# Supplementary material for: SARS-CoV-2 infection in central North Carolina: Protocol for a population-based longitudinal cohort study and preliminary participant results
Source: PLoS One. 2021 Oct 25;16(10):e0259070. doi: 10.1371/journal.pone.0259070 (PMC8544868; doi:10.1371/journal.pone.0259070)
Supplement: S1 Table — Sources: Percent of households under federal poverty rate are from 2019 U.S. Census Small Area Income and Poverty Estimates (SAIPE). Race and ethnicity are from 2019 American Community Survey (ACS) 5-year estimates, whereas all other demographics use 2019 ACS 1-year estimates. (DOCX) [file pone.0259070.s001.docx]

| **S1 Table. Demographics of Chatham County and North Carolina (2019)** | | | |
| --- | --- | --- | --- |
| **Characteristic** | | Chatham County | North Carolina |
| Total Population | | 74,470 | 10,488,084 |
| Sex | |  |  |
|  | Female | 37,328 (52.3%) | 5,393,757 (51.4%) |
|  | Male | 34,010 (47.7%) | 5,094,327 (48.6%) |
| Median Age (years) | | 46.7 | 39.1 |
| Age by Category (years) | |  |  |
|  | <20 | 15,766 (22.2%) | 2,611,879 (24.9%) |
|  | 20-34 | 9,495 (13.3%) | 2,090,791 (20.0%) |
|  | 35-44 | 8,365 (11.7%) | 1,312,661 (12.5%) |
|  | 45-54 | 10,007 (14.0%) | 1,363,200 (13.0%) |
|  | 55-64 | 10,576 (14.8%) | 1,358,618 (16.3%) |
|  | 65+ | 17,129 (24.0%) | 1,750,935 (16.7%) |
| Race & Ethnicity* | |  |  |
|  | White | 51,146 (71.7%) | 6,474,688 (63.1%) |
|  | Hispanic or Latino | 8,707 (12.2%) | 962,665 (9.4%) |
|  | Black or African American | 7,898 (11.1%) | 2,165,301 (21.1%) |
|  | Asian | 1,068 (1.5%) | 290,525 (2.8%) |
|  | Other | 384 (0.5%) | 22,962 (0.2%) |
|  | American Indian or Alaska Native | 63 (0.1%) | 112,504 (1.1%) |
|  | Native Hawaiian or Other Pacific Islander | 48 (0.1%) | 5,640 (0.1%) |
|  | Two or more races | 2,024 (2.8%) | 230,591 (2.3%) |
| Median Household Income | | $66,857 | $57,341 |
| Households Under Federal Poverty Rate | | 8.7% | 13.6% |
| Household Income | |  |  |
|  | Under $50K | 38.9% | 43.9% |
|  | $50K – $100K | 28.0% | 30.7% |
|  | $100K– $200K | 22.3% | 19.4% |
|  | Over $200K | 10.8% | 6.0% |
| Educational Attainment | |  |  |
|  | No degree | 7,039 (12.8%) | 818,924 (11.4%) |
|  | High school graduate | 11,838 (21.5%) | 1,839,042 (25.6%) |
|  | Some college | 11,942 (21.7%) | 2,207,926 (30.7%) |
|  | Bachelor’s degree or higher | 24,223 (44.0%) | 2,321,185 (32.3%) |
