# Supplementary figures and images for: SARS-CoV-2 infection in central North Carolina: Protocol for a population-based longitudinal cohort study and preliminary participant results
Source: PLoS One. 2021 Oct 25;16(10):e0259070. doi: 10.1371/journal.pone.0259070 (PMC8544868; doi:10.1371/journal.pone.0259070)

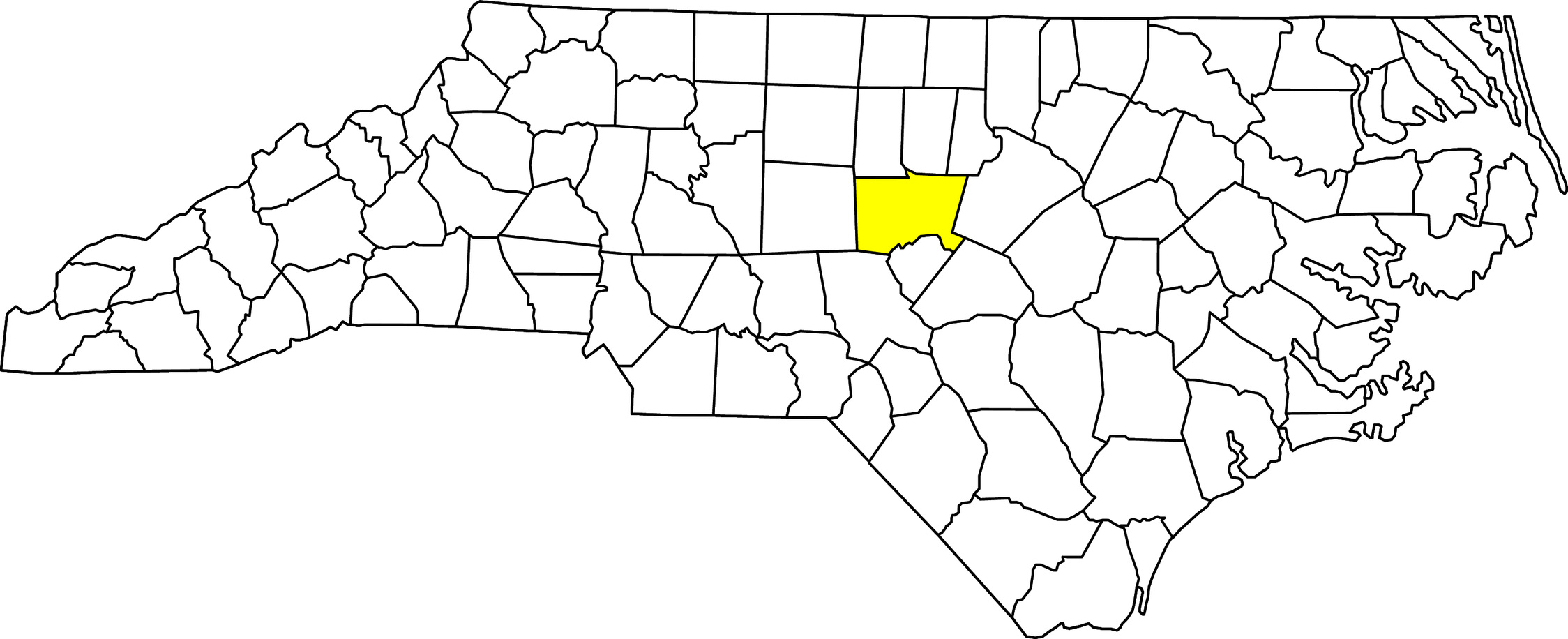

Supplement: S1 Fig — (TIF) [file pone.0259070.s002.tif]

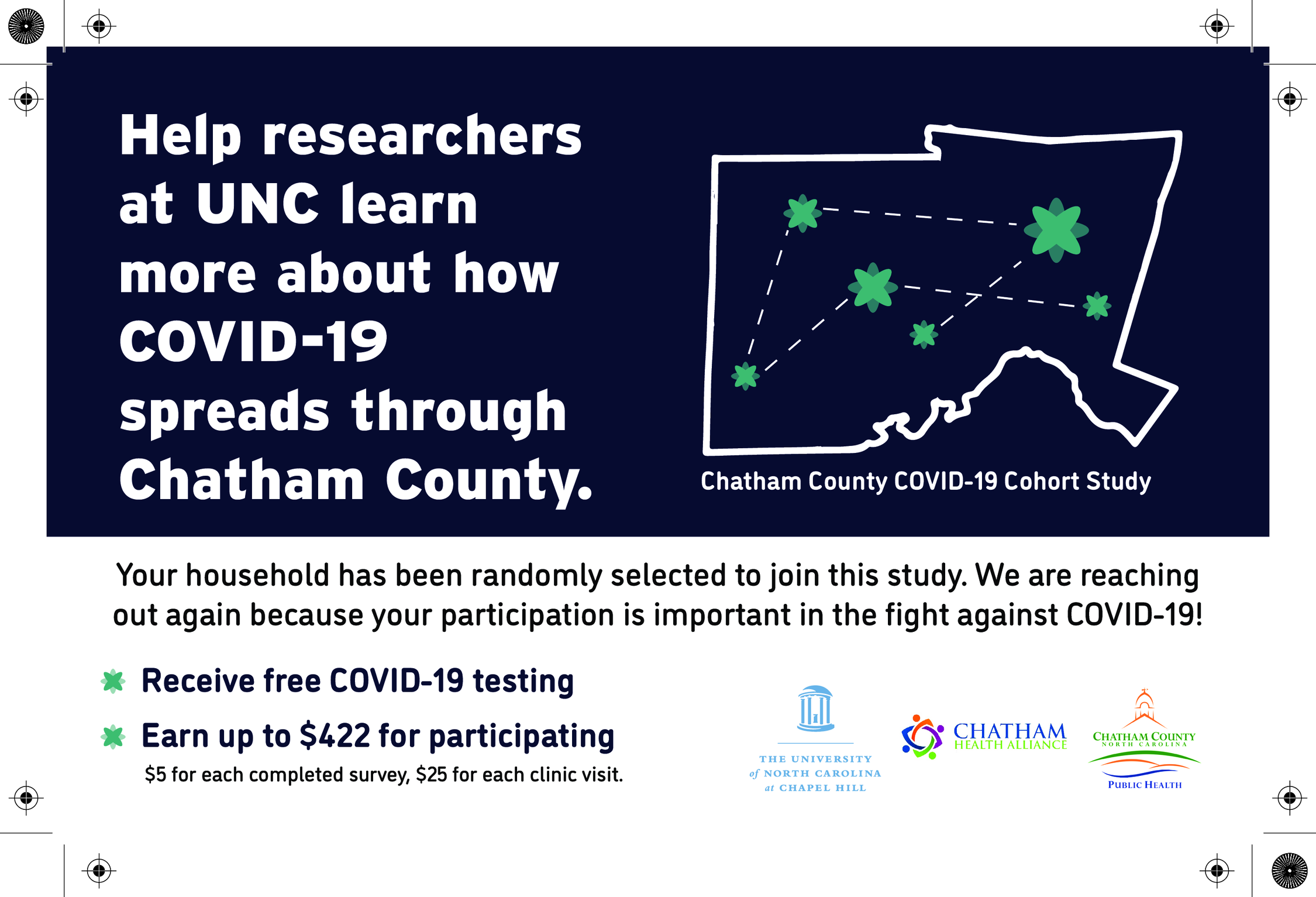

Supplement: S2 Fig — (TIF) [file pone.0259070.s003.tif]
