# Supplementary material for: SARS-CoV-2 infection in central North Carolina: Protocol for a population-based longitudinal cohort study and preliminary participant results
Source: PLoS One. 2021 Oct 25;16(10):e0259070. doi: 10.1371/journal.pone.0259070 (PMC8544868; doi:10.1371/journal.pone.0259070)
Supplement: S2 Appendix — (PDF) [file pone.0259070.s005.pdf]

Bi-weekly Survey

This Chatham County COVID-19 Cohort Study is being conducted by researchers from the University of North Carolina at Chapel Hill Schools of Medicine and Public Health. The purpose of this study is to learn how the new coronavirus disease, called COVID-19, is spreading and how it affects health. The results may help uncover new ways to prevent COVID-19 in the future.

Please complete the survey below.

Thank you!

Would you prefer to take this survey in English or Spanish? / Preferie responder esta encuesta en ingles o espanol?

- ◯ English / ingles
- ◯ Spanish / espanol

Which of the following best fits your current work situation?

- ◯ works full time
- ◯ works part time
- ◯ is looking for work/employment
- ◯ retired
- ◯ homemaker
- ◯ student
- ◯ on maternity/paternity leave
- ◯ on illness/sick leave
- ◯ on disability
- ◯ other

Do you currently consider yourself self-employed (including as an independent contractor or gig-economy worker)?

- ◯ yes
- ◯ no
- ◯ don't know

On a scale of 0 (definitely not going to happen) to 10 (definitely going to happen), how likely is it that you will lose your job because of the COVID-19 pandemic?

____

On a scale of 0 (definitely not going to happen) to 10 (definitely going to happen), how likely is it that you will receive fewer work hours at your job because of the COVID-19 pandemic?

____

On a scale of 0 (definitely not going to happen) to 10 (definitely going to happen), how likely do you think it is that your household will run out of money in the next 3 months?

____

---

	always (100%)	most of the time (75%)	half of the time (50%)	less than half of the time (25%)	never (0%)
How often are you required to work from outside of the home currently?	◯	◯	◯	◯	◯

How regularly are you in close physical contact with co-workers during your work outside of the home currently?	○	○	○	○	○
How regularly are you in close physical contact with clients during your work outside of the home currently?	○	○	○	○	○
How often do you have access to disposable gloves during your work outside of the home currently?	○	○	○	○	○
How often do you have access to a face mask during your work outside of the home currently?	○	○	○	○	○
How often do you use disposable gloves during your work outside of the home currently?	○	○	○	○	○
How often do you use a face mask during your work outside of the home currently?	○	○	○	○	○
How often do you wash your hands with soap and water during your work outside of the home currently?	○	○	○	○	○
How often do you sanitize your hands with hand sanitizer during your work outside of the home currently?	○	○	○	○	○
How worried are you that you will be exposed to COVID-19 during your work outside of the home currently?	○	○	○	○	○

Do you currently work in any of the following high-risk settings for COVID-19 transmission?

- ☐ healthcare setting (hospital, clinic, urgent care, etc.)
- ☐ dense residential setting (nursing home, other long-term care facility)
- ☐ prison or jail
- ☐ meatpacking facility
- ☐ shipping or distribution facility
- ☐ high-volume retail facility (grocery store, etc.)

Are you covered by any type of medical or health insurance (including private insurance, insurance your purchased, Medicare, Medicaid, or any other health insurance program)?

- ○ yes
- ○ no
- ○ don't know

What is the primary health insurance coverage that you have?	◯ Private health insurance through a job or school<br>◯ Insurance purchased through a state or federal health insurance exchange, such as healthcare.gov<br>◯ Insurance purchased directly through a health plan or insurance company<br>◯ Medicare<br>◯ Medi-Gap<br>◯ Medicaid<br>◯ Military health care (TRICARE, VA, CHAMP-VA, etc.)<br>◯ Indian Health Service<br>◯ Other<br>(Select one (your primary insurance).)
Please specify your other source of health insurance	__________________________________
Are you currently pregnant?	◯ Yes<br>◯ No

	excellent	very good	good	fair	poor
In general, how would you rate your health over the last two weeks?	◯	◯	◯	◯	◯

How often have you done the following things to protect yourself from infection during the last two weeks?

	always (100%)	most of the time (75%)	half of the time (50%)	less than half of the time (25%)	never (0%)
Worn a face mask	◯	◯	◯	◯	◯
Washed hands and/or used sanitizer frequently	◯	◯	◯	◯	◯
Stayed at least 6 feet away from others	◯	◯	◯	◯	◯
Avoided large gatherings, public spaces, or crowds	◯	◯	◯	◯	◯
Avoided contact with people who could be high risk	◯	◯	◯	◯	◯
Avoided food from restaurants, including takeout	◯	◯	◯	◯	◯
Worked or studied at home instead of going into an office/classroom	◯	◯	◯	◯	◯
Avoided shaking hands or touching people	◯	◯	◯	◯	◯
Stayed home when you were sick	◯	◯	◯	◯	◯
Wiped down surfaces with disinfectant	◯	◯	◯	◯	◯

Cancelled or postponed planned travel for work	○	○	○	○	○
Cancelled or postponed travel for pleasure	○	○	○	○	○
Cancelled or postponed personal or social activities	○	○	○	○	○
Cancelled a doctor's appointment	○	○	○	○	○
Stockpiled food or water	○	○	○	○	○
Followed government guidelines or rules to shelter in place (staying at home, limiting contacts with other people, etc.)	○	○	○	○	○

During the last two weeks, have you experienced any of the following symptoms?

	yes	no
Fever (measured by thermometer or self-diagnosed)	○	○
Cough (new or worsening)	○	○
Shortness of breath (new or worsening)	○	○
Fatigue (new tiredness doing normal activities)	○	○
Body aches	○	○
Headache	○	○
Diarrhea	○	○
Sore throat	○	○
Itchy, pink, or painful eyes	○	○
Runny nose or congestion	○	○
Changes in your sense of smell or taste	○	○
New rash	○	○
Repeated shaking with chills	○	○

When did the symptoms reported above first start?

____

Did you experience any bias or discrimination due to the symptoms you reported?

- ○ yes
- ○ no
- ○ don't know

Which of the following did you do to protect your friends and family after your symptoms began?

- ☐ wore a mask more frequently
- ☐ washed your hands with soap and water more frequently
- ☐ used hand sanitizer more frequently
- ☐ isolated yourself in your home more frequently
- ☐ stayed home more frequently
- ☐ wore disposable gloves more frequently

What did you do in response to the symptoms reported above?	☐ nothing<br>☐ took over the counter medication (ibuprofen, acetaminophen, etc.)<br>☐ communicated with a health care provider over the phone<br>☐ visited a health care provider's office<br>☐ visited a retail clinic or pharmacy<br>☐ visited urgent care (FASTMed, etc.)<br>☐ visited the emergency room<br>☐ was admitted to the hospital<br>☐ other<br>(Select all that apply.)
Please specify what other action you took in response to your symptoms.	__________________________________
If you were able to talk with a health care provider, were you told that you may have COVID-19?	◯ yes<br>◯ no<br>◯ don't know
If you received a COVID-19 test due to the symptoms you reported, what was the result?	◯ pending<br>◯ positive<br>◯ negative<br>◯ inconclusive<br>◯ did not receive a test
How many days were you admitted to the hospital?	__________________________________
Did you receive the following interventions during your hospital admission?	☐ extra oxygen in your nose<br>☐ treatment in the intensive care unit (ICU)<br>☐ mechanical ventilation (intubation or a breathing tube)
Have you returned to your normal health at this time?	◯ yes<br>◯ no<br>◯ don't know
Have you ever been, or are currently, in a COVID vaccine trial?	◯ Yes<br>◯ No
Where was/is the primary site for the COVID vaccine trial? (For example: UNC, Duke, a specific hospital?)	__________________________________
Have you received a COVID vaccine outside of a clinical trial?	◯ Yes<br>◯ No
Where did you receive the COVID vaccine?	◯ Doctors Office<br>◯ Work/Employment<br>◯ Retail (e.g. Walgreens, CVS)<br>◯ Vaccine site<br>◯ Other
Please specify where you received the COVID vaccine.	__________________________________
Please specify the city/town in NC of the vaccine site:	__________________________________

Please specify who was the hosting organization/institution of the vaccine site: __________________________________

Which COVID vaccine did you receive?
- ◯ Pfizer
- ◯ Moderna
- ◯ AstraZeneca
- ◯ Novavax
- ◯ Johnson & Johnson
- ◯ Other
- ◯ Unsure/Unknown

Please specify which other COVID vaccine you received. __________________________________

How many doses of the COVID vaccine have you received?
- ◯ 1
- ◯ 2

Which day did you receive the first dose of the vaccine? __________________________________

Which day did you receive the second dose of the vaccine? __________________________________

Did you experience any of the following side effects after vaccination?

		No	Mild (you notice symptoms, but they aren't a problem)	Moderate (symptoms limit your normal daily activities)	Severe (symptoms make normal daily activities difficult or impossible)
1	pain at or around the injection site	◯	◯	◯	◯
2	redness at or around the injection site	◯	◯	◯	◯
3	swelling at or around the injection site	◯	◯	◯	◯
4	rash at or around the injection site	◯	◯	◯	◯
5	headache	◯	◯	◯	◯
6	fatigue	◯	◯	◯	◯
7	fever (temperature >100.4°F or >38°C)	◯	◯	◯	◯
8	chills	◯	◯	◯	◯
9	joint pain	◯	◯	◯	◯
10	muscle pain	◯	◯	◯	◯
11	nausea	◯	◯	◯	◯

How long did these side effects last?
- ◯ Less than 12 hours
- ◯ 12 to 24 hours
- ◯ more than 24 hours

Did you take any medication for these side effects?
- ◯ Yes
- ◯ No

What medication(s) did you take for the side effects?

____
(Please list all medications.)

Did you consult a physician or other health care provider for the side effects?

◯ Yes
◯ No

How did you experience the side effects after the second dose of the vaccination as compared to those after the first dose of the vaccination?

◯ More severe
◯ Less severe
◯ Equally severe
◯ Not applicable/Haven't received second dose yet

Please provide the following information about your household.

What is your permanent address?

____

How long have you lived at this address?

◯ 0-3 years
◯ 4-6 years
◯ 7-10 years
◯ more than 10 years

How many additional people (not including yourself) live or spend a significant amount of time (greater than 40 hours a week) in this household?

◯ 0
◯ 1
◯ 2
◯ 3
◯ 4
◯ 5
◯ 6
◯ 7
◯ 8
◯ 9
◯ 10
◯ 11
◯ 12

How many of the people in your household are below the age of 18?

◯ 0
◯ 1
◯ 2
◯ 3
◯ 4
◯ 5
◯ 6
◯ 7
◯ 8
◯ 9
◯ 10
◯ 11
◯ 12

For each additional person in the your household, please provide the following information.

Person 1: What is your relationship to this person?	◯ partner or spouse<br>◯ child<br>◯ parent<br>◯ sibling<br>◯ other family member<br>◯ in-home childcare provider or other caregiver<br>◯ other
Person 1: Please specify your relationship with this person.	__________________________
Person 1: What is this person's age?	__________________________<br>(Please specify their age in years)
Person 1: What is this person's sex?	◯ Female<br>◯ Male<br>◯ Other
Person 1: What is this person's race?	☐ American Indian or Alaska Native<br>☐ Asian<br>☐ Black or African American<br>☐ Native Hawaiian or Pacific Islander<br>☐ White<br>☐ Other<br>☐ don't know<br>(Select all that apply.)
Person 1: What is this person's ethnicity?	◯ Hispanic or Latino<br>◯ Not Hispanic or Latino<br>◯ Other<br>◯ don't know
Person 1: What is the highest level of education or schooling this person has completed?	◯ never attended school<br>◯ kindergarten - 8th grade<br>◯ some high school<br>◯ high school equivalency (GED)<br>◯ high school graduate<br>◯ some college<br>◯ college graduate<br>◯ graduate school or more<br>◯ don't know
Person 1: Which of the following best fit this person's current work situation?	◯ works full time<br>◯ works part time<br>◯ is looking for work/employment<br>◯ retired<br>◯ homemaker<br>◯ student<br>◯ on maternity/paternity leave<br>◯ on illness/sick leave<br>◯ on disability<br>◯ other<br>◯ don't know
Person 1: Does this person currently consider themselves self-employed (including as an independent contractor or gig-economy worker)?	◯ yes<br>◯ no<br>◯ don't know

Person 1: Does this person currently work in any of the following high-risk settings for COVID-19 transmission?

- ☐ healthcare setting (hospital, clinic, urgent care, etc.)
- ☐ dense residential setting (nursing home, other long-term care facility)
- ☐ prison or jail
- ☐ meatpacking facility
- ☐ shipping or distribution facility
- ☐ high-volume retail facility (grocery store, etc.)
- ☐ don't know

Person 1: Does this person's employer offer them any of the following benefits at their current main job?

- ☐ paid sick leave
- ☐ paid vacation/personal leave
- ☐ health insurance
- ☐ disability insurance
- ☐ retirement plan
- ☐ other
- ☐ don't know

(Select all that apply.)

Person 1: On a scale of 0 (definitely not going to happen) to 10 (definitely going to happen), how likely is it that this person will lose their job because of the COVID-19 pandemic?

____

Person 1: On a scale of 0 (definitely not going to happen) to 10 (definitely going to happen), how likely is it that this person will receive fewer work hours at their job because of the COVID-19 pandemic?

____

	always (100%)	most of the time (75%)	half of the time (50%)	less than half of the time (25%)	never (0%)	don't know
Person 1: How often is this person required to work from outside of the home currently?	◯	◯	◯	◯	◯	◯
Person 1: How regularly is this person in close physical contact with co-workers during their work outside of the home currently?	◯	◯	◯	◯	◯	◯
Person 1: How regularly is this person in close physical contact with clients during their work outside of the home currently?	◯	◯	◯	◯	◯	◯

Person 1: Does this person plan to get a vaccine for COVID-19 when one becomes available?

- ◯ yes
- ◯ no
- ◯ don't know

Person 1: Has this person had any symptoms (cough, fever, difficulty breathing, fatigue, body aches, diarrhea, runny nose, loss of smell or taste) consistent with COVID-19 in the last two weeks?

- ◯ yes
- ◯ no
- ◯ don't know

Person 1: When did this person's symptoms begin?

____

Person 1: Is this person worried that they may have had COVID-19 because of their symptoms?	◯ yes<br>◯ no<br>◯ don't know
Person 1: Did this person experience any bias or discrimination because of their symptoms?	◯ yes<br>◯ no<br>◯ don't know
Person 1: What did this person do in response to their symptoms?	☐ nothing<br>☐ took over the counter medication (ibuprofen, acetaminophen, etc.)<br>☐ communicated with a health care provider over the phone<br>☐ visited a health care provider's office<br>☐ visited a retail clinic or pharmacy<br>☐ visited urgent care (FASTMed, etc.)<br>☐ visited the emergency room<br>☐ was admitted to the hospital<br>☐ other<br>☐ don't know<br>(Select all that apply.)
Person 1: Please specify what other action this person took in response to their symptoms.	______________________
Person 1: Did a health care provider tell this person that they may have COVID-19?	◯ yes<br>◯ no<br>◯ don't know
Person 1: If this person received a COVID-19 test due to their symptoms, what was the result?	◯ pending<br>◯ positive<br>◯ negative<br>◯ inconclusive<br>◯ did not receive a test<br>◯ don't know
Person 1: How many days was this person admitted to the hospital?	______________________
Person 1: Did this person receive any of the following interventions during their hospital admission?	☐ extra oxygen in your nose<br>☐ treatment in the intensive care unit (ICU)<br>☐ mechanical ventilation (intubation or a breathing tube)<br>☐ don't know
Person 1: Has this person returned to their normal health at this time?	◯ yes<br>◯ no<br>◯ don't know
Person 1: Which of the following did this person do to protect their friends and family after their symptoms began?	☐ wore a mask more frequently<br>☐ washed your hands with soap and water more frequently<br>☐ used hand sanitizer more frequently<br>☐ isolated yourself in your home more frequently<br>☐ stayed home more frequently<br>☐ wore disposable gloves more frequently<br>☐ don't know

For each additional person in the your household, please provide the following information.

Person 2: What is your relationship to this person?

- ◯ partner or spouse
- ◯ child
- ◯ parent
- ◯ sibling
- ◯ other family member
- ◯ in-home childcare provider or other caregiver
- ◯ other

Person 2: Please specify your relationship with this person.

____

Person 2: What is this person's age?

____
(Please specify their age in years)

Person 2: What is this person's sex?

- ◯ Female
- ◯ Male
- ◯ Other

Person 2: What is this person's race?

- ☐ American Indian or Alaska Native
- ☐ Asian
- ☐ Black or African American
- ☐ Native Hawaiian or Pacific Islander
- ☐ White
- ☐ Other
- ☐ don't know

(Select all that apply.)

Person 2: What is this person's ethnicity?

- ◯ Hispanic or Latino
- ◯ Not Hispanic or Latino
- ◯ Other
- ◯ don't know

Person 2: What is the highest level of education or schooling this person has completed?

- ◯ never attended school
- ◯ kindergarten - 8th grade
- ◯ some high school
- ◯ high school equivalency (GED)
- ◯ high school graduate
- ◯ some college
- ◯ college graduate
- ◯ graduate school or more
- ◯ don't know

Person 2: Which of the following best fit this person's current work situation?

- ◯ works full time
- ◯ works part time
- ◯ is looking for work/employment
- ◯ retired
- ◯ homemaker
- ◯ student
- ◯ on maternity/paternity leave
- ◯ on illness/sick leave
- ◯ on disability
- ◯ other
- ◯ don't know

Person 2: Does this person currently consider themselves self-employed (including as an independent contractor or gig-economy worker)?

- ◯ yes
- ◯ no
- ◯ don't know

Person 2: Does this person currently work in any of the following high-risk settings for COVID-19 transmission?	☐ healthcare setting (hospital, clinic, urgent care, etc.)<br>☐ dense residential setting (nursing home, other long-term care facility)<br>☐ prison or jail<br>☐ meatpacking facility<br>☐ shipping or distribution facility<br>☐ high-volume retail facility (grocery store, etc.)<br>☐ don't know
Person 2: Does this person's employer offer them any of the following benefits at their current main job?	☐ paid sick leave<br>☐ paid vacation/personal leave<br>☐ health insurance<br>☐ disability insurance<br>☐ retirement plan<br>☐ other<br>☐ don't know<br>(Select all that apply.)
Person 2: On a scale of 0 (definitely not going to happen) to 10 (definitely going to happen), how likely is it that this person will lose their job because of the COVID-19 pandemic?	__________________________________
Person 2: On a scale of 0 (definitely not going to happen) to 10 (definitely going to happen), how likely is it that this person will receive fewer work hours at their job because of the COVID-19 pandemic?	__________________________________

	always (100%)	most of the time (75%)	half of the time (50%)	less than half of the time (25%)	never (0%)	don't know
Person 2: How often is this person required to work from outside of the home currently?	○	○	○	○	○	○
Person 2: How regularly is this person in close physical contact with co-workers during their work outside of the home currently?	○	○	○	○	○	○
Person 2: How regularly is this person in close physical contact with clients during their work outside of the home currently?	○	○	○	○	○	○

Person 2: Does this person plan to get a vaccine for COVID-19 when one becomes available?	○ yes<br>○ no<br>○ don't know
Person 2: Has this person had any symptoms (cough, fever, difficulty breathing, fatigue, body aches, diarrhea, runny nose, loss of smell or taste) consistent with COVID-19 in the last two weeks?	○ yes<br>○ no<br>○ don't know
Person 2: When did this person's symptoms begin?	__________________________________

Person 2: Is this person worried that they may have had COVID-19 because of their symptoms?	◯ yes<br>◯ no<br>◯ don't know
Person 2: Did this person experience any bias or discrimination because of their symptoms?	◯ yes<br>◯ no<br>◯ don't know
Person 2: What did this person do in response to their symptoms?	☐ nothing<br>☐ took over the counter medication (ibuprofen, acetaminophen, etc.)<br>☐ communicated with a health care provider over the phone<br>☐ visited a health care provider's office<br>☐ visited a retail clinic or pharmacy<br>☐ visited urgent care (FASTMed, etc.)<br>☐ visited the emergency room<br>☐ was admitted to the hospital<br>☐ other<br>☐ don't know<br>(Select all that apply.)
Person 2: Please specify what other action this person took in response to their symptoms.	__________________________________
Person 2: Did a health care provider tell this person that they may have COVID-19?	◯ yes<br>◯ no<br>◯ don't know
Person 2: If this person received a COVID-19 test due to their symptoms, what was the result?	◯ pending<br>◯ positive<br>◯ negative<br>◯ inconclusive<br>◯ did not receive a test<br>◯ don't know
Person 2: How many days was this person admitted to the hospital?	__________________________________
Person 2: Did this person receive any of the following interventions during their hospital admission?	☐ extra oxygen in your nose<br>☐ treatment in the intensive care unit (ICU)<br>☐ mechanical ventilation (intubation or a breathing tube)<br>☐ don't know
Person 2: Has this person returned to their normal health at this time?	◯ yes<br>◯ no<br>◯ don't know
Person 2: Which of the following did this person do to protect their friends and family after their symptoms began?	☐ wore a mask more frequently<br>☐ washed your hands with soap and water more frequently<br>☐ used hand sanitizer more frequently<br>☐ isolated yourself in your home more frequently<br>☐ stayed home more frequently<br>☐ wore disposable gloves more frequently<br>☐ don't know

For each additional person in the your household, please provide the following information.

Person 3: What is your relationship to this person?
- ◯ partner or spouse
- ◯ child
- ◯ parent
- ◯ sibling
- ◯ other family member
- ◯ in-home childcare provider or other caregiver
- ◯ other

Person 3: Please specify your relationship with this person.

____

Person 3: What is this person's age?

____
(Please specify their age in years)

Person 3: What is this person's sex?
- ◯ Female
- ◯ Male
- ◯ Other

Person 3: What is this person's race?
- ☐ American Indian or Alaska Native
- ☐ Asian
- ☐ Black or African American
- ☐ Native Hawaiian or Pacific Islander
- ☐ White
- ☐ Other
- ☐ don't know

(Select all that apply.)

Person 3: What is this person's ethnicity?
- ◯ Hispanic or Latino
- ◯ Not Hispanic or Latino
- ◯ Other
- ◯ don't know

Person 3: What is the highest level of education or schooling this person has completed?
- ◯ never attended school
- ◯ kindergarten - 8th grade
- ◯ some high school
- ◯ high school equivalency (GED)
- ◯ high school graduate
- ◯ some college
- ◯ college graduate
- ◯ graduate school or more
- ◯ don't know

Person 3: Which of the following best fit this person's current work situation?
- ◯ works full time
- ◯ works part time
- ◯ is looking for work/employment
- ◯ retired
- ◯ homemaker
- ◯ student
- ◯ on maternity/paternity leave
- ◯ on illness/sick leave
- ◯ on disability
- ◯ other
- ◯ don't know

Person 3: Does this person currently consider themselves self-employed (including as an independent contractor or gig-economy worker)?
- ◯ yes
- ◯ no
- ◯ don't know

Person 3: Does this person currently work in any of the following high-risk settings for COVID-19 transmission?

- ☐ healthcare setting (hospital, clinic, urgent care, etc.)
- ☐ dense residential setting (nursing home, other long-term care facility)
- ☐ prison or jail
- ☐ meatpacking facility
- ☐ shipping or distribution facility
- ☐ high-volume retail facility (grocery store, etc.)
- ☐ don't know

Person 3: Does this person's employer offer them any of the following benefits at their current main job?

- ☐ paid sick leave
- ☐ paid vacation/personal leave
- ☐ health insurance
- ☐ disability insurance
- ☐ retirement plan
- ☐ other
- ☐ don't know

(Select all that apply.)

Person 3: On a scale of 0 (definitely not going to happen) to 10 (definitely going to happen), how likely is it that this person will lose their job because of the COVID-19 pandemic?

____

Person 3: On a scale of 0 (definitely not going to happen) to 10 (definitely going to happen), how likely is it that this person will receive fewer work hours at their job because of the COVID-19 pandemic?

____

	always (100%)	most of the time (75%)	half of the time (50%)	less than half of the time (25%)	never (0%)	don't know
Person 3: How often is this person required to work from outside of the home currently?	◯	◯	◯	◯	◯	◯
Person 3: How regularly is this person in close physical contact with co-workers during their work outside of the home currently?	◯	◯	◯	◯	◯	◯
Person 3: How regularly is this person in close physical contact with clients during their work outside of the home currently?	◯	◯	◯	◯	◯	◯

Person 3: Does this person plan to get a vaccine for COVID-19 when one becomes available?

- ◯ yes
- ◯ no
- ◯ don't know

Person 3: Has this person had any symptoms (cough, fever, difficulty breathing, fatigue, body aches, diarrhea, runny nose, loss of smell or taste) consistent with COVID-19 in the last two weeks?

- ◯ yes
- ◯ no
- ◯ don't know

Person 3: When did this person's symptoms begin?

____

Person 3: Is this person worried that they may have had COVID-19 because of their symptoms?	◯ yes<br>◯ no<br>◯ don't know
Person 3: Did this person experience any bias or discrimination because of their symptoms?	◯ yes<br>◯ no<br>◯ don't know
Person 3: What did this person do in response to their symptoms?	☐ nothing<br>☐ took over the counter medication (ibuprofen, acetaminophen, etc.)<br>☐ communicated with a health care provider over the phone<br>☐ visited a health care provider's office<br>☐ visited a retail clinic or pharmacy<br>☐ visited urgent care (FASTMed, etc.)<br>☐ visited the emergency room<br>☐ was admitted to the hospital<br>☐ other<br>☐ don't know<br>(Select all that apply.)
Person 3: Please specify what other action this person took in response to their symptoms.	______________________________
Person 3: Did a health care provider tell this person that they may have COVID-19?	◯ yes<br>◯ no<br>◯ don't know
Person 3: If this person received a COVID-19 test due to their symptoms, what was the result?	◯ pending<br>◯ positive<br>◯ negative<br>◯ inconclusive<br>◯ did not receive a test<br>◯ don't know
Person 3: How many days was this person admitted to the hospital?	______________________________
Person 3: Did this person receive any of the following interventions during their hospital admission?	☐ extra oxygen in your nose<br>☐ treatment in the intensive care unit (ICU)<br>☐ mechanical ventilation (intubation or a breathing tube)<br>☐ don't know
Person 3: Has this person returned to their normal health at this time?	◯ yes<br>◯ no<br>◯ don't know
Person 3: Which of the following did this person do to protect their friends and family after their symptoms began?	☐ wore a mask more frequently<br>☐ washed your hands with soap and water more frequently<br>☐ used hand sanitizer more frequently<br>☐ isolated yourself in your home more frequently<br>☐ stayed home more frequently<br>☐ wore disposable gloves more frequently<br>☐ don't know

For each additional person in the your household, please provide the following information.

Person 4: What is your relationship to this person?	◯ partner or spouse<br>◯ child<br>◯ parent<br>◯ sibling<br>◯ other family member<br>◯ in-home childcare provider or other caregiver<br>◯ other
Person 4: Please specify your relationship with this person.	________________________________
Person 4: What is this person's age?	________________________________<br>(Please specify their age in years)
Person 4: What is this person's sex?	◯ Female<br>◯ Male<br>◯ Other
Person 4: What is this person's race?	☐ American Indian or Alaska Native<br>☐ Asian<br>☐ Black or African American<br>☐ Native Hawaiian or Pacific Islander<br>☐ White<br>☐ Other<br>☐ don't know<br>(Select all that apply.)
Person 4: What is this person's ethnicity?	◯ Hispanic or Latino<br>◯ Not Hispanic or Latino<br>◯ Other<br>◯ don't know
Person 4: What is the highest level of education or schooling this person has completed?	◯ never attended school<br>◯ kindergarten - 8th grade<br>◯ some high school<br>◯ high school equivalency (GED)<br>◯ high school graduate<br>◯ some college<br>◯ college graduate<br>◯ graduate school or more<br>◯ don't know
Person 4: Which of the following best fit this person's current work situation?	◯ works full time<br>◯ works part time<br>◯ is looking for work/employment<br>◯ retired<br>◯ homemaker<br>◯ student<br>◯ on maternity/paternity leave<br>◯ on illness/sick leave<br>◯ on disability<br>◯ other<br>◯ don't know
Person 4: Does this person currently consider themselves self-employed (including as an independent contractor or gig-economy worker)?	◯ yes<br>◯ no<br>◯ don't know

Person 4: Does this person currently work in any of the following high-risk settings for COVID-19 transmission?	☐ healthcare setting (hospital, clinic, urgent care, etc.)<br>☐ dense residential setting (nursing home, other long-term care facility)<br>☐ prison or jail<br>☐ meatpacking facility<br>☐ shipping or distribution facility<br>☐ high-volume retail facility (grocery store, etc.)<br>☐ don't know
Person 4: Does this person's employer offer them any of the following benefits at their current main job?	☐ paid sick leave<br>☐ paid vacation/personal leave<br>☐ health insurance<br>☐ disability insurance<br>☐ retirement plan<br>☐ other<br>☐ don't know<br>(Select all that apply.)
Person 4: On a scale of 0 (definitely not going to happen) to 10 (definitely going to happen), how likely is it that this person will lose their job because of the COVID-19 pandemic?	__________________________
Person 4: On a scale of 0 (definitely not going to happen) to 10 (definitely going to happen), how likely is it that this person will receive fewer work hours at their job because of the COVID-19 pandemic?	__________________________

	always (100%)	most of the time (75%)	half of the time (50%)	less than half of the time (25%)	never (0%)	don't know
Person 4: How often is this person required to work from outside of the home currently?	○	○	○	○	○	○
Person 4: How regularly is this person in close physical contact with co-workers during their work outside of the home currently?	○	○	○	○	○	○
Person 4: How regularly is this person in close physical contact with clients during their work outside of the home currently?	○	○	○	○	○	○

Person 4: Does this person plan to get a vaccine for COVID-19 when one becomes available?	○ yes<br>○ no<br>○ don't know
Person 4: Has this person had any symptoms (cough, fever, difficulty breathing, fatigue, body aches, diarrhea, runny nose, loss of smell or taste) consistent with COVID-19 in the last two weeks?	○ yes<br>○ no<br>○ don't know
Person 4: When did this person's symptoms begin?	__________________________

Person 4: Is this person worried that they may have had COVID-19 because of their symptoms?	◯ yes<br>◯ no<br>◯ don't know
Person 4: Did this person experience any bias or discrimination because of their symptoms?	◯ yes<br>◯ no<br>◯ don't know
Person 4: What did this person do in response to their symptoms?	☐ nothing<br>☐ took over the counter medication (ibuprofen, acetaminophen, etc.)<br>☐ communicated with a health care provider over the phone<br>☐ visited a health care provider's office<br>☐ visited a retail clinic or pharmacy<br>☐ visited urgent care (FASTMed, etc.)<br>☐ visited the emergency room<br>☐ was admitted to the hospital<br>☐ other<br>☐ don't know<br>(Select all that apply.)
Person 4: Please specify what other action this person took in response to their symptoms.	__________________________________
Person 4: Did a health care provider tell this person that they may have COVID-19?	◯ yes<br>◯ no<br>◯ don't know
Person 4: If this person received a COVID-19 test due to their symptoms, what was the result?	◯ pending<br>◯ positive<br>◯ negative<br>◯ inconclusive<br>◯ did not receive a test<br>◯ don't know
Person 4: How many days was this person admitted to the hospital?	__________________________________
Person 4: Did this person receive any of the following interventions during their hospital admission?	☐ extra oxygen in your nose<br>☐ treatment in the intensive care unit (ICU)<br>☐ mechanical ventilation (intubation or a breathing tube)<br>☐ don't know
Person 4: Has this person returned to their normal health at this time?	◯ yes<br>◯ no<br>◯ don't know
Person 4: Which of the following did this person do to protect their friends and family after their symptoms began?	☐ wore a mask more frequently<br>☐ washed your hands with soap and water more frequently<br>☐ used hand sanitizer more frequently<br>☐ isolated yourself in your home more frequently<br>☐ stayed home more frequently<br>☐ wore disposable gloves more frequently<br>☐ don't know

For each additional person in the your household, please provide the following information.

Person 5: What is your relationship to this person?	◯ partner or spouse<br>◯ child<br>◯ parent<br>◯ sibling<br>◯ other family member<br>◯ in-home childcare provider or other caregiver<br>◯ other
Person 5: Please specify your relationship with this person.	__________________________________
Person 5: What is this person's age?	__________________________________<br>(Please specify their age in years)
Person 5: What is this person's sex?	◯ Female<br>◯ Male<br>◯ Other
Person 5: What is this person's race?	☐ American Indian or Alaska Native<br>☐ Asian<br>☐ Black or African American<br>☐ Native Hawaiian or Pacific Islander<br>☐ White<br>☐ Other<br>☐ don't know<br>(Select all that apply.)
Person 5: What is this person's ethnicity?	◯ Hispanic or Latino<br>◯ Not Hispanic or Latino<br>◯ Other<br>◯ don't know
Person 5: What is the highest level of education or schooling this person has completed?	◯ never attended school<br>◯ kindergarten - 8th grade<br>◯ some high school<br>◯ high school equivalency (GED)<br>◯ high school graduate<br>◯ some college<br>◯ college graduate<br>◯ graduate school or more<br>◯ don't know
Person 5: Which of the following best fit this person's current work situation?	◯ works full time<br>◯ works part time<br>◯ is looking for work/employment<br>◯ retired<br>◯ homemaker<br>◯ student<br>◯ on maternity/paternity leave<br>◯ on illness/sick leave<br>◯ on disability<br>◯ other<br>◯ don't know
Person 5: Does this person currently consider themselves self-employed (including as an independent contractor or gig-economy worker)?	◯ yes<br>◯ no<br>◯ don't know

Person 5: Does this person currently work in any of the following high-risk settings for COVID-19 transmission?	☐ healthcare setting (hospital, clinic, urgent care, etc.)<br>☐ dense residential setting (nursing home, other long-term care facility)<br>☐ prison or jail<br>☐ meatpacking facility<br>☐ shipping or distribution facility<br>☐ high-volume retail facility (grocery store, etc.)<br>☐ don't know
Person 5: Does this person's employer offer them any of the following benefits at their current main job?	☐ paid sick leave<br>☐ paid vacation/personal leave<br>☐ health insurance<br>☐ disability insurance<br>☐ retirement plan<br>☐ other<br>☐ don't know<br>(Select all that apply.)
Person 5: On a scale of 0 (definitely not going to happen) to 10 (definitely going to happen), how likely is it that this person will lose their job because of the COVID-19 pandemic?	______________________
Person 5: On a scale of 0 (definitely not going to happen) to 10 (definitely going to happen), how likely is it that this person will receive fewer work hours at their job because of the COVID-19 pandemic?	______________________

	always (100%)	most of the time (75%)	half of the time (50%)	less than half of the time (25%)	never (0%)	don't know
Person 5: How often is this person required to work from outside of the home currently?	○	○	○	○	○	○
Person 5: How regularly is this person in close physical contact with co-workers during their work outside of the home currently?	○	○	○	○	○	○
Person 5: How regularly is this person in close physical contact with clients during their work outside of the home currently?	○	○	○	○	○	○

Person 5: Does this person plan to get a vaccine for COVID-19 when one becomes available?	○ yes<br>○ no<br>○ don't know
Person 5: Has this person had any symptoms (cough, fever, difficulty breathing, fatigue, body aches, diarrhea, runny nose, loss of smell or taste) consistent with COVID-19 in the last two weeks?	○ yes<br>○ no<br>○ don't know
Person 5: When did this person's symptoms begin?	______________________

Person 5: Is this person worried that they may have had COVID-19 because of their symptoms?	◯ yes<br>◯ no<br>◯ don't know
Person 5: Did this person experience any bias or discrimination because of their symptoms?	◯ yes<br>◯ no<br>◯ don't know
Person 5: What did this person do in response to their symptoms?	☐ nothing<br>☐ took over the counter medication (ibuprofen, acetaminophen, etc.)<br>☐ communicated with a health care provider over the phone<br>☐ visited a health care provider's office<br>☐ visited a retail clinic or pharmacy<br>☐ visited urgent care (FASTMed, etc.)<br>☐ visited the emergency room<br>☐ was admitted to the hospital<br>☐ other<br>☐ don't know<br>(Select all that apply.)
Person 5: Please specify what other action this person took in response to their symptoms.	________________________
Person 5: Did a health care provider tell this person that they may have COVID-19?	◯ yes<br>◯ no<br>◯ don't know
Person 5: If this person received a COVID-19 test due to their symptoms, what was the result?	◯ pending<br>◯ positive<br>◯ negative<br>◯ inconclusive<br>◯ did not receive a test<br>◯ don't know
Person 5: How many days was this person admitted to the hospital?	________________________
Person 5: Did this person receive any of the following interventions during their hospital admission?	☐ extra oxygen in your nose<br>☐ treatment in the intensive care unit (ICU)<br>☐ mechanical ventilation (intubation or a breathing tube)<br>☐ don't know
Person 5: Has this person returned to their normal health at this time?	◯ yes<br>◯ no<br>◯ don't know
Person 5: Which of the following did this person do to protect their friends and family after their symptoms began?	☐ wore a mask more frequently<br>☐ washed your hands with soap and water more frequently<br>☐ used hand sanitizer more frequently<br>☐ isolated yourself in your home more frequently<br>☐ stayed home more frequently<br>☐ wore disposable gloves more frequently<br>☐ don't know

For each additional person in the your household, please provide the following information.

Person 6: What is your relationship to this person?	◯ partner or spouse<br>◯ child<br>◯ parent<br>◯ sibling<br>◯ other family member<br>◯ in-home childcare provider or other caregiver<br>◯ other
Person 6: Please specify your relationship with this person.	__________________________
Person 6: What is this person's age?	__________________________<br>(Please specify their age in years)
Person 6: What is this person's sex?	◯ Female<br>◯ Male<br>◯ Other
Person 6: What is this person's race?	☐ American Indian or Alaska Native<br>☐ Asian<br>☐ Black or African American<br>☐ Native Hawaiian or Pacific Islander<br>☐ White<br>☐ Other<br>☐ don't know<br>(Select all that apply.)
Person 6: What is this person's ethnicity?	◯ Hispanic or Latino<br>◯ Not Hispanic or Latino<br>◯ Other<br>◯ don't know
Person 6: What is the highest level of education or schooling this person has completed?	◯ never attended school<br>◯ kindergarten - 8th grade<br>◯ some high school<br>◯ high school equivalency (GED)<br>◯ high school graduate<br>◯ some college<br>◯ college graduate<br>◯ graduate school or more<br>◯ don't know
Person 6: Which of the following best fit this person's current work situation?	◯ works full time<br>◯ works part time<br>◯ is looking for work/employment<br>◯ retired<br>◯ homemaker<br>◯ student<br>◯ on maternity/paternity leave<br>◯ on illness/sick leave<br>◯ on disability<br>◯ other<br>◯ don't know
Person 6: Does this person currently consider themselves self-employed (including as an independent contractor or gig-economy worker)?	◯ yes<br>◯ no<br>◯ don't know

Person 6: Does this person currently work in any of the following high-risk settings for COVID-19 transmission?

- ☐ healthcare setting (hospital, clinic, urgent care, etc.)
- ☐ dense residential setting (nursing home, other long-term care facility)
- ☐ prison or jail
- ☐ meatpacking facility
- ☐ shipping or distribution facility
- ☐ high-volume retail facility (grocery store, etc.)
- ☐ don't know

Person 6: Does this person's employer offer them any of the following benefits at their current main job?

- ☐ paid sick leave
- ☐ paid vacation/personal leave
- ☐ health insurance
- ☐ disability insurance
- ☐ retirement plan
- ☐ other
- ☐ don't know

(Select all that apply.)

Person 6: On a scale of 0 (definitely not going to happen) to 10 (definitely going to happen), how likely is it that this person will lose their job because of the COVID-19 pandemic?

____

Person 6: On a scale of 0 (definitely not going to happen) to 10 (definitely going to happen), how likely is it that this person will receive fewer work hours at their job because of the COVID-19 pandemic?

____

---

	always (100%)	most of the time (75%)	half of the time (50%)	less than half of the time (25%)	never (0%)	don't know
Person 6: How often is this person required to work from outside of the home currently?	◯	◯	◯	◯	◯	◯
Person 6: How regularly is this person in close physical contact with co-workers during their work outside of the home currently?	◯	◯	◯	◯	◯	◯
Person 6: How regularly is this person in close physical contact with clients during their work outside of the home currently?	◯	◯	◯	◯	◯	◯

Person 6: Does this person plan to get a vaccine for COVID-19 when one becomes available?

- ◯ yes
- ◯ no
- ◯ don't know

Person 6: Has this person had any symptoms (cough, fever, difficulty breathing, fatigue, body aches, diarrhea, runny nose, loss of smell or taste) consistent with COVID-19 in the last two weeks?

- ◯ yes
- ◯ no
- ◯ don't know

Person 6: When did this person's symptoms begin?

____

Person 6: Is this person worried that they may have had COVID-19 because of their symptoms?	◯ yes<br>◯ no<br>◯ don't know
Person 6: Did this person experience any bias or discrimination because of their symptoms?	◯ yes<br>◯ no<br>◯ don't know
Person 6: What did this person do in response to their symptoms?	☐ nothing<br>☐ took over the counter medication (ibuprofen, acetaminophen, etc.)<br>☐ communicated with a health care provider over the phone<br>☐ visited a health care provider's office<br>☐ visited a retail clinic or pharmacy<br>☐ visited urgent care (FASTMed, etc.)<br>☐ visited the emergency room<br>☐ was admitted to the hospital<br>☐ other<br>☐ don't know<br>(Select all that apply.)
Person 6: Please specify what other action this person took in response to their symptoms.	______________________________
Person 6: Did a health care provider tell this person that they may have COVID-19?	◯ yes<br>◯ no<br>◯ don't know
Person 6: If this person received a COVID-19 test due to their symptoms, what was the result?	◯ pending<br>◯ positive<br>◯ negative<br>◯ inconclusive<br>◯ did not receive a test<br>◯ don't know
Person 6: How many days was this person admitted to the hospital?	______________________________
Person 6: Did this person receive any of the following interventions during their hospital admission?	☐ extra oxygen in your nose<br>☐ treatment in the intensive care unit (ICU)<br>☐ mechanical ventilation (intubation or a breathing tube)<br>☐ don't know
Person 6: Has this person returned to their normal health at this time?	◯ yes<br>◯ no<br>◯ don't know
Person 6: Which of the following did this person do to protect their friends and family after their symptoms began?	☐ wore a mask more frequently<br>☐ washed your hands with soap and water more frequently<br>☐ used hand sanitizer more frequently<br>☐ isolated yourself in your home more frequently<br>☐ stayed home more frequently<br>☐ wore disposable gloves more frequently<br>☐ don't know

For each additional person in the your household, please provide the following information.

Person 7: What is your relationship to this person?

◯ partner or spouse
◯ child
◯ parent
◯ sibling
◯ other family member
◯ in-home childcare provider or other caregiver
◯ other

Person 7: Please specify your relationship with this person.

____

Person 7: What is this person's age?

____
(Please specify their age in years)

Person 7: What is this person's sex?

◯ Female
◯ Male
◯ Other

Person 7: What is this person's race?

☐ American Indian or Alaska Native
☐ Asian
☐ Black or African American
☐ Native Hawaiian or Pacific Islander
☐ White
☐ Other
☐ don't know
(Select all that apply.)

Person 7: What is this person's ethnicity?

◯ Hispanic or Latino
◯ Not Hispanic or Latino
◯ Other
◯ don't know

Person 7: What is the highest level of education or schooling this person has completed?

◯ never attended school
◯ kindergarten - 8th grade
◯ some high school
◯ high school equivalency (GED)
◯ high school graduate
◯ some college
◯ college graduate
◯ graduate school or more
◯ don't know

Person 7: Which of the following best fit this person's current work situation?

◯ works full time
◯ works part time
◯ is looking for work/employment
◯ retired
◯ homemaker
◯ student
◯ on maternity/paternity leave
◯ on illness/sick leave
◯ on disability
◯ other
◯ don't know

Person 7: Does this person currently consider themselves self-employed (including as an independent contractor or gig-economy worker)?

◯ yes
◯ no
◯ don't know

Person 7: Does this person currently work in any of the following high-risk settings for COVID-19 transmission?	☐ healthcare setting (hospital, clinic, urgent care, etc.)<br>☐ dense residential setting (nursing home, other long-term care facility)<br>☐ prison or jail<br>☐ meatpacking facility<br>☐ shipping or distribution facility<br>☐ high-volume retail facility (grocery store, etc.)<br>☐ don't know
Person 7: Does this person's employer offer them any of the following benefits at their current main job?	☐ paid sick leave<br>☐ paid vacation/personal leave<br>☐ health insurance<br>☐ disability insurance<br>☐ retirement plan<br>☐ other<br>☐ don't know<br>(Select all that apply.)
Person 7: On a scale of 0 (definitely not going to happen) to 10 (definitely going to happen), how likely is it that this person will lose their job because of the COVID-19 pandemic?	______________________
Person 7: On a scale of 0 (definitely not going to happen) to 10 (definitely going to happen), how likely is it that this person will receive fewer work hours at their job because of the COVID-19 pandemic?	______________________

	always (100%)	most of the time (75%)	half of the time (50%)	less than half of the time (25%)	never (0%)	don't know
Person 7: How often is this person required to work from outside of the home currently?	◯	◯	◯	◯	◯	◯
Person 7: How regularly is this person in close physical contact with co-workers during their work outside of the home currently?	◯	◯	◯	◯	◯	◯
Person 7: How regularly is this person in close physical contact with clients during their work outside of the home currently?	◯	◯	◯	◯	◯	◯

Person 7: Does this person plan to get a vaccine for COVID-19 when one becomes available?	◯ yes<br>◯ no<br>◯ don't know
Person 7: Has this person had any symptoms (cough, fever, difficulty breathing, fatigue, body aches, diarrhea, runny nose, loss of smell or taste) consistent with COVID-19 in the last two weeks?	◯ yes<br>◯ no<br>◯ don't know
Person 7: When did this person's symptoms begin?	______________________

Person 7: Is this person worried that they may have had COVID-19 because of their symptoms?	◯ yes<br>◯ no<br>◯ don't know
Person 7: Did this person experience any bias or discrimination because of their symptoms?	◯ yes<br>◯ no<br>◯ don't know
Person 7: What did this person do in response to their symptoms?	☐ nothing<br>☐ took over the counter medication (ibuprofen, acetaminophen, etc.)<br>☐ communicated with a health care provider over the phone<br>☐ visited a health care provider's office<br>☐ visited a retail clinic or pharmacy<br>☐ visited urgent care (FASTMed, etc.)<br>☐ visited the emergency room<br>☐ was admitted to the hospital<br>☐ other<br>☐ don't know<br>(Select all that apply.)
Person 7: Please specify what other action this person took in response to their symptoms.	__________________________
Person 7: Did a health care provider tell this person that they may have COVID-19?	◯ yes<br>◯ no<br>◯ don't know
Person 7: If this person received a COVID-19 test due to their symptoms, what was the result?	◯ pending<br>◯ positive<br>◯ negative<br>◯ inconclusive<br>◯ did not receive a test<br>◯ don't know
Person 7: How many days was this person admitted to the hospital?	__________________________
Person 7: Did this person receive any of the following interventions during their hospital admission?	☐ extra oxygen in your nose<br>☐ treatment in the intensive care unit (ICU)<br>☐ mechanical ventilation (intubation or a breathing tube)<br>☐ don't know
Person 7: Has this person returned to their normal health at this time?	◯ yes<br>◯ no<br>◯ don't know
Person 7: Which of the following did this person do to protect their friends and family after their symptoms began?	☐ wore a mask more frequently<br>☐ washed your hands with soap and water more frequently<br>☐ used hand sanitizer more frequently<br>☐ isolated yourself in your home more frequently<br>☐ stayed home more frequently<br>☐ wore disposable gloves more frequently<br>☐ don't know

For each additional person in the your household, please provide the following information.

Question	Response
Person 8: What is your relationship to this person?	◯ partner or spouse<br>◯ child<br>◯ parent<br>◯ sibling<br>◯ other family member<br>◯ in-home childcare provider or other caregiver<br>◯ other
Person 8: Please specify your relationship with this person.	__________________________
Person 8: What is this person's age?	__________________________<br>(Please specify their age in years)
Person 8: What is this person's sex?	◯ Female<br>◯ Male<br>◯ Other
Person 8: What is this person's race?	☐ American Indian or Alaska Native<br>☐ Asian<br>☐ Black or African American<br>☐ Native Hawaiian or Pacific Islander<br>☐ White<br>☐ Other<br>☐ don't know<br>(Select all that apply.)
Person 8: What is this person's ethnicity?	◯ Hispanic or Latino<br>◯ Not Hispanic or Latino<br>◯ Other<br>◯ don't know
Person 8: What is the highest level of education or schooling this person has completed?	◯ never attended school<br>◯ kindergarten - 8th grade<br>◯ some high school<br>◯ high school equivalency (GED)<br>◯ high school graduate<br>◯ some college<br>◯ college graduate<br>◯ graduate school or more<br>◯ don't know
Person 8: Which of the following best fit this person's current work situation?	◯ works full time<br>◯ works part time<br>◯ is looking for work/employment<br>◯ retired<br>◯ homemaker<br>◯ student<br>◯ on maternity/paternity leave<br>◯ on illness/sick leave<br>◯ on disability<br>◯ other<br>◯ don't know
Person 8: Does this person currently consider themselves self-employed (including as an independent contractor or gig-economy worker)?	◯ yes<br>◯ no<br>◯ don't know

Person 8: Does this person currently work in any of the following high-risk settings for COVID-19 transmission?

- ☐ healthcare setting (hospital, clinic, urgent care, etc.)
- ☐ dense residential setting (nursing home, other long-term care facility)
- ☐ prison or jail
- ☐ meatpacking facility
- ☐ shipping or distribution facility
- ☐ high-volume retail facility (grocery store, etc.)
- ☐ don't know

Person 8: Does this person's employer offer them any of the following benefits at their current main job?

- ☐ paid sick leave
- ☐ paid vacation/personal leave
- ☐ health insurance
- ☐ disability insurance
- ☐ retirement plan
- ☐ other
- ☐ don't know

(Select all that apply.)

Person 8: On a scale of 0 (definitely not going to happen) to 10 (definitely going to happen), how likely is it that this person will lose their job because of the COVID-19 pandemic?

____

Person 8: On a scale of 0 (definitely not going to happen) to 10 (definitely going to happen), how likely is it that this person will receive fewer work hours at their job because of the COVID-19 pandemic?

____

	always (100%)	most of the time (75%)	half of the time (50%)	less than half of the time (25%)	never (0%)	don't know
Person 8: How often is this person required to work from outside of the home currently?	○	○	○	○	○	○
Person 8: How regularly is this person in close physical contact with co-workers during their work outside of the home currently?	○	○	○	○	○	○
Person 8: How regularly is this person in close physical contact with clients during their work outside of the home currently?	○	○	○	○	○	○

Person 8: Does this person plan to get a vaccine for COVID-19 when one becomes available?

- ○ yes
- ○ no
- ○ don't know

Person 8: Has this person had any symptoms (cough, fever, difficulty breathing, fatigue, body aches, diarrhea, runny nose, loss of smell or taste) consistent with COVID-19 in the last two weeks?

- ○ yes
- ○ no
- ○ don't know

Person 8: When did this person's symptoms begin?

____

Person 8: Is this person worried that they may have had COVID-19 because of their symptoms?	◯ yes<br>◯ no<br>◯ don't know
Person 8: Did this person experience any bias or discrimination because of their symptoms?	◯ yes<br>◯ no<br>◯ don't know
Person 8: What did this person do in response to their symptoms?	☐ nothing<br>☐ took over the counter medication (ibuprofen, acetaminophen, etc.)<br>☐ communicated with a health care provider over the phone<br>☐ visited a health care provider's office<br>☐ visited a retail clinic or pharmacy<br>☐ visited urgent care (FASTMed, etc.)<br>☐ visited the emergency room<br>☐ was admitted to the hospital<br>☐ other<br>☐ don't know<br>(Select all that apply.)
Person 8: Please specify what other action this person took in response to their symptoms.	__________________________________
Person 8: Did a health care provider tell this person that they may have COVID-19?	◯ yes<br>◯ no<br>◯ don't know
Person 8: If this person received a COVID-19 test due to their symptoms, what was the result?	◯ pending<br>◯ positive<br>◯ negative<br>◯ inconclusive<br>◯ did not receive a test<br>◯ don't know
Person 8: How many days was this person admitted to the hospital?	__________________________________
Person 8: Did this person receive any of the following interventions during their hospital admission?	☐ extra oxygen in your nose<br>☐ treatment in the intensive care unit (ICU)<br>☐ mechanical ventilation (intubation or a breathing tube)<br>☐ don't know
Person 8: Has this person returned to their normal health at this time?	◯ yes<br>◯ no<br>◯ don't know
Person 8: Which of the following did this person do to protect their friends and family after their symptoms began?	☐ wore a mask more frequently<br>☐ washed your hands with soap and water more frequently<br>☐ used hand sanitizer more frequently<br>☐ isolated yourself in your home more frequently<br>☐ stayed home more frequently<br>☐ wore disposable gloves more frequently<br>☐ don't know

For each additional person in the your household, please provide the following information.

Person 9: What is your relationship to this person?	◯ partner or spouse<br>◯ child<br>◯ parent<br>◯ sibling<br>◯ other family member<br>◯ in-home childcare provider or other caregiver<br>◯ other
Person 9: Please specify your relationship with this person.	__________________________________
Person 9: What is this person's age?	__________________________________<br>(Please specify their age in years)
Person 9: What is this person's sex?	◯ Female<br>◯ Male<br>◯ Other
Person 9: What is this person's race?	☐ American Indian or Alaska Native<br>☐ Asian<br>☐ Black or African American<br>☐ Native Hawaiian or Pacific Islander<br>☐ White<br>☐ Other<br>☐ don't know<br>(Select all that apply.)
Person 9: What is this person's ethnicity?	◯ Hispanic or Latino<br>◯ Not Hispanic or Latino<br>◯ Other<br>◯ don't know
Person 9: What is the highest level of education or schooling this person has completed?	◯ never attended school<br>◯ kindergarten - 8th grade<br>◯ some high school<br>◯ high school equivalency (GED)<br>◯ high school graduate<br>◯ some college<br>◯ college graduate<br>◯ graduate school or more<br>◯ don't know
Person 9: Which of the following best fit this person's current work situation?	◯ works full time<br>◯ works part time<br>◯ is looking for work/employment<br>◯ retired<br>◯ homemaker<br>◯ student<br>◯ on maternity/paternity leave<br>◯ on illness/sick leave<br>◯ on disability<br>◯ other<br>◯ don't know
Person 9: Does this person currently consider themselves self-employed (including as an independent contractor or gig-economy worker)?	◯ yes<br>◯ no<br>◯ don't know

Person 9: Does this person currently work in any of the following high-risk settings for COVID-19 transmission?

☐ healthcare setting (hospital, clinic, urgent care, etc.)
☐ dense residential setting (nursing home, other long-term care facility)
☐ prison or jail
☐ meatpacking facility
☐ shipping or distribution facility
☐ high-volume retail facility (grocery store, etc.)
☐ don't know

Person 9: Does this person's employer offer them any of the following benefits at their current main job?

☐ paid sick leave
☐ paid vacation/personal leave
☐ health insurance
☐ disability insurance
☐ retirement plan
☐ other
☐ don't know
(Select all that apply.)

Person 9: On a scale of 0 (definitely not going to happen) to 10 (definitely going to happen), how likely is it that this person will lose their job because of the COVID-19 pandemic?

____

Person 9: On a scale of 0 (definitely not going to happen) to 10 (definitely going to happen), how likely is it that this person will receive fewer work hours at their job because of the COVID-19 pandemic?

____

	always (100%)	most of the time (75%)	half of the time (50%)	less than half of the time (25%)	never (0%)	don't know
Person 9: How often is this person required to work from outside of the home currently?	◯	◯	◯	◯	◯	◯
Person 9: How regularly is this person in close physical contact with co-workers during their work outside of the home currently?	◯	◯	◯	◯	◯	◯
Person 9: How regularly is this person in close physical contact with clients during their work outside of the home currently?	◯	◯	◯	◯	◯	◯

Person 9: Does this person plan to get a vaccine for COVID-19 when one becomes available?

◯ yes
◯ no
◯ don't know

Person 9: Has this person had any symptoms (cough, fever, difficulty breathing, fatigue, body aches, diarrhea, runny nose, loss of smell or taste) consistent with COVID-19 in the last two weeks?

◯ yes
◯ no
◯ don't know

Person 9: When did this person's symptoms begin?

____

Question	Response
Person 9: Is this person worried that they may have had COVID-19 because of their symptoms?	◯ yes<br>◯ no<br>◯ don't know
Person 9: Did this person experience any bias or discrimination because of their symptoms?	◯ yes<br>◯ no<br>◯ don't know
Person 9: What did this person do in response to their symptoms?	☐ nothing<br>☐ took over the counter medication (ibuprofen, acetaminophen, etc.)<br>☐ communicated with a health care provider over the phone<br>☐ visited a health care provider's office<br>☐ visited a retail clinic or pharmacy<br>☐ visited urgent care (FASTMed, etc.)<br>☐ visited the emergency room<br>☐ was admitted to the hospital<br>☐ other<br>☐ don't know<br>(Select all that apply.)
Person 9: Please specify what other action this person took in response to their symptoms.	__________________________________
Person 9: Did a health care provider tell this person that they may have COVID-19?	◯ yes<br>◯ no<br>◯ don't know
Person 9: If this person received a COVID-19 test due to their symptoms, what was the result?	◯ pending<br>◯ positive<br>◯ negative<br>◯ inconclusive<br>◯ did not receive a test<br>◯ don't know
Person 9: How many days was this person admitted to the hospital?	__________________________________
Person 9: Did this person receive any of the following interventions during their hospital admission?	☐ extra oxygen in your nose<br>☐ treatment in the intensive care unit (ICU)<br>☐ mechanical ventilation (intubation or a breathing tube)<br>☐ don't know
Person 9: Has this person returned to their normal health at this time?	◯ yes<br>◯ no<br>◯ don't know
Person 9: Which of the following did this person do to protect their friends and family after their symptoms began?	☐ wore a mask more frequently<br>☐ washed your hands with soap and water more frequently<br>☐ used hand sanitizer more frequently<br>☐ isolated yourself in your home more frequently<br>☐ stayed home more frequently<br>☐ wore disposable gloves more frequently<br>☐ don't know

For each additional person in the your household, please provide the following information.

Person 10: What is your relationship to this person?	◯ partner or spouse<br>◯ child<br>◯ parent<br>◯ sibling<br>◯ other family member<br>◯ in-home childcare provider or other caregiver<br>◯ other
Person 10: Please specify your relationship with this person.	_________________________________
Person 10: What is this person's age?	_________________________________<br>(Please specify their age in years)
Person 10: What is this person's sex?	◯ Female<br>◯ Male<br>◯ Other
Person 10: What is this person's race?	☐ American Indian or Alaska Native<br>☐ Asian<br>☐ Black or African American<br>☐ Native Hawaiian or Pacific Islander<br>☐ White<br>☐ Other<br>☐ don't know<br>(Select all that apply.)
Person 10: What is this person's ethnicity?	◯ Hispanic or Latino<br>◯ Not Hispanic or Latino<br>◯ Other<br>◯ don't know
Person 10: What is the highest level of education or schooling this person has completed?	◯ never attended school<br>◯ kindergarten - 8th grade<br>◯ some high school<br>◯ high school equivalency (GED)<br>◯ high school graduate<br>◯ some college<br>◯ college graduate<br>◯ graduate school or more<br>◯ don't know
Person 10: Which of the following best fit this person's current work situation?	◯ works full time<br>◯ works part time<br>◯ is looking for work/employment<br>◯ retired<br>◯ homemaker<br>◯ student<br>◯ on maternity/paternity leave<br>◯ on illness/sick leave<br>◯ on disability<br>◯ other<br>◯ don't know
Person 10: Does this person currently consider themselves self-employed (including as an independent contractor or gig-economy worker)?	◯ yes<br>◯ no<br>◯ don't know

Person 10: Does this person currently work in any of the following high-risk settings for COVID-19 transmission?	☐ healthcare setting (hospital, clinic, urgent care, etc.)<br>☐ dense residential setting (nursing home, other long-term care facility)<br>☐ prison or jail<br>☐ meatpacking facility<br>☐ shipping or distribution facility<br>☐ high-volume retail facility (grocery store, etc.)<br>☐ don't know
Person 10: Does this person's employer offer them any of the following benefits at their current main job?	☐ paid sick leave<br>☐ paid vacation/personal leave<br>☐ health insurance<br>☐ disability insurance<br>☐ retirement plan<br>☐ other<br>☐ don't know<br>(Select all that apply.)
Person 10: On a scale of 0 (definitely not going to happen) to 10 (definitely going to happen), how likely is it that this person will lose their job because of the COVID-19 pandemic?	______________________
Person 10: On a scale of 0 (definitely not going to happen) to 10 (definitely going to happen), how likely is it that this person will receive fewer work hours at their job because of the COVID-19 pandemic?	______________________

	always (100%)	most of the time (75%)	half of the time (50%)	less than half of the time (25%)	never (0%)	don't know
Person 10: How often is this person required to work from outside of the home currently?	○	○	○	○	○	○
Person 10: How regularly is this person in close physical contact with co-workers during their work outside of the home currently?	○	○	○	○	○	○
Person 10: How regularly is this person in close physical contact with clients during their work outside of the home currently?	○	○	○	○	○	○

Person 10: Does this person plan to get a vaccine for COVID-19 when one becomes available?	○ yes<br>○ no<br>○ don't know
Person 10: Has this person had any symptoms (cough, fever, difficulty breathing, fatigue, body aches, diarrhea, runny nose, loss of smell or taste) consistent with COVID-19 in the last two weeks?	○ yes<br>○ no<br>○ don't know
Person 10: When did this person's symptoms begin?	______________________

Person 10: Is this person worried that they may have had COVID-19 because of their symptoms?	◯ yes<br>◯ no<br>◯ don't know
Person 10: Did this person experience any bias or discrimination because of their symptoms?	◯ yes<br>◯ no<br>◯ don't know
Person 10: What did this person do in response to their symptoms?	☐ nothing<br>☐ took over the counter medication (ibuprofen, acetaminophen, etc.)<br>☐ communicated with a health care provider over the phone<br>☐ visited a health care provider's office<br>☐ visited a retail clinic or pharmacy<br>☐ visited urgent care (FASTMed, etc.)<br>☐ visited the emergency room<br>☐ was admitted to the hospital<br>☐ other<br>☐ don't know<br>(Select all that apply.)
Person 10: Please specify what other action this person took in response to their symptoms.	__________________________________
Person 10: Did a health care provider tell this person that they may have COVID-19?	◯ yes<br>◯ no<br>◯ don't know
Person 10: If this person received a COVID-19 test due to their symptoms, what was the result?	◯ pending<br>◯ positive<br>◯ negative<br>◯ inconclusive<br>◯ did not receive a test<br>◯ don't know
Person 10: How many days was this person admitted to the hospital?	__________________________________
Person 10: Did this person receive any of the following interventions during their hospital admission?	☐ extra oxygen in your nose<br>☐ treatment in the intensive care unit (ICU)<br>☐ mechanical ventilation (intubation or a breathing tube)<br>☐ don't know
Person 10: Has this person returned to their normal health at this time?	◯ yes<br>◯ no<br>◯ don't know
Person 10: Which of the following did this person do to protect their friends and family after their symptoms began?	☐ wore a mask more frequently<br>☐ washed your hands with soap and water more frequently<br>☐ used hand sanitizer more frequently<br>☐ isolated yourself in your home more frequently<br>☐ stayed home more frequently<br>☐ wore disposable gloves more frequently<br>☐ don't know

For each additional person in the your household, please provide the following information.

Person 11: What is your relationship to this person?	◯ partner or spouse<br>◯ child<br>◯ parent<br>◯ sibling<br>◯ other family member<br>◯ in-home childcare provider or other caregiver<br>◯ other
Person 11: Please specify your relationship with this person.	__________________________
Person 11: What is this person's age?	__________________________<br>(Please specify their age in years)
Person 11: What is this person's sex?	◯ Female<br>◯ Male<br>◯ Other
Person 11: What is this person's race?	☐ American Indian or Alaska Native<br>☐ Asian<br>☐ Black or African American<br>☐ Native Hawaiian or Pacific Islander<br>☐ White<br>☐ Other<br>☐ don't know<br>(Select all that apply.)
Person 11: What is this person's ethnicity?	◯ Hispanic or Latino<br>◯ Not Hispanic or Latino<br>◯ Other<br>◯ don't know
Person 11: What is the highest level of education or schooling this person has completed?	◯ never attended school<br>◯ kindergarten - 8th grade<br>◯ some high school<br>◯ high school equivalency (GED)<br>◯ high school graduate<br>◯ some college<br>◯ college graduate<br>◯ graduate school or more<br>◯ don't know
Person 11: Which of the following best fit this person's current work situation?	◯ works full time<br>◯ works part time<br>◯ is looking for work/employment<br>◯ retired<br>◯ homemaker<br>◯ student<br>◯ on maternity/paternity leave<br>◯ on illness/sick leave<br>◯ on disability<br>◯ other<br>◯ don't know
Person 11: Does this person currently consider themselves self-employed (including as an independent contractor or gig-economy worker)?	◯ yes<br>◯ no<br>◯ don't know

Person 11: Does this person currently work in any of the following high-risk settings for COVID-19 transmission?	☐ healthcare setting (hospital, clinic, urgent care, etc.)<br>☐ dense residential setting (nursing home, other long-term care facility)<br>☐ prison or jail<br>☐ meatpacking facility<br>☐ shipping or distribution facility<br>☐ high-volume retail facility (grocery store, etc.)<br>☐ don't know
Person 11: Does this person's employer offer them any of the following benefits at their current main job?	☐ paid sick leave<br>☐ paid vacation/personal leave<br>☐ health insurance<br>☐ disability insurance<br>☐ retirement plan<br>☐ other<br>☐ don't know<br>(Select all that apply.)
Person 11: On a scale of 0 (definitely not going to happen) to 10 (definitely going to happen), how likely is it that this person will lose their job because of the COVID-19 pandemic?	______________________
Person 11: On a scale of 0 (definitely not going to happen) to 10 (definitely going to happen), how likely is it that this person will receive fewer work hours at their job because of the COVID-19 pandemic?	______________________

	always (100%)	most of the time (75%)	half of the time (50%)	less than half of the time (25%)	never (0%)	don't know
Person 11: How often is this person required to work from outside of the home currently?	○	○	○	○	○	○
Person 11: How regularly is this person in close physical contact with co-workers during their work outside of the home currently?	○	○	○	○	○	○
Person 11: How regularly is this person in close physical contact with clients during their work outside of the home currently?	○	○	○	○	○	○

Person 11: Does this person plan to get a vaccine for COVID-19 when one becomes available?	○ yes<br>○ no<br>○ don't know
Person 11: Has this person had any symptoms (cough, fever, difficulty breathing, fatigue, body aches, diarrhea, runny nose, loss of smell or taste) consistent with COVID-19 in the last two weeks?	○ yes<br>○ no<br>○ don't know
Person 11: When did this person's symptoms begin?	______________________

Person 11: Is this person worried that they may have had COVID-19 because of their symptoms?	◯ yes<br>◯ no<br>◯ don't know
Person 11: Did this person experience any bias or discrimination because of their symptoms?	◯ yes<br>◯ no<br>◯ don't know
Person 11: What did this person do in response to their symptoms?	☐ nothing<br>☐ took over the counter medication (ibuprofen, acetaminophen, etc.)<br>☐ communicated with a health care provider over the phone<br>☐ visited a health care provider's office<br>☐ visited a retail clinic or pharmacy<br>☐ visited urgent care (FASTMed, etc.)<br>☐ visited the emergency room<br>☐ was admitted to the hospital<br>☐ other<br>☐ don't know<br>(Select all that apply.)
Person 11: Please specify what other action this person took in response to their symptoms.	__________________________________
Person 11: Did a health care provider tell this person that they may have COVID-19?	◯ yes<br>◯ no<br>◯ don't know
Person 11: If this person received a COVID-19 test due to their symptoms, what was the result?	◯ pending<br>◯ positive<br>◯ negative<br>◯ inconclusive<br>◯ did not receive a test<br>◯ don't know
Person 11: How many days was this person admitted to the hospital?	__________________________________
Person 11: Did this person receive any of the following interventions during their hospital admission?	☐ extra oxygen in your nose<br>☐ treatment in the intensive care unit (ICU)<br>☐ mechanical ventilation (intubation or a breathing tube)<br>☐ don't know
Person 11: Has this person returned to their normal health at this time?	◯ yes<br>◯ no<br>◯ don't know
Person 11: Which of the following did this person do to protect their friends and family after their symptoms began?	☐ wore a mask more frequently<br>☐ washed your hands with soap and water more frequently<br>☐ used hand sanitizer more frequently<br>☐ isolated yourself in your home more frequently<br>☐ stayed home more frequently<br>☐ wore disposable gloves more frequently<br>☐ don't know

For each additional person in the your household, please provide the following information.

Person 12: What is your relationship to this person?	◯ partner or spouse<br>◯ child<br>◯ parent<br>◯ sibling<br>◯ other family member<br>◯ in-home childcare provider or other caregiver<br>◯ other
Person 12: Please specify your relationship with this person.	________________________________
Person 12: What is this person's age?	________________________________<br>(Please specify their age in years)
Person 12: What is this person's sex?	◯ Female<br>◯ Male<br>◯ Other
Person 12: What is this person's race?	☐ American Indian or Alaska Native<br>☐ Asian<br>☐ Black or African American<br>☐ Native Hawaiian or Pacific Islander<br>☐ White<br>☐ Other<br>☐ don't know<br>(Select all that apply.)
Person 12: What is this person's ethnicity?	◯ Hispanic or Latino<br>◯ Not Hispanic or Latino<br>◯ Other<br>◯ don't know
Person 12: What is the highest level of education or schooling this person has completed?	◯ never attended school<br>◯ kindergarten - 8th grade<br>◯ some high school<br>◯ high school equivalency (GED)<br>◯ high school graduate<br>◯ some college<br>◯ college graduate<br>◯ graduate school or more<br>◯ don't know
Person 12: Which of the following best fit this person's current work situation?	◯ works full time<br>◯ works part time<br>◯ is looking for work/employment<br>◯ retired<br>◯ homemaker<br>◯ student<br>◯ on maternity/paternity leave<br>◯ on illness/sick leave<br>◯ on disability<br>◯ other<br>◯ don't know
Person 12: Does this person currently consider themselves self-employed (including as an independent contractor or gig-economy worker)?	◯ yes<br>◯ no<br>◯ don't know

Person 12: Does this person currently work in any of the following high-risk settings for COVID-19 transmission?

- ☐ healthcare setting (hospital, clinic, urgent care, etc.)
- ☐ dense residential setting (nursing home, other long-term care facility)
- ☐ prison or jail
- ☐ meatpacking facility
- ☐ shipping or distribution facility
- ☐ high-volume retail facility (grocery store, etc.)
- ☐ don't know

Person 12: Does this person's employer offer them any of the following benefits at their current main job?

- ☐ paid sick leave
- ☐ paid vacation/personal leave
- ☐ health insurance
- ☐ disability insurance
- ☐ retirement plan
- ☐ other
- ☐ don't know

(Select all that apply.)

Person 12: On a scale of 0 (definitely not going to happen) to 10 (definitely going to happen), how likely is it that this person will lose their job because of the COVID-19 pandemic?

____

Person 12: On a scale of 0 (definitely not going to happen) to 10 (definitely going to happen), how likely is it that this person will receive fewer work hours at their job because of the COVID-19 pandemic?

____

---

	always (100%)	most of the time (75%)	half of the time (50%)	less than half of the time (25%)	never (0%)	don't know
Person 12: How often is this person required to work from outside of the home currently?	○	○	○	○	○	○
Person 12: How regularly is this person in close physical contact with co-workers during their work outside of the home currently?	○	○	○	○	○	○
Person 12: How regularly is this person in close physical contact with clients during their work outside of the home currently?	○	○	○	○	○	○

Person 12: Does this person plan to get a vaccine for COVID-19 when one becomes available?

- ○ yes
- ○ no
- ○ don't know

Person 12: Has this person had any symptoms (cough, fever, difficulty breathing, fatigue, body aches, diarrhea, runny nose, loss of smell or taste) consistent with COVID-19 in the last two weeks?

- ○ yes
- ○ no
- ○ don't know

Person 12: When did this person's symptoms begin?

____

Person 12: Is this person worried that they may have had COVID-19 because of their symptoms?	◯ yes<br>◯ no<br>◯ don't know
Person 12: Did this person experience any bias or discrimination because of their symptoms?	◯ yes<br>◯ no<br>◯ don't know
Person 12: What did this person do in response to their symptoms?	☐ nothing<br>☐ took over the counter medication (ibuprofen, acetaminophen, etc.)<br>☐ communicated with a health care provider over the phone<br>☐ visited a health care provider's office<br>☐ visited a retail clinic or pharmacy<br>☐ visited urgent care (FASTMed, etc.)<br>☐ visited the emergency room<br>☐ was admitted to the hospital<br>☐ other<br>☐ don't know<br>(Select all that apply.)
Person 12: Please specify what other action this person took in response to their symptoms.	__________________________________
Person 12: Did a health care provider tell this person that they may have COVID-19?	◯ yes<br>◯ no<br>◯ don't know
Person 12: If this person received a COVID-19 test due to their symptoms, what was the result?	◯ pending<br>◯ positive<br>◯ negative<br>◯ inconclusive<br>◯ did not receive a test<br>◯ don't know
Person 12: How many days was this person admitted to the hospital?	__________________________________
Person 12: Did this person receive any of the following interventions during their hospital admission?	☐ extra oxygen in your nose<br>☐ treatment in the intensive care unit (ICU)<br>☐ mechanical ventilation (intubation or a breathing tube)<br>☐ don't know
Person 12: Has this person returned to their normal health at this time?	◯ yes<br>◯ no<br>◯ don't know
Person 12: Which of the following did this person do to protect their friends and family after their symptoms began?	☐ wore a mask more frequently<br>☐ washed your hands with soap and water more frequently<br>☐ used hand sanitizer more frequently<br>☐ isolated yourself in your home more frequently<br>☐ stayed home more frequently<br>☐ wore disposable gloves more frequently<br>☐ don't know

Please provide the following information about your mental health and wellness.

How serious a problem would you say the COVID-19 pandemic is for you personally right now?
- ◯ very serious
- ◯ somewhat serious
- ◯ not too serious
- ◯ not at all serious

How serious a problem would you say the COVID-19 pandemic is for people in your community right now?
- ◯ very serious
- ◯ somewhat serious
- ◯ not too serious
- ◯ not at all serious

How serious a problem would you say the COVID-19 pandemic is for people in the United States right now?
- ◯ very serious
- ◯ somewhat serious
- ◯ not too serious
- ◯ not at all serious

How serious a problem would you say the COVID-19 pandemic is for people around the world right now?
- ◯ very serious
- ◯ somewhat serious
- ◯ not too serious
- ◯ not at all serious

In the last two weeks, how often have you encountered news media, a viral/trending event, online videos, online discussions, or photos in which someone like you was threatened or harmed?
- ◯ Never
- ◯ About once a month
- ◯ About once a week
- ◯ About once a day
- ◯ Several times a day

In the last two weeks, how often have you encountered news media, a viral/trending event, online videos, online discussions, or photos in which negative stereotypes of people like you were promoted as if they were true?
- ◯ Never
- ◯ About once a month
- ◯ About once a week
- ◯ About once a day
- ◯ Several times a day

The COVID-19 pandemic may cause challenges for some people regardless of whether they are infected. How concerned are you about each of the following things?

	not at all concerned	not too concerned	somewhat concerned	very concerned
Getting the healthcare you need (including care for mental health)	◯	◯	◯	◯
Having a place to live	◯	◯	◯	◯
Being able to interact with other people	◯	◯	◯	◯
Getting food, water, and other household supplies	◯	◯	◯	◯
Getting medication	◯	◯	◯	◯
Having transportation to get where you need to go	◯	◯	◯	◯
Caring for your family and friends	◯	◯	◯	◯

During the last two weeks, how often have you been bothered by the following problems?

	not at all	several days	more than half of the days	nearly every day
Feeling nervous, anxious, or on edge	◯	◯	◯	◯
Not being able to stop or control worrying	◯	◯	◯	◯
Worrying too much about different things	◯	◯	◯	◯
Trouble relaxing	◯	◯	◯	◯
Being so restless that it's hard to sit still	◯	◯	◯	◯
Becoming easily annoyed or irritable	◯	◯	◯	◯
Feeling afraid as if something awful might happen	◯	◯	◯	◯

How difficult have these problems made it for you to do your work, take care of things at home, or get along with other people?

◯ not difficult
◯ somewhat difficult
◯ very difficult
◯ extremely difficult

In the last two weeks, how often have you experienced the following feelings and behaviors?

	Rarely or none of the time (< 1 day)	Some or a little of the time (1-2 days)	Occasionally or a moderate amount of the time (3-4 days)	Most or all of the time (5-7 days)
I was bothered by things that usually don't bother me.	◯	◯	◯	◯
I did not feel like eating; my appetite was poor.	◯	◯	◯	◯
I felt that I could not shake off the blues even with the help from my family and friends.	◯	◯	◯	◯
I felt that I was not as good as other people.	◯	◯	◯	◯
I had trouble keeping my mind on what I was doing.	◯	◯	◯	◯
I felt depressed.	◯	◯	◯	◯
I felt that everything I did was an effort.	◯	◯	◯	◯
I felt hopeless about the future.	◯	◯	◯	◯
I thought my life had been a failure.	◯	◯	◯	◯

I felt fearful.	◯	◯	◯	◯
My sleep was restless.	◯	◯	◯	◯
I was unhappy.	◯	◯	◯	◯
I talked less than usual.	◯	◯	◯	◯
I felt lonely.	◯	◯	◯	◯
People were unfriendly.	◯	◯	◯	◯
I did not enjoy life.	◯	◯	◯	◯
I had crying spells.	◯	◯	◯	◯
I felt sad.	◯	◯	◯	◯
I felt that people disliked me.	◯	◯	◯	◯
I could not get "going".	◯	◯	◯	◯

How did you complete this survey?

- ◯ on a computer (laptop or desktop)
- ◯ on a mobile device (cell phone or tablet)
- ◯ on a phone with an interviewer
- ◯ other

¿Cuál de las siguientes es la mejor descripción de su situación laboral actual?

- ◯ Empleo a tiempo completo
- ◯ Empleo a tiempo parcial
- ◯ En busca de empleo
- ◯ Jubilado
- ◯ Ama de casa
- ◯ Estudiante
- ◯ Permiso de maternidad o paternidad
- ◯ Permiso por enfermedad
- ◯ Desempleado por discapacidad
- ◯ Otra

¿Se considera usted actualmente empleado por cuenta propia (contratista independiente, trabajador esporádico (gig), etc.)?

- ◯ Sí
- ◯ No
- ◯ No sabe

En una escala de 0 (definitivamente no va a suceder) a 10 (definitivamente va a suceder), ¿qué tan probable es que usted pierda su empleo debido a la pandemia de COVID-19?

____

En una escala de 0 (definitivamente no va a suceder) a 10 (definitivamente va a suceder), ¿qué tan probable es que le asignen menos horas de trabajo debido a la pandemia de COVID-19?

____

En su opinión ¿qué tan probable es que su grupo familiar se quede sin dinero en los próximos 3 meses? Dé su respuesta en una escala de 0 (definitivamente no va a suceder) a 10 (definitivamente va a suceder).

____

	Todo el tiempo (100%)	La mayor parte del tiempo (75%)	La mitad del tiempo (50%)	Menos de la mitad del tiempo (25%)	Nunca (0%)

Actualmente, ¿con qué frecuencia se le exige que trabaje fuera de su residencia?	○	○	○	○	○
Actualmente, ¿con qué frecuencia se encuentra usted físicamente cerca de sus compañeros de trabajo mientras trabaja fuera de su residencia?	○	○	○	○	○
Actualmente, ¿con qué frecuencia se encuentra usted físicamente cerca de los clientes mientras trabaja fuera de su residencia?	○	○	○	○	○
Actualmente, ¿con qué frecuencia tiene acceso a guantes desechables mientras trabaja fuera de su residencia?	○	○	○	○	○
Actualmente, ¿con qué frecuencia tiene acceso a una mascarilla mientras trabaja fuera de su residencia?	○	○	○	○	○
Actualmente, ¿con qué frecuencia lleva usted guantes desechables mientras trabaja fuera de su residencia?	○	○	○	○	○
Actualmente, ¿con qué frecuencia lleva usted mascarilla mientras trabaja fuera de su residencia?	○	○	○	○	○
Actualmente, ¿con qué frecuencia se lava las manos con agua y jabón mientras trabaja fuera de su residencia?	○	○	○	○	○
Actualmente, ¿con qué frecuencia se desinfecta las manos con desinfectante de manos mientras trabaja fuera de su residencia?	○	○	○	○	○
Actualmente, ¿cuánto le preocupa exponerse a la COVID-19 mientras trabaja fuera de su residencia?	○	○	○	○	○

¿Trabaja usted actualmente en alguno de los siguientes ambientes de alto riesgo de contagio de COVID-19?

- ☐ Ambiente de asistencia médica (hospital, clínica, centro de urgencias)
- ☐ Ambiente residencial denso (hogar de ancianos, otro centro de asistencia de larga duración)
- ☐ Prisión o cárcel
- ☐ Establecimiento de envasado de carne
- ☐ Establecimiento de envío o distribución
- ☐ Establecimiento minorista de alto volumen (tienda de provisiones, etc.)

¿Tiene usted seguro médico o de salud (seguro privado, seguro que usted ha comprado, Medicare, Medicaid o algún otro tipo de seguro)?

- ○ Sí
- ○ No
- ○ No sabe

¿Cuál es su principal seguro de salud?

- ○ Seguro médico privado por empleo o establecimiento educacional
- ○ Seguro comprado en un intercambio de seguros de salud estatal o federal, tal como healthcare.gov
- ○ Seguro comprado directamente a una compañía de seguros.
- ○ Medicare
- ○ Medi-Gap
- ○ Medicaid
- ○ Asistencia médica militar (TRICARE, VA, CHAMP-VA, etc.)
- ○ Servicio de Salud Indígena
- ○ Otro

((Seleccione uno (su seguro principal).))

Si marcó "Otro", especifique la fuente de seguro médico.

____

¿Está usted actualmente embarazada?

- ○ Sí
- ○ No

	Excelente	Muy buena	Buena	Regular	Mala
En general, ¿cómo calificaría usted su salud en las últimas dos semanas?	○	○	○	○	○

Con que frequencia ha hecho lo siguiente durante las ultimas dos semanas para protegerse contra la infeccion?

	Siempre (100%)	La mayor parte del tiempo (75%)	La mitad del tiempo (50%)	Menos de la mitad del tiempo (25%)	Nunca (0%)
Llevar mascarilla	○	○	○	○	○
Lavarse las manos y/o limpiárselas con desinfectante a menudo	○	○	○	○	○
Mantenerse al menos a 6 pies de distancia de los demás	○	○	○	○	○

Evitar grandes reuniones, espacios públicos o multitudes	◯	◯	◯	◯	◯
Evitar el contacto con personas de alto riesgo	◯	◯	◯	◯	◯
No comer en restaurante, ni siquiera comprar comida para llevar	◯	◯	◯	◯	◯
Trabajar o estudiar en casa en lugar de ir a una oficina o a un aula	◯	◯	◯	◯	◯
No dar la mano ni tocar a otras personas.	◯	◯	◯	◯	◯
Quedarse en casa cuando se está enfermo	◯	◯	◯	◯	◯
Limpiar las superficies con desinfectante	◯	◯	◯	◯	◯
Cancelar o postergar viajes de trabajo	◯	◯	◯	◯	◯
Cancelar o postergar viajes de placer	◯	◯	◯	◯	◯
Cancelar o postergar viajes las actividades personales o sociales	◯	◯	◯	◯	◯
Cancelar las consultas médicas en persona	◯	◯	◯	◯	◯
Almacenar comida o agua	◯	◯	◯	◯	◯
Seguir las recomendaciones o reglas del gobierno sobre refugiarse donde se esté (es decir, quedarse en casa, limitar el contacto con otras personas, etc.)	◯	◯	◯	◯	◯

Durante los ultimos dos semanas, ha tenido alguno de los siguientes sintomas?

	Sí	No
Fiebre (medida por termómetro o autodiagnóstico)	◯	◯
Tos (nueva o que empeora)	◯	◯
Dificultad para respirar (nueva o que empeora)	◯	◯
Fatiga (nuevo cansancio al hacer actividades normales)	◯	◯
Dolores de cuerpo	◯	◯
Dolor de cabeza	◯	◯

Diarrea	○	○
Dolor de garganta	○	○
Picazón, enrojecimiento o dolor de ojos	○	○
Goteo o congestión nasal	○	○
Cambios en el sentido del olfato o del gusto	○	○
Nuevo sarpullido	○	○
Temblores y escalofríos reiterados	○	○

¿Cuándo comenzaron estos síntomas? __________________________________

¿Fue usted objeto de prejuicio o discriminación debido a sus síntomas?
- ○ Sí
- ○ No
- ○ No sabe

¿Cuáles de las siguientes medidas tomó usted para proteger a sus amigos y familiares después de que comenzaron sus síntomas?
- ☐ Ponerse mascarilla con mayor frecuencia
- ☐ Lavarse las manos con agua y jabón con mayor frecuencia
- ☐ Limpiarse las manos con desinfectante con mayor frecuencia
- ☐ Aislarse en su casa con mayor frecuencia
- ☐ Quedarse en casa con mayor frecuencia
- ☐ Ponerse guantes desechables con mayor frecuencia

¿Qué hizo usted en vista de sus síntomas?
- ☐ Nada
- ☐ Tomó medicamentos sin receta (ibuprofeno, acetaminofén, etc.)
- ☐ Consultó por teléfono a un proveedor de asistencia médica
- ☐ Fue al consultorio de un proveedor de asistencia
- ☐ Fue a una clínica o una farmacia minorista
- ☐ Fue a un centro de urgencias (FASTmed, etc.)
- ☐ Fue a la sala de emergencias
- ☐ Fue ingresado al hospital
- ☐ Otra cosa

((Seleccione todo lo que corresponda.))

¿Qué otra cosa hizo usted en vista de sus síntomas? __________________________________

Si pudo hablar con un proveedor de asistencia médica, ¿le dijo el proveedor que era posible que tuviera COVID-19?
- ○ Sí
- ○ No
- ○ No sabe

Si le hicieron una prueba de COVID-19 en vista de los síntomas que declaró, ¿cuál fue el resultado?
- ○ Pendiente
- ○ Positivo
- ○ Negativo
- ○ No concluyente
- ○ No se hizo la prueba

¿Cuántos días estuvo usted hospitalizado? __________________________________

¿Le hicieron a usted las siguientes intervenciones durante su hospitalización?	☐ Oxígeno adicional por la nariz<br>☐ Tratamiento en la Unidad de Cuidados Intensivos (Intensive Care Unit, ICU)<br>☐ Ventilación mecánica (intubación o tubo de respiración)
¿Ha vuelto usted a su salud normal?	○ Sí<br>○ No<br>○ No sabe
¿Ha participado o está participando actualmente en un ensayo de la vacuna de COVID?	○ Yes<br>○ No
¿Dónde fue o es el sitio principal para el ensayo de la vacuna de COVID? (Por ejemplo, ¿UNC, Duke o un hospital específico?)	__________________________
¿Ha recibido una vacuna de COVID-19 fuera de un ensayo clínico?	○ Sí<br>○ No
¿Dónde recibió la vacuna de COVID-19?	○ Oficina del doctor/medico<br>○ Trabajo/Empleo<br>○ Minorista (por ejemplo Walgreens, CVS)<br>○ Sitio de la vacuna<br>○ Otro
Especifique donde recibió la vacuna de COVID-19.	__________________________
Especifique la cuidad/el pueblo en Carolina del Norte del sitio de la vacuna:	__________________________
Especifique quien fue la organización o instituto que organizo el sitio de la vacuna:	__________________________
¿Cuál vacuna de COVID-19 recibió?	○ Pfizer<br>○ Moderna<br>○ AstraZeneca<br>○ Novavax<br>○ Johnson & Johnson<br>○ Otro<br>○ No sabe
Especifique que otra vacuna de COVID recibió.	__________________________
¿Cuántas dosis de la vacuna ha recibido?	○ 1<br>○ 2
¿Qué día recibió la primera dosis de la vacuna?	__________________________
¿Qué día recibió la segunda dosis de la vacuna?	__________________________

Did you experience any of the following side effects after vaccination?

		No	Leve (nota síntomas, pero no son un problema)	Moderado (los síntomas limitan sus actividades diarias normales)	Grave (los síntomas hacen que las actividades normales sean difícil o imposible)
1	dolor en o alrededor del lugar de la inyección	◯	◯	◯	◯
2	enrojecimiento en o alrededor del lugar de la inyección	◯	◯	◯	◯
3	hinchazón en o alrededor del lugar de la inyección	◯	◯	◯	◯
4	erupción en o alrededor del lugar de la inyección	◯	◯	◯	◯
5	dolor de cabeza	◯	◯	◯	◯
6	fatiga/cansancio	◯	◯	◯	◯
7	fiebre (temperatura >100.4°F o >38°C)	◯	◯	◯	◯
8	escalofríos	◯	◯	◯	◯
9	dolor en las articulaciones	◯	◯	◯	◯
10	dolor muscular	◯	◯	◯	◯
11	nausea	◯	◯	◯	◯

¿Cuánto tiempo duraron estos efectos secundarios?
- ◯ menos de 12 horas
- ◯ 12 a 24 horas
- ◯ más de 24 horas

¿Tomo algún medicamento para estos efectos secundarios?
- ◯ Sí
- ◯ No

¿Que medicamento(s) tomo para los efectos secundarios?

____
(Por favor enumere todos los medicamentos.)

¿Consultó a un médico u otro proveedor de atención médica para los efectos secundarios?
- ◯ Sí
- ◯ No

¿Como experimento los efectos secundarios después de la secunda dosis de la vacuna en comparación con los efectos secundarios después de la primera dosis de la vacuna?
- ◯ más grave
- ◯ menos grave
- ◯ igualmenta grave
- ◯ no corresponde/aún no he recibido la segunda dosis

Proporcione la siguiente informacion sobre su hogar.

¿Cuál es su dirección permanente?

____

¿Cuanto tiempo ha vivido en esta dirección?

- ◯ 0-3 años
- ◯ 4-6 años
- ◯ 7-10 años
- ◯ Más de 10 años

¿Cuántas personas (sin contarse usted) viven o pasan una cantidad considerable de tiempo en esta vivienda (más de 40 horas por semana)?

- ◯ 0
- ◯ 1
- ◯ 2
- ◯ 3
- ◯ 4
- ◯ 5
- ◯ 6
- ◯ 7
- ◯ 8
- ◯ 9
- ◯ 10
- ◯ 11
- ◯ 12

¿Cuántas personas de su grupo familiar son menores de 18 años?

- ◯ 0
- ◯ 1
- ◯ 2
- ◯ 3
- ◯ 4
- ◯ 5
- ◯ 6
- ◯ 7
- ◯ 8
- ◯ 9
- ◯ 10
- ◯ 11
- ◯ 12

Por cada persona adicional en su hogar, proporcione la siguiente informacion.

Persona 1: ¿Cuál es su relación con esta persona?

- ◯ Pareja o cónyuge
- ◯ Hijo o hija
- ◯ Padre o madre
- ◯ Hermano o hermana
- ◯ Otro familiar
- ◯ Proveedor de cuidado infantil u otros cuidados en casa
- ◯ Otra

Persona 1: Especifique su relación con esta persona.

____

Persona 1: ¿Qué edad tiene esta persona?

____
((Especifique la edad en años))

Persona 1: ¿Cuál es el sexo de esta persona?

- ◯ Femenino
- ◯ Masculino

Persona 1: ¿Cuál es la raza de esta persona?	☐ Indio americano o nativo de Alaska<br>☐ Asiático<br>☐ Negro o afroestadounidense<br>☐ Nativo de Hawái o de las islas del Pacífico<br>☐ Blanco<br>☐ Otra<br>☐ No sabe<br>((Seleccione todo lo que corresponda.))
Persona 1: ¿Cuál es la identidad étnica de esta persona?	◯ Hispano o latino<br>◯ Ni hispano ni latino<br>◯ Otra<br>◯ No sabe
Persona 1: ¿Cuál es el nivel de educación de esta persona?	◯ No tiene educación formal<br>◯ Kínder a 8° grado<br>◯ Estudios de educación secundaria<br>◯ Equivalencia de educación secundaria (GED)<br>◯ Diploma de educación secundaria<br>◯ Estudios de educación superior<br>◯ Título universitario<br>◯ Estudios de posgrado o más<br>◯ No sabe
Persona 1: ¿Cuál de las siguientes es la mejor descripción de la situación laboral actual de esta persona?	◯ Empleo a tiempo completo<br>◯ Empleo a tiempo parcial<br>◯ En busca de empleo<br>◯ Jubilado<br>◯ Ama de casa<br>◯ Estudiante<br>◯ Permiso de maternidad o paternidad<br>◯ Permiso por enfermedad<br>◯ Desempleado por discapacidad<br>◯ Otra<br>◯ No sabe
Persona 1: ¿Se considera esta persona actualmente empleado por cuenta propia (contratista independiente, trabajador esporádico (gig), etc.)?	◯ Sí<br>◯ No<br>◯ No sabe
Persona 1: ¿Trabaja esta persona actualmente en alguno de los siguientes ambientes de alto riesgo de contagio de COVID-19?	☐ Ambiente de asistencia médica (hospital, clínica, centro de urgencias, etc.)<br>☐ Ambiente residencial denso (hogar de ancianos, otro centro de asistencia de larga duración)<br>☐ Prisión o cárcel<br>☐ Establecimiento de envasado de carne<br>☐ Establecimiento de envío o distribución<br>☐ Establecimiento minorista de alto volumen (tienda de provisiones, etc.)<br>☐ No sabe
Persona 1: ¿Le ofrece el empleador a esta persona alguno de los siguientes beneficios en su empleo principal actual?	☐ Permiso por enfermedad con goce de sueldo<br>☐ Vacaciones o permiso personal con goce de sueldo<br>☐ Seguro de salud<br>☐ Seguro de discapacidad<br>☐ Plan de jubilación<br>☐ Otro<br>☐ No sabe<br>((Seleccione todo lo que corresponda.))

Persona 1: En una escala de 0 (definitivamente no va a suceder) a 10 (definitivamente va a suceder), ¿qué tan probable es que esta persona pierda su empleo debido a la pandemia de COVID-19? ______________________________

Persona 1: En una escala de 0 (definitivamente no va a suceder) a 10 (definitivamente va a suceder), ¿qué tan probable es que a esta persona se le asignen menos horas de trabajo debido a la pandemia de COVID-19? ______________________________

	Todo el tiempo (100%)	La mayor parte del tiempo (75%)	La mitad del tiempo (50%)	Menos de la mitad del tiempo (25%)	nunca (0%)	Nunca
Persona 1: Actualmente ¿con qué frecuencia se le exige a esta persona que trabaje fuera de su residencia?	◯	◯	◯	◯	◯	◯
Persona 1: Actualmente, ¿con qué frecuencia se encuentra esta persona físicamente cerca de sus compañeros de trabajo mientras trabaja fuera de su residencia?	◯	◯	◯	◯	◯	◯
Persona 1: Actualmente, ¿con qué frecuencia se encuentra esta persona físicamente cerca de sus clientes mientras trabaja fuera de su residencia	◯	◯	◯	◯	◯	◯

Persona 1: ¿Piensa esta persona vacunarse contra la COVID-19 cuando se ofrezca una vacuna?
- ◯ Sí
- ◯ No
- ◯ No sabe

Persona 1: En las últimas dos semanas, ¿ha tenido esta persona algún síntoma de COVID-19 (tos, fiebre, dificultad para respirar, fatiga, dolores de cuerpo, diarrea, goteo nasal o pérdida del sentido del olfato o del gusto)?
- ◯ Sí
- ◯ No
- ◯ No sabe

Persona 1: ¿Cuándo le comenzaron los síntomas de COVID-19 a esta persona? ______________________________

Persona 1: En vista de sus síntomas, ¿le preocupa a esta persona la posibilidad de tener COVID-19?
- ◯ Sí
- ◯ No
- ◯ No sabe

Persona 1: ¿Fue esta persona objeto de prejuicio o discriminación debido a sus síntomas?
- ◯ Sí
- ◯ No
- ◯ No sabe

Persona 1: ¿Qué hizo esta persona en vista de sus síntomas?	☐ Nada<br>☐ Tomó medicamentos sin receta (ibuprofeno, acetaminofén, etc.)<br>☐ Consultó por teléfono a un proveedor de asistencia médica<br>☐ Fue al consultorio de un proveedor de asistencia médica<br>☐ Fue a una clínica o una farmacia minorista<br>☐ Fue a un centro de urgencias (FASTMed, etc.)<br>☐ Fue a la sala de emergencias<br>☐ Fue ingresado al hospital<br>☐ Otra cosa<br>☐ No sabe<br>((Seleccione todo lo que corresponda.))
Persona 1: ¿Qué otra cosa hizo esta persona en vista de sus síntomas?	______________________________
Persona 1: ¿Le dijo un proveedor de asistencia médica a esta persona que era posible que tuviera COVID-19?	◯ Sí<br>◯ No<br>◯ No sabe
Persona 1: Si a esta persona le hicieron una prueba de COVID-19 en vista de sus síntomas, ¿cuál fue el resultado?	◯ Pendiente<br>◯ Positivo<br>◯ Negativo<br>◯ No concluyente<br>◯ No se hizo la prueba<br>◯ No sabe
Persona 1: ¿Cuántos días estuvo hospitalizada esta persona?	______________________________
Persona 1: ¿Se le hicieron a esta persona las siguientes intervenciones durante su hospitalización?	☐ Oxígeno adicional por la nariz<br>☐ Tratamiento en la Unidad de Cuidados Intensivos (Intensive Care Unit, ICU)<br>☐ Ventilación mecánica (intubación o tubo de respiración)<br>☐ No sabe
Persona 1: ¿Ha vuelto esta persona a su salud normal?	◯ Sí<br>◯ No<br>◯ No sabe
Persona 1: ¿Cuáles de las siguientes medidas tomó esta persona para proteger a sus amigos y familiares después de que comenzaron sus síntomas?	☐ Ponerse mascarilla con mayor frecuencia<br>☐ Lavarse las manos con agua y jabón con mayor frecuencia<br>☐ Limpiarse las manos con desinfectante con mayor frecuencia<br>☐ Aislarse en su casa con mayor frecuencia<br>☐ Quedarse en casa con mayor frecuencia<br>☐ Ponerse guantes desechables con mayor frecuencia<br>☐ No sabe

Por cada persona adicional en su hogar, proporcione la siguiente informacion.

Persona 2: ¿Cuál es su relación con esta persona?	◯ Pareja o cónyuge<br>◯ Hijo o hija<br>◯ Padre o madre<br>◯ Hermano o hermana<br>◯ Otro familiar<br>◯ Proveedor de cuidado infantil u otros cuidados en casa<br>◯ Otra
Persona 2: Especifique su relación con esta persona.	__________________________________
Persona 2: ¿Qué edad tiene esta persona?	__________________________________<br>((Especifique la edad en años))
Persona 2: ¿Cuál es el sexo de esta persona?	◯ Femenino<br>◯ Masculino
Persona 2: ¿Cuál es la raza de esta persona?	☐ Indio americano o nativo de Alaska<br>☐ Asiático<br>☐ Negro o afroestadounidense<br>☐ Nativo de Hawái o de las islas del Pacífico<br>☐ Blanco<br>☐ Otra<br>☐ No sabe<br>((Seleccione todo lo que corresponda.))
Persona 2: ¿Cuál es la identidad étnica de esta persona?	◯ Hispano o latino<br>◯ Ni hispano ni latino<br>◯ Otra<br>◯ No sabe
Persona 2: ¿Cuál es el nivel de educación de esta persona?	◯ No tiene educación formal<br>◯ Kínder a 8° grado<br>◯ Estudios de educación secundaria<br>◯ Equivalencia de educación secundaria (GED)<br>◯ Diploma de educación secundaria<br>◯ Estudios de educación superior<br>◯ Título universitario<br>◯ Estudios de posgrado o más<br>◯ No sabe
Persona 2: ¿Cuál de las siguientes es la mejor descripción de la situación laboral actual de esta persona?	◯ Empleo a tiempo completo<br>◯ Empleo a tiempo parcial<br>◯ En busca de empleo<br>◯ Jubilado<br>◯ Ama de casa<br>◯ Estudiante<br>◯ Permiso de maternidad o paternidad<br>◯ Permiso por enfermedad<br>◯ Desempleado por discapacidad<br>◯ Otra<br>◯ No sabe
Persona 2: ¿Se considera esta persona actualmente empleado por cuenta propia (contratista independiente, trabajador esporádico (gig), etc.)?	◯ Sí<br>◯ No<br>◯ No sabe

Persona 2: ¿Trabaja esta persona actualmente en alguno de los siguientes ambientes de alto riesgo de contagio de COVID-19?

☐ Ambiente de asistencia médica (hospital, clínica, centro de urgencias, etc.)
☐ Ambiente residencial denso (hogar de ancianos, otro centro de asistencia de larga duración)
☐ Prisión o cárcel
☐ Establecimiento de envasado de carne
☐ Establecimiento de envío o distribución
☐ Establecimiento minorista de alto volumen (tienda de provisiones, etc.)
☐ No sabe

Persona 2: ¿Le ofrece el empleador a esta persona alguno de los siguientes beneficios en su empleo principal actual?

☐ Permiso por enfermedad con goce de sueldo
☐ Vacaciones o permiso personal con goce de sueldo
☐ Seguro de salud
☐ Seguro de discapacidad
☐ Plan de jubilación
☐ Otro
☐ No sabe
((Seleccione todo lo que corresponda.))

Persona 2: En una escala de 0 (definitivamente no va a suceder) a 10 (definitivamente va a suceder), ¿qué tan probable es que esta persona pierda su empleo debido a la pandemia de COVID-19?

____

Persona 2: En una escala de 0 (definitivamente no va a suceder) a 10 (definitivamente va a suceder), ¿qué tan probable es que a esta persona se le asignen menos horas de trabajo debido a la pandemia de COVID-19?

____

	Todo el tiempo (100%)	La mayor parte del tiempo (75%)	La mitad del tiempo (50%)	Menos de la mitad del tiempo (25%)	nunca (0%)	Nunca
Persona 2: Actualmente ¿con qué frecuencia se le exige a esta persona que trabaje fuera de su residencia?	○	○	○	○	○	○
Persona 2: Actualmente, ¿con qué frecuencia se encuentra esta persona físicamente cerca de sus compañeros de trabajo mientras trabaja fuera de su residencia?	○	○	○	○	○	○
Persona 2: Actualmente, ¿con qué frecuencia se encuentra esta persona físicamente cerca de sus clientes mientras trabaja fuera de su residencia	○	○	○	○	○	○

Persona 2: ¿Piensa esta persona vacunarse contra la COVID-19 cuando se ofrezca una vacuna?

○ Sí
○ No
○ No sabe

Persona 2: En las últimas dos semanas, ¿ha tenido esta persona algún síntoma de COVID-19 (tos, fiebre, dificultad para respirar, fatiga, dolores de cuerpo, diarrea, goteo nasal o pérdida del sentido del olfato o del gusto)?	◯ Sí<br>◯ No<br>◯ No sabe
Persona 2: ¿Cuándo le comenzaron los síntomas de COVID-19 a esta persona?	______________________
Persona 2: En vista de sus síntomas, ¿le preocupa a esta persona la posibilidad de tener COVID-19?	◯ Sí<br>◯ No<br>◯ No sabe
Persona 2: ¿Fue esta persona objeto de prejuicio o discriminación debido a sus síntomas?	◯ Sí<br>◯ No<br>◯ No sabe
Persona 2: ¿Qué hizo esta persona en vista de sus síntomas?	☐ Nada<br>☐ Tomó medicamentos sin receta (ibuprofeno, acetaminofén, etc.)<br>☐ Consultó por teléfono a un proveedor de asistencia médica<br>☐ Fue al consultorio de un proveedor de asistencia médica<br>☐ Fue a una clínica o una farmacia minorista<br>☐ Fue a un centro de urgencias (FASTMed, etc.)<br>☐ Fue a la sala de emergencias<br>☐ Fue ingresado al hospital<br>☐ Otra cosa<br>☐ No sabe<br>((Seleccione todo lo que corresponda.))
Persona 2: ¿Qué otra cosa hizo esta persona en vista de sus síntomas?	______________________
Persona 2: ¿Le dijo un proveedor de asistencia médica a esta persona que era posible que tuviera COVID-19?	◯ Sí<br>◯ No<br>◯ No sabe
Persona 2: Si a esta persona le hicieron una prueba de COVID-19 en vista de sus síntomas, ¿cuál fue el resultado?	◯ Pendiente<br>◯ Positivo<br>◯ Negativo<br>◯ No concluyente<br>◯ No se hizo la prueba<br>◯ No sabe
Persona 2: ¿Cuántos días estuvo hospitalizada esta persona?	______________________
Persona 2: ¿Se le hicieron a esta persona las siguientes intervenciones durante su hospitalización?	☐ Oxígeno adicional por la nariz<br>☐ Tratamiento en la Unidad de Cuidados Intensivos (Intensive Care Unit, ICU)<br>☐ Ventilación mecánica (intubación o tubo de respiración)<br>☐ No sabe
Persona 2: ¿Ha vuelto esta persona a su salud normal?	◯ Sí<br>◯ No<br>◯ No sabe

Persona 2: ¿Cuáles de las siguientes medidas tomó esta persona para proteger a sus amigos y familiares después de que comenzaron sus síntomas?

- ☐ Ponerse mascarilla con mayor frecuencia
- ☐ Lavarse las manos con agua y jabón con mayor frecuencia
- ☐ Limpiarse las manos con desinfectante con mayor frecuencia
- ☐ Aislarse en su casa con mayor frecuencia
- ☐ Quedarse en casa con mayor frecuencia
- ☐ Ponerse guantes desechables con mayor frecuencia
- ☐ No sabe

Por cada persona adicional en su hogar, proporcione la siguiente informacion.

Persona 3: ¿Cuál es su relación con esta persona?

- ○ Pareja o cónyuge
- ○ Hijo o hija
- ○ Padre o madre
- ○ Hermano o hermana
- ○ Otro familiar
- ○ Proveedor de cuidado infantil u otros cuidados en casa
- ○ Otra

Persona 3: Especifique su relación con esta persona.

____

Persona 3: ¿Qué edad tiene esta persona?

____
((Especifique la edad en años))

Persona 3: ¿Cuál es el sexo de esta persona?

- ○ Femenino
- ○ Masculino

Persona 3: ¿Cuál es la raza de esta persona?

- ☐ Indio americano o nativo de Alaska
- ☐ Asiático
- ☐ Negro o afroestadounidense
- ☐ Nativo de Hawái o de las islas del Pacífico
- ☐ Blanco
- ☐ Otra
- ☐ No sabe

((Seleccione todo lo que corresponda.))

Persona 3: ¿Cuál es la identidad étnica de esta persona?

- ○ Hispano o latino
- ○ Ni hispano ni latino
- ○ Otra
- ○ No sabe

Persona 3: ¿Cuál es el nivel de educación de esta persona?

- ○ No tiene educación formal
- ○ Kínder a 8° grado
- ○ Estudios de educación secundaria
- ○ Equivalencia de educación secundaria (GED)
- ○ Diploma de educación secundaria
- ○ Estudios de educación superior
- ○ Título universitario
- ○ Estudios de posgrado o más
- ○ No sabe

Persona 3: ¿Cuál de las siguientes es la mejor descripción de la situación laboral actual de esta persona?	◯ Empleo a tiempo completo<br>◯ Empleo a tiempo parcial<br>◯ En busca de empleo<br>◯ Jubilado<br>◯ Ama de casa<br>◯ Estudiante<br>◯ Permiso de maternidad o paternidad<br>◯ Permiso por enfermedad<br>◯ Desempleado por discapacidad<br>◯ Otra<br>◯ No sabe
Persona 3: ¿Se considera esta persona actualmente empleado por cuenta propia (contratista independiente, trabajador esporádico (gig), etc.)?	◯ Sí<br>◯ No<br>◯ No sabe
Persona 3: ¿Trabaja esta persona actualmente en alguno de los siguientes ambientes de alto riesgo de contagio de COVID-19?	☐ Ambiente de asistencia médica (hospital, clínica, centro de urgencias, etc.)<br>☐ Ambiente residencial denso (hogar de ancianos, otro centro de asistencia de larga duración)<br>☐ Prisión o cárcel<br>☐ Establecimiento de envasado de carne<br>☐ Establecimiento de envío o distribución<br>☐ Establecimiento minorista de alto volumen (tienda de provisiones, etc.)<br>☐ No sabe
Persona 3: ¿Le ofrece el empleador a esta persona alguno de los siguientes beneficios en su empleo principal actual?	☐ Permiso por enfermedad con goce de sueldo<br>☐ Vacaciones o permiso personal con goce de sueldo<br>☐ Seguro de salud<br>☐ Seguro de discapacidad<br>☐ Plan de jubilación<br>☐ Otro<br>☐ No sabe<br>((Seleccione todo lo que corresponda.))
Persona 3: En una escala de 0 (definitivamente no va a suceder) a 10 (definitivamente va a suceder), ¿qué tan probable es que esta persona pierda su empleo debido a la pandemia de COVID-19?	______________________________
Persona 3: En una escala de 0 (definitivamente no va a suceder) a 10 (definitivamente va a suceder), ¿qué tan probable es que a esta persona se le asignen menos horas de trabajo debido a la pandemia de COVID-19?	______________________________

	Todo el tiempo (100%)	La mayor parte del tiempo (75%)	La mitad del tiempo (50%)	Menos de la mitad del tiempo (25%)	nunca (0%)	Nunca
Persona 3: Actualmente ¿con qué frecuencia se le exige a esta persona que trabaje fuera de su residencia?	◯	◯	◯	◯	◯	◯

Persona 3: Actualmente, ¿con qué frecuencia se encuentra esta persona físicamente cerca de sus compañeros de trabajo mientras trabaja fuera de su residencia? ○ ○ ○ ○ ○ ○

Persona 3: Actualmente, ¿con qué frecuencia se encuentra esta persona físicamente cerca de sus clientes mientras trabaja fuera de su residencia ○ ○ ○ ○ ○ ○

Persona 3: ¿Piensa esta persona vacunarse contra la COVID-19 cuando se ofrezca una vacuna?

○ Sí
○ No
○ No sabe

Persona 3: En las últimas dos semanas, ¿ha tenido esta persona algún síntoma de COVID-19 (tos, fiebre, dificultad para respirar, fatiga, dolores de cuerpo, diarrea, goteo nasal o pérdida del sentido del olfato o del gusto)?

○ Sí
○ No
○ No sabe

Persona 3: ¿Cuándo le comenzaron los síntomas de COVID-19 a esta persona?

____

Persona 3: En vista de sus síntomas, ¿le preocupa a esta persona la posibilidad de tener COVID-19?

○ Sí
○ No
○ No sabe

Persona 3: ¿Fue esta persona objeto de prejuicio o discriminación debido a sus síntomas?

○ Sí
○ No
○ No sabe

Persona 3: ¿Qué hizo esta persona en vista de sus síntomas?

☐ Nada
☐ Tomó medicamentos sin receta (ibuprofeno, acetaminofén, etc.)
☐ Consultó por teléfono a un proveedor de asistencia médica
☐ Fue al consultorio de un proveedor de asistencia médica
☐ Fue a una clínica o una farmacia minorista
☐ Fue a un centro de urgencias (FASTMed, etc.)
☐ Fue a la sala de emergencias
☐ Fue ingresado al hospital
☐ Otra cosa
☐ No sabe
((Seleccione todo lo que corresponda.))

Persona 3: ¿Qué otra cosa hizo esta persona en vista de sus síntomas?

____

Persona 3: ¿Le dijo un proveedor de asistencia médica a esta persona que era posible que tuviera COVID-19?

○ Sí
○ No
○ No sabe

Persona 3: Si a esta persona le hicieron una prueba de COVID-19 en vista de sus síntomas, ¿cuál fue el resultado?	◯ Pendiente<br>◯ Positivo<br>◯ Negativo<br>◯ No concluyente<br>◯ No se hizo la prueba<br>◯ No sabe
Persona 3: ¿Cuántos días estuvo hospitalizada esta persona?	______________________________
Persona 3: ¿Se le hicieron a esta persona las siguientes intervenciones durante su hospitalización?	☐ Oxígeno adicional por la nariz<br>☐ Tratamiento en la Unidad de Cuidados Intensivos (Intensive Care Unit, ICU)<br>☐ Ventilación mecánica (intubación o tubo de respiración)<br>☐ No sabe
Persona 3: ¿Ha vuelto esta persona a su salud normal?	◯ Sí<br>◯ No<br>◯ No sabe
Persona 3: ¿Cuáles de las siguientes medidas tomó esta persona para proteger a sus amigos y familiares después de que comenzaron sus síntomas?	☐ Ponerse mascarilla con mayor frecuencia<br>☐ Lavarse las manos con agua y jabón con mayor frecuencia<br>☐ Limpiarse las manos con desinfectante con mayor frecuencia<br>☐ Aislarse en su casa con mayor frecuencia<br>☐ Quedarse en casa con mayor frecuencia<br>☐ Ponerse guantes desechables con mayor frecuencia<br>☐ No sabe

Por cada persona adicional en su hogar, proporcione la siguiente informacion.

Persona 4: ¿Cuál es su relación con esta persona?	◯ Pareja o cónyuge<br>◯ Hijo o hija<br>◯ Padre o madre<br>◯ Hermano o hermana<br>◯ Otro familiar<br>◯ Proveedor de cuidado infantil u otros cuidados en casa<br>◯ Otra
Persona 4: Especifique su relación con esta persona.	______________________________
Persona 4: ¿Qué edad tiene esta persona?	______________________________<br>((Especifique la edad en años))
Persona 4: ¿Cuál es el sexo de esta persona?	◯ Femenino<br>◯ Masculino

Persona 4: ¿Cuál es la raza de esta persona?	☐ Indio americano o nativo de Alaska<br>☐ Asiático<br>☐ Negro o afroestadounidense<br>☐ Nativo de Hawái o de las islas del Pacífico<br>☐ Blanco<br>☐ Otra<br>☐ No sabe<br>((Seleccione todo lo que corresponda.))
Persona 4: ¿Cuál es la identidad étnica de esta persona?	◯ Hispano o latino<br>◯ Ni hispano ni latino<br>◯ Otra<br>◯ No sabe
Persona 4: ¿Cuál es el nivel de educación de esta persona?	◯ No tiene educación formal<br>◯ Kínder a 8° grado<br>◯ Estudios de educación secundaria<br>◯ Equivalencia de educación secundaria (GED)<br>◯ Diploma de educación secundaria<br>◯ Estudios de educación superior<br>◯ Título universitario<br>◯ Estudios de posgrado o más<br>◯ No sabe
Persona 4: ¿Cuál de las siguientes es la mejor descripción de la situación laboral actual de esta persona?	◯ Empleo a tiempo completo<br>◯ Empleo a tiempo parcial<br>◯ En busca de empleo<br>◯ Jubilado<br>◯ Ama de casa<br>◯ Estudiante<br>◯ Permiso de maternidad o paternidad<br>◯ Permiso por enfermedad<br>◯ Desempleado por discapacidad<br>◯ Otra<br>◯ No sabe
Persona 4: ¿Se considera esta persona actualmente empleado por cuenta propia (contratista independiente, trabajador esporádico (gig), etc.)?	◯ Sí<br>◯ No<br>◯ No sabe
Persona 4: ¿Trabaja esta persona actualmente en alguno de los siguientes ambientes de alto riesgo de contagio de COVID-19?	☐ Ambiente de asistencia médica (hospital, clínica, centro de urgencias, etc.)<br>☐ Ambiente residencial denso (hogar de ancianos, otro centro de asistencia de larga duración)<br>☐ Prisión o cárcel<br>☐ Establecimiento de envasado de carne<br>☐ Establecimiento de envío o distribución<br>☐ Establecimiento minorista de alto volumen (tienda de provisiones, etc.)<br>☐ No sabe
Persona 4: ¿Le ofrece el empleador a esta persona alguno de los siguientes beneficios en su empleo principal actual?	☐ Permiso por enfermedad con goce de sueldo<br>☐ Vacaciones o permiso personal con goce de sueldo<br>☐ Seguro de salud<br>☐ Seguro de discapacidad<br>☐ Plan de jubilación<br>☐ Otro<br>☐ No sabe<br>((Seleccione todo lo que corresponda.))

Persona 4: En una escala de 0 (definitivamente no va a suceder) a 10 (definitivamente va a suceder), ¿qué tan probable es que esta persona pierda su empleo debido a la pandemia de COVID-19? ________________________________

Persona 4: En una escala de 0 (definitivamente no va a suceder) a 10 (definitivamente va a suceder), ¿qué tan probable es que a esta persona se le asignen menos horas de trabajo debido a la pandemia de COVID-19? ________________________________

	Todo el tiempo (100%)	La mayor parte del tiempo (75%)	La mitad del tiempo (50%)	Menos de la mitad del tiempo (25%)	nunca (0%)	Nunca
Persona 4: Actualmente ¿con qué frecuencia se le exige a esta persona que trabaje fuera de su residencia?	◯	◯	◯	◯	◯	◯
Persona 4: Actualmente, ¿con qué frecuencia se encuentra esta persona físicamente cerca de sus compañeros de trabajo mientras trabaja fuera de su residencia?	◯	◯	◯	◯	◯	◯
Persona 4: Actualmente, ¿con qué frecuencia se encuentra esta persona físicamente cerca de sus clientes mientras trabaja fuera de su residencia	◯	◯	◯	◯	◯	◯

Persona 4: ¿Piensa esta persona vacunarse contra la COVID-19 cuando se ofrezca una vacuna?

◯ Sí
◯ No
◯ No sabe

Persona 4: En las últimas dos semanas, ¿ha tenido esta persona algún síntoma de COVID-19 (tos, fiebre, dificultad para respirar, fatiga, dolores de cuerpo, diarrea, goteo nasal o pérdida del sentido del olfato o del gusto)?

◯ Sí
◯ No
◯ No sabe

Persona 4: ¿Cuándo le comenzaron los síntomas de COVID-19 a esta persona? ________________________________

Persona 4: En vista de sus síntomas, ¿le preocupa a esta persona la posibilidad de tener COVID-19?

◯ Sí
◯ No
◯ No sabe

Persona 4: ¿Fue esta persona objeto de prejuicio o discriminación debido a sus síntomas?

◯ Sí
◯ No
◯ No sabe

Persona 4: ¿Qué hizo esta persona en vista de sus síntomas?	☐ Nada<br>☐ Tomó medicamentos sin receta (ibuprofeno, acetaminofén, etc.)<br>☐ Consultó por teléfono a un proveedor de asistencia médica<br>☐ Fue al consultorio de un proveedor de asistencia médica<br>☐ Fue a una clínica o una farmacia minorista<br>☐ Fue a un centro de urgencias (FASTMed, etc.)<br>☐ Fue a la sala de emergencias<br>☐ Fue ingresado al hospital<br>☐ Otra cosa<br>☐ No sabe<br>((Seleccione todo lo que corresponda.))
Persona 4: ¿Qué otra cosa hizo esta persona en vista de sus síntomas?	______________________________
Persona 4: ¿Le dijo un proveedor de asistencia médica a esta persona que era posible que tuviera COVID-19?	◯ Sí<br>◯ No<br>◯ No sabe
Persona 4: Si a esta persona le hicieron una prueba de COVID-19 en vista de sus síntomas, ¿cuál fue el resultado?	◯ Pendiente<br>◯ Positivo<br>◯ Negativo<br>◯ No concluyente<br>◯ No se hizo la prueba<br>◯ No sabe
Persona 4: ¿Cuántos días estuvo hospitalizada esta persona?	______________________________
Persona 4: ¿Se le hicieron a esta persona las siguientes intervenciones durante su hospitalización?	☐ Oxígeno adicional por la nariz<br>☐ Tratamiento en la Unidad de Cuidados Intensivos (Intensive Care Unit, ICU)<br>☐ Ventilación mecánica (intubación o tubo de respiración)<br>☐ No sabe
Persona 4: ¿Ha vuelto esta persona a su salud normal?	◯ Sí<br>◯ No<br>◯ No sabe
Persona 4: ¿Cuáles de las siguientes medidas tomó esta persona para proteger a sus amigos y familiares después de que comenzaron sus síntomas?	☐ Ponerse mascarilla con mayor frecuencia<br>☐ Lavarse las manos con agua y jabón con mayor frecuencia<br>☐ Limpiarse las manos con desinfectante con mayor frecuencia<br>☐ Aislarse en su casa con mayor frecuencia<br>☐ Quedarse en casa con mayor frecuencia<br>☐ Ponerse guantes desechables con mayor frecuencia<br>☐ No sabe

Por cada persona adicional en su hogar, proporcione la siguiente informacion.

Persona 5: ¿Cuál es su relación con esta persona?

- ◯ Pareja o cónyuge
- ◯ Hijo o hija
- ◯ Padre o madre
- ◯ Hermano o hermana
- ◯ Otro familiar
- ◯ Proveedor de cuidado infantil u otros cuidados en casa
- ◯ Otra

Persona 5: Especifique su relación con esta persona.

____

Persona 5: ¿Qué edad tiene esta persona?

____
((Especifique la edad en años))

Persona 5: ¿Cuál es el sexo de esta persona?

- ◯ Femenino
- ◯ Masculino

Persona 5: ¿Cuál es la raza de esta persona?

- ☐ Indio americano o nativo de Alaska
- ☐ Asiático
- ☐ Negro o afroestadounidense
- ☐ Nativo de Hawái o de las islas del Pacífico
- ☐ Blanco
- ☐ Otra
- ☐ No sabe

((Seleccione todo lo que corresponda.))

Persona 5: ¿Cuál es la identidad étnica de esta persona?

- ◯ Hispano o latino
- ◯ Ni hispano ni latino
- ◯ Otra
- ◯ No sabe

Persona 5: ¿Cuál es el nivel de educación de esta persona?

- ◯ No tiene educación formal
- ◯ Kínder a 8° grado
- ◯ Estudios de educación secundaria
- ◯ Equivalencia de educación secundaria (GED)
- ◯ Diploma de educación secundaria
- ◯ Estudios de educación superior
- ◯ Título universitario
- ◯ Estudios de posgrado o más
- ◯ No sabe

Persona 5: ¿Cuál de las siguientes es la mejor descripción de la situación laboral actual de esta persona?

- ◯ Empleo a tiempo completo
- ◯ Empleo a tiempo parcial
- ◯ En busca de empleo
- ◯ Jubilado
- ◯ Ama de casa
- ◯ Estudiante
- ◯ Permiso de maternidad o paternidad
- ◯ Permiso por enfermedad
- ◯ Desempleado por discapacidad
- ◯ Otra
- ◯ No sabe

Persona 5: ¿Se considera esta persona actualmente empleado por cuenta propia (contratista independiente, trabajador esporádico (gig), etc.)?

- ◯ Sí
- ◯ No
- ◯ No sabe

Persona 5: ¿Trabaja esta persona actualmente en alguno de los siguientes ambientes de alto riesgo de contagio de COVID-19?	☐ Ambiente de asistencia médica (hospital, clínica, centro de urgencias, etc.)<br>☐ Ambiente residencial denso (hogar de ancianos, otro centro de asistencia de larga duración)<br>☐ Prisión o cárcel<br>☐ Establecimiento de envasado de carne<br>☐ Establecimiento de envío o distribución<br>☐ Establecimiento minorista de alto volumen (tienda de provisiones, etc.)<br>☐ No sabe
Persona 5: ¿Le ofrece el empleador a esta persona alguno de los siguientes beneficios en su empleo principal actual?	☐ Permiso por enfermedad con goce de sueldo<br>☐ Vacaciones o permiso personal con goce de sueldo<br>☐ Seguro de salud<br>☐ Seguro de discapacidad<br>☐ Plan de jubilación<br>☐ Otro<br>☐ No sabe<br>((Seleccione todo lo que corresponda.))
Persona 5: En una escala de 0 (definitivamente no va a suceder) a 10 (definitivamente va a suceder), ¿qué tan probable es que esta persona pierda su empleo debido a la pandemia de COVID-19?	______________________
Persona 5: En una escala de 0 (definitivamente no va a suceder) a 10 (definitivamente va a suceder), ¿qué tan probable es que a esta persona se le asignen menos horas de trabajo debido a la pandemia de COVID-19?	______________________

	Todo el tiempo (100%)	La mayor parte del tiempo (75%)	La mitad del tiempo (50%)	Menos de la mitad del tiempo (25%)	nunca (0%)	Nunca
Persona 5: Actualmente ¿con qué frecuencia se le exige a esta persona que trabaje fuera de su residencia?	◯	◯	◯	◯	◯	◯
Persona 5: Actualmente, ¿con qué frecuencia se encuentra esta persona físicamente cerca de sus compañeros de trabajo mientras trabaja fuera de su residencia?	◯	◯	◯	◯	◯	◯
Persona 5: Actualmente, ¿con qué frecuencia se encuentra esta persona físicamente cerca de sus clientes mientras trabaja fuera de su residencia	◯	◯	◯	◯	◯	◯

Persona 5: ¿Piensa esta persona vacunarse contra la COVID-19 cuando se ofrezca una vacuna?	◯ Sí<br>◯ No<br>◯ No sabe

Persona 5: En las últimas dos semanas, ¿ha tenido esta persona algún síntoma de COVID-19 (tos, fiebre, dificultad para respirar, fatiga, dolores de cuerpo, diarrea, goteo nasal o pérdida del sentido del olfato o del gusto)?	◯ Sí<br>◯ No<br>◯ No sabe
Persona 5: ¿Cuándo le comenzaron los síntomas de COVID-19 a esta persona?	__________________________________
Persona 5: En vista de sus síntomas, ¿le preocupa a esta persona la posibilidad de tener COVID-19?	◯ Sí<br>◯ No<br>◯ No sabe
Persona 5: ¿Fue esta persona objeto de prejuicio o discriminación debido a sus síntomas?	◯ Sí<br>◯ No<br>◯ No sabe
Persona 5: ¿Qué hizo esta persona en vista de sus síntomas?	☐ Nada<br>☐ Tomó medicamentos sin receta (ibuprofeno, acetaminofén, etc.)<br>☐ Consultó por teléfono a un proveedor de asistencia médica<br>☐ Fue al consultorio de un proveedor de asistencia médica<br>☐ Fue a una clínica o una farmacia minorista<br>☐ Fue a un centro de urgencias (FASTMed, etc.)<br>☐ Fue a la sala de emergencias<br>☐ Fue ingresado al hospital<br>☐ Otra cosa<br>☐ No sabe<br>((Seleccione todo lo que corresponda.))
Persona 5: ¿Qué otra cosa hizo esta persona en vista de sus síntomas?	__________________________________
Persona 5: ¿Le dijo un proveedor de asistencia médica a esta persona que era posible que tuviera COVID-19?	◯ Sí<br>◯ No<br>◯ No sabe
Persona 5: Si a esta persona le hicieron una prueba de COVID-19 en vista de sus síntomas, ¿cuál fue el resultado?	◯ Pendiente<br>◯ Positivo<br>◯ Negativo<br>◯ No concluyente<br>◯ No se hizo la prueba<br>◯ No sabe
Persona 5: ¿Cuántos días estuvo hospitalizada esta persona?	__________________________________
Persona 5: ¿Se le hicieron a esta persona las siguientes intervenciones durante su hospitalización?	☐ Oxígeno adicional por la nariz<br>☐ Tratamiento en la Unidad de Cuidados Intensivos (Intensive Care Unit, ICU)<br>☐ Ventilación mecánica (intubación o tubo de respiración)<br>☐ No sabe
Persona 5: ¿Ha vuelto esta persona a su salud normal?	◯ Sí<br>◯ No<br>◯ No sabe

Persona 5: ¿Cuáles de las siguientes medidas tomó esta persona para proteger a sus amigos y familiares después de que comenzaron sus síntomas?

- ☐ Ponerse mascarilla con mayor frecuencia
- ☐ Lavarse las manos con agua y jabón con mayor frecuencia
- ☐ Limpiarse las manos con desinfectante con mayor frecuencia
- ☐ Aislarse en su casa con mayor frecuencia
- ☐ Quedarse en casa con mayor frecuencia
- ☐ Ponerse guantes desechables con mayor frecuencia
- ☐ No sabe

Por cada persona adicional en su hogar, proporcione la siguiente informacion.

Persona 6: ¿Cuál es su relación con esta persona?

- ○ Pareja o cónyuge
- ○ Hijo o hija
- ○ Padre o madre
- ○ Hermano o hermana
- ○ Otro familiar
- ○ Proveedor de cuidado infantil u otros cuidados en casa
- ○ Otra

Persona 6: Especifique su relación con esta persona.

____

Persona 6: ¿Qué edad tiene esta persona?

____
((Especifique la edad en años))

Persona 6: ¿Cuál es el sexo de esta persona?

- ○ Femenino
- ○ Masculino

Persona 6: ¿Cuál es la raza de esta persona?

- ☐ Indio americano o nativo de Alaska
- ☐ Asiático
- ☐ Negro o afroestadounidense
- ☐ Nativo de Hawái o de las islas del Pacífico
- ☐ Blanco
- ☐ Otra
- ☐ No sabe

((Seleccione todo lo que corresponda.))

Persona 6: ¿Cuál es la identidad étnica de esta persona?

- ○ Hispano o latino
- ○ Ni hispano ni latino
- ○ Otra
- ○ No sabe

Persona 6: ¿Cuál es el nivel de educación de esta persona?

- ○ No tiene educación formal
- ○ Kínder a 8° grado
- ○ Estudios de educación secundaria
- ○ Equivalencia de educación secundaria (GED)
- ○ Diploma de educación secundaria
- ○ Estudios de educación superior
- ○ Título universitario
- ○ Estudios de posgrado o más
- ○ No sabe

Persona 6: ¿Cuál de las siguientes es la mejor descripción de la situación laboral actual de esta persona?	◯ Empleo a tiempo completo<br>◯ Empleo a tiempo parcial<br>◯ En busca de empleo<br>◯ Jubilado<br>◯ Ama de casa<br>◯ Estudiante<br>◯ Permiso de maternidad o paternidad<br>◯ Permiso por enfermedad<br>◯ Desempleado por discapacidad<br>◯ Otra<br>◯ No sabe
Persona 6: ¿Se considera esta persona actualmente empleado por cuenta propia (contratista independiente, trabajador esporádico (gig), etc.)?	◯ Sí<br>◯ No<br>◯ No sabe
Persona 6: ¿Trabaja esta persona actualmente en alguno de los siguientes ambientes de alto riesgo de contagio de COVID-19?	☐ Ambiente de asistencia médica (hospital, clínica, centro de urgencias, etc.)<br>☐ Ambiente residencial denso (hogar de ancianos, otro centro de asistencia de larga duración)<br>☐ Prisión o cárcel<br>☐ Establecimiento de envasado de carne<br>☐ Establecimiento de envío o distribución<br>☐ Establecimiento minorista de alto volumen (tienda de provisiones, etc.)<br>☐ No sabe
Persona 6: ¿Le ofrece el empleador a esta persona alguno de los siguientes beneficios en su empleo principal actual?	☐ Permiso por enfermedad con goce de sueldo<br>☐ Vacaciones o permiso personal con goce de sueldo<br>☐ Seguro de salud<br>☐ Seguro de discapacidad<br>☐ Plan de jubilación<br>☐ Otro<br>☐ No sabe<br>((Seleccione todo lo que corresponda.))
Persona 6: En una escala de 0 (definitivamente no va a suceder) a 10 (definitivamente va a suceder), ¿qué tan probable es que esta persona pierda su empleo debido a la pandemia de COVID-19?	__________________________________
Persona 6: En una escala de 0 (definitivamente no va a suceder) a 10 (definitivamente va a suceder), ¿qué tan probable es que a esta persona se le asignen menos horas de trabajo debido a la pandemia de COVID-19?	__________________________________

	Todo el tiempo (100%)	La mayor parte del tiempo (75%)	La mitad del tiempo (50%)	Menos de la mitad del tiempo (25%)	nunca (0%)	Nunca
Persona 6: Actualmente ¿con qué frecuencia se le exige a esta persona que trabaje fuera de su residencia?	◯	◯	◯	◯	◯	◯

Persona 6: Actualmente, ¿con qué frecuencia se encuentra esta persona físicamente cerca de sus compañeros de trabajo mientras trabaja fuera de su residencia?

○ ○ ○ ○ ○ ○

Persona 6: Actualmente, ¿con qué frecuencia se encuentra esta persona físicamente cerca de sus clientes mientras trabaja fuera de su residencia

○ ○ ○ ○ ○ ○

Persona 6: ¿Piensa esta persona vacunarse contra la COVID-19 cuando se ofrezca una vacuna?

○ Sí
○ No
○ No sabe

Persona 6: En las últimas dos semanas, ¿ha tenido esta persona algún síntoma de COVID-19 (tos, fiebre, dificultad para respirar, fatiga, dolores de cuerpo, diarrea, goteo nasal o pérdida del sentido del olfato o del gusto)?

○ Sí
○ No
○ No sabe

Persona 6: ¿Cuándo le comenzaron los síntomas de COVID-19 a esta persona?

____

Persona 6: En vista de sus síntomas, ¿le preocupa a esta persona la posibilidad de tener COVID-19?

○ Sí
○ No
○ No sabe

Persona 6: ¿Fue esta persona objeto de prejuicio o discriminación debido a sus síntomas?

○ Sí
○ No
○ No sabe

Persona 6: ¿Qué hizo esta persona en vista de sus síntomas?

☐ Nada
☐ Tomó medicamentos sin receta (ibuprofeno, acetaminofén, etc.)
☐ Consultó por teléfono a un proveedor de asistencia médica
☐ Fue al consultorio de un proveedor de asistencia médica
☐ Fue a una clínica o una farmacia minorista
☐ Fue a un centro de urgencias (FASTMed, etc.)
☐ Fue a la sala de emergencias
☐ Fue ingresado al hospital
☐ Otra cosa
☐ No sabe
((Seleccione todo lo que corresponda.))

Persona 6: ¿Qué otra cosa hizo esta persona en vista de sus síntomas?

____

Persona 6: ¿Le dijo un proveedor de asistencia médica a esta persona que era posible que tuviera COVID-19?

○ Sí
○ No
○ No sabe

Persona 6: Si a esta persona le hicieron una prueba de COVID-19 en vista de sus síntomas, ¿cuál fue el resultado?	◯ Pendiente<br>◯ Positivo<br>◯ Negativo<br>◯ No concluyente<br>◯ No se hizo la prueba<br>◯ No sabe
Persona 6: ¿Cuántos días estuvo hospitalizada esta persona?	__________________________
Persona 6: ¿Se le hicieron a esta persona las siguientes intervenciones durante su hospitalización?	☐ Oxígeno adicional por la nariz<br>☐ Tratamiento en la Unidad de Cuidados Intensivos (Intensive Care Unit, ICU)<br>☐ Ventilación mecánica (intubación o tubo de respiración)<br>☐ No sabe
Persona 6: ¿Ha vuelto esta persona a su salud normal?	◯ Sí<br>◯ No<br>◯ No sabe
Persona 6: ¿Cuáles de las siguientes medidas tomó esta persona para proteger a sus amigos y familiares después de que comenzaron sus síntomas?	☐ Ponerse mascarilla con mayor frecuencia<br>☐ Lavarse las manos con agua y jabón con mayor frecuencia<br>☐ Limpiarse las manos con desinfectante con mayor frecuencia<br>☐ Aislarse en su casa con mayor frecuencia<br>☐ Quedarse en casa con mayor frecuencia<br>☐ Ponerse guantes desechables con mayor frecuencia<br>☐ No sabe

Por cada persona adicional en su hogar, proporcione la siguiente informacion.

Persona 7: ¿Cuál es su relación con esta persona?	◯ Pareja o cónyuge<br>◯ Hijo o hija<br>◯ Padre o madre<br>◯ Hermano o hermana<br>◯ Otro familiar<br>◯ Proveedor de cuidado infantil u otros cuidados en casa<br>◯ Otra
Persona 7: Especifique su relación con esta persona.	__________________________
Persona 7: ¿Qué edad tiene esta persona?	__________________________<br>((Especifique la edad en años))
Persona 7: ¿Cuál es el sexo de esta persona?	◯ Femenino<br>◯ Masculino

Persona 7: ¿Cuál es la raza de esta persona?	☐ Indio americano o nativo de Alaska<br>☐ Asiático<br>☐ Negro o afroestadounidense<br>☐ Nativo de Hawái o de las islas del Pacífico<br>☐ Blanco<br>☐ Otra<br>☐ No sabe<br>((Seleccione todo lo que corresponda.))
Persona 7: ¿Cuál es la identidad étnica de esta persona?	◯ Hispano o latino<br>◯ Ni hispano ni latino<br>◯ Otra<br>◯ No sabe
Persona 7: ¿Cuál es el nivel de educación de esta persona?	◯ No tiene educación formal<br>◯ Kínder a 8° grado<br>◯ Estudios de educación secundaria<br>◯ Equivalencia de educación secundaria (GED)<br>◯ Diploma de educación secundaria<br>◯ Estudios de educación superior<br>◯ Título universitario<br>◯ Estudios de posgrado o más<br>◯ No sabe
Persona 7: ¿Cuál de las siguientes es la mejor descripción de la situación laboral actual de esta persona?	◯ Empleo a tiempo completo<br>◯ Empleo a tiempo parcial<br>◯ En busca de empleo<br>◯ Jubilado<br>◯ Ama de casa<br>◯ Estudiante<br>◯ Permiso de maternidad o paternidad<br>◯ Permiso por enfermedad<br>◯ Desempleado por discapacidad<br>◯ Otra<br>◯ No sabe
Persona 7: ¿Se considera esta persona actualmente empleado por cuenta propia (contratista independiente, trabajador esporádico (gig), etc.)?	◯ Sí<br>◯ No<br>◯ No sabe
Persona 7: ¿Trabaja esta persona actualmente en alguno de los siguientes ambientes de alto riesgo de contagio de COVID-19?	☐ Ambiente de asistencia médica (hospital, clínica, centro de urgencias, etc.)<br>☐ Ambiente residencial denso (hogar de ancianos, otro centro de asistencia de larga duración)<br>☐ Prisión o cárcel<br>☐ Establecimiento de envasado de carne<br>☐ Establecimiento de envío o distribución<br>☐ Establecimiento minorista de alto volumen (tienda de provisiones, etc.)<br>☐ No sabe
Persona 7: ¿Le ofrece el empleador a esta persona alguno de los siguientes beneficios en su empleo principal actual?	☐ Permiso por enfermedad con goce de sueldo<br>☐ Vacaciones o permiso personal con goce de sueldo<br>☐ Seguro de salud<br>☐ Seguro de discapacidad<br>☐ Plan de jubilación<br>☐ Otro<br>☐ No sabe<br>((Seleccione todo lo que corresponda.))

Persona 7: En una escala de 0 (definitivamente no va a suceder) a 10 (definitivamente va a suceder), ¿qué tan probable es que esta persona pierda su empleo debido a la pandemia de COVID-19?

____

Persona 7: En una escala de 0 (definitivamente no va a suceder) a 10 (definitivamente va a suceder), ¿qué tan probable es que a esta persona se le asignen menos horas de trabajo debido a la pandemia de COVID-19?

____

	Todo el tiempo (100%)	La mayor parte del tiempo (75%)	La mitad del tiempo (50%)	Menos de la mitad del tiempo (25%)	nunca (0%)	Nunca
Persona 7: Actualmente ¿con qué frecuencia se le exige a esta persona que trabaje fuera de su residencia?	◯	◯	◯	◯	◯	◯
Persona 7: Actualmente, ¿con qué frecuencia se encuentra esta persona físicamente cerca de sus compañeros de trabajo mientras trabaja fuera de su residencia?	◯	◯	◯	◯	◯	◯
Persona 7: Actualmente, ¿con qué frecuencia se encuentra esta persona físicamente cerca de sus clientes mientras trabaja fuera de su residencia	◯	◯	◯	◯	◯	◯

Persona 7: ¿Piensa esta persona vacunarse contra la COVID-19 cuando se ofrezca una vacuna?

◯ Sí
◯ No
◯ No sabe

Persona 7: En las últimas dos semanas, ¿ha tenido esta persona algún síntoma de COVID-19 (tos, fiebre, dificultad para respirar, fatiga, dolores de cuerpo, diarrea, goteo nasal o pérdida del sentido del olfato o del gusto)?

◯ Sí
◯ No
◯ No sabe

Persona 7: ¿Cuándo le comenzaron los síntomas de COVID-19 a esta persona?

____

Persona 7: En vista de sus síntomas, ¿le preocupa a esta persona la posibilidad de tener COVID-19?

◯ Sí
◯ No
◯ No sabe

Persona 7: ¿Fue esta persona objeto de prejuicio o discriminación debido a sus síntomas?

◯ Sí
◯ No
◯ No sabe

Persona 7: ¿Qué hizo esta persona en vista de sus síntomas?	☐ Nada<br>☐ Tomó medicamentos sin receta (ibuprofeno, acetaminofén, etc.)<br>☐ Consultó por teléfono a un proveedor de asistencia médica<br>☐ Fue al consultorio de un proveedor de asistencia médica<br>☐ Fue a una clínica o una farmacia minorista<br>☐ Fue a un centro de urgencias (FASTMed, etc.)<br>☐ Fue a la sala de emergencias<br>☐ Fue ingresado al hospital<br>☐ Otra cosa<br>☐ No sabe<br>((Seleccione todo lo que corresponda.))
Persona 7: ¿Qué otra cosa hizo esta persona en vista de sus síntomas?	__________________________________
Persona 7: ¿Le dijo un proveedor de asistencia médica a esta persona que era posible que tuviera COVID-19?	◯ Sí<br>◯ No<br>◯ No sabe
Persona 7: Si a esta persona le hicieron una prueba de COVID-19 en vista de sus síntomas, ¿cuál fue el resultado?	◯ Pendiente<br>◯ Positivo<br>◯ Negativo<br>◯ No concluyente<br>◯ No se hizo la prueba<br>◯ No sabe
Persona 7: ¿Cuántos días estuvo hospitalizada esta persona?	__________________________________
Persona 7: ¿Se le hicieron a esta persona las siguientes intervenciones durante su hospitalización?	☐ Oxígeno adicional por la nariz<br>☐ Tratamiento en la Unidad de Cuidados Intensivos (Intensive Care Unit, ICU)<br>☐ Ventilación mecánica (intubación o tubo de respiración)<br>☐ No sabe
Persona 7: ¿Ha vuelto esta persona a su salud normal?	◯ Sí<br>◯ No<br>◯ No sabe
Persona 7: ¿Cuáles de las siguientes medidas tomó esta persona para proteger a sus amigos y familiares después de que comenzaron sus síntomas?	☐ Ponerse mascarilla con mayor frecuencia<br>☐ Lavarse las manos con agua y jabón con mayor frecuencia<br>☐ Limpiarse las manos con desinfectante con mayor frecuencia<br>☐ Aislarse en su casa con mayor frecuencia<br>☐ Quedarse en casa con mayor frecuencia<br>☐ Ponerse guantes desechables con mayor frecuencia<br>☐ No sabe

Por cada persona adicional en su hogar, proporcione la siguiente informacion.

Persona 8: ¿Cuál es su relación con esta persona?	◯ Pareja o cónyuge<br>◯ Hijo o hija<br>◯ Padre o madre<br>◯ Hermano o hermana<br>◯ Otro familiar<br>◯ Proveedor de cuidado infantil u otros cuidados en casa<br>◯ Otra
Persona 8: Especifique su relación con esta persona.	______________________________
Persona 8: ¿Qué edad tiene esta persona?	______________________________<br>((Especifique la edad en años))
Persona 8: ¿Cuál es el sexo de esta persona?	◯ Femenino<br>◯ Masculino
Persona 8: ¿Cuál es la raza de esta persona?	☐ Indio americano o nativo de Alaska<br>☐ Asiático<br>☐ Negro o afroestadounidense<br>☐ Nativo de Hawái o de las islas del Pacífico<br>☐ Blanco<br>☐ Otra<br>☐ No sabe<br>((Seleccione todo lo que corresponda.))
Persona 8: ¿Cuál es la identidad étnica de esta persona?	◯ Hispano o latino<br>◯ Ni hispano ni latino<br>◯ Otra<br>◯ No sabe
Persona 8: ¿Cuál es el nivel de educación de esta persona?	◯ No tiene educación formal<br>◯ Kínder a 8° grado<br>◯ Estudios de educación secundaria<br>◯ Equivalencia de educación secundaria (GED)<br>◯ Diploma de educación secundaria<br>◯ Estudios de educación superior<br>◯ Título universitario<br>◯ Estudios de posgrado o más<br>◯ No sabe
Persona 8: ¿Cuál de las siguientes es la mejor descripción de la situación laboral actual de esta persona?	◯ Empleo a tiempo completo<br>◯ Empleo a tiempo parcial<br>◯ En busca de empleo<br>◯ Jubilado<br>◯ Ama de casa<br>◯ Estudiante<br>◯ Permiso de maternidad o paternidad<br>◯ Permiso por enfermedad<br>◯ Desempleado por discapacidad<br>◯ Otra<br>◯ No sabe
Persona 8: ¿Se considera esta persona actualmente empleado por cuenta propia (contratista independiente, trabajador esporádico (gig), etc.)?	◯ Sí<br>◯ No<br>◯ No sabe

Persona 8: ¿Trabaja esta persona actualmente en alguno de los siguientes ambientes de alto riesgo de contagio de COVID-19?

- ☐ Ambiente de asistencia médica (hospital, clínica, centro de urgencias, etc.)
- ☐ Ambiente residencial denso (hogar de ancianos, otro centro de asistencia de larga duración)
- ☐ Prisión o cárcel
- ☐ Establecimiento de envasado de carne
- ☐ Establecimiento de envío o distribución
- ☐ Establecimiento minorista de alto volumen (tienda de provisiones, etc.)
- ☐ No sabe

Persona 8: ¿Le ofrece el empleador a esta persona alguno de los siguientes beneficios en su empleo principal actual?

- ☐ Permiso por enfermedad con goce de sueldo
- ☐ Vacaciones o permiso personal con goce de sueldo
- ☐ Seguro de salud
- ☐ Seguro de discapacidad
- ☐ Plan de jubilación
- ☐ Otro
- ☐ No sabe

((Seleccione todo lo que corresponda.))

Persona 8: En una escala de 0 (definitivamente no va a suceder) a 10 (definitivamente va a suceder), ¿qué tan probable es que esta persona pierda su empleo debido a la pandemia de COVID-19?

____

Persona 8: En una escala de 0 (definitivamente no va a suceder) a 10 (definitivamente va a suceder), ¿qué tan probable es que a esta persona se le asignen menos horas de trabajo debido a la pandemia de COVID-19?

____

	Todo el tiempo (100%)	La mayor parte del tiempo (75%)	La mitad del tiempo (50%)	Menos de la mitad del tiempo (25%)	nunca (0%)	Nunca
Persona 8: Actualmente ¿con qué frecuencia se le exige a esta persona que trabaje fuera de su residencia?	○	○	○	○	○	○
Persona 8: Actualmente, ¿con qué frecuencia se encuentra esta persona físicamente cerca de sus compañeros de trabajo mientras trabaja fuera de su residencia?	○	○	○	○	○	○
Persona 8: Actualmente, ¿con qué frecuencia se encuentra esta persona físicamente cerca de sus clientes mientras trabaja fuera de su residencia	○	○	○	○	○	○

Persona 8: ¿Piensa esta persona vacunarse contra la COVID-19 cuando se ofrezca una vacuna?

- ○ Sí
- ○ No
- ○ No sabe

Persona 8: En las últimas dos semanas, ¿ha tenido esta persona algún síntoma de COVID-19 (tos, fiebre, dificultad para respirar, fatiga, dolores de cuerpo, diarrea, goteo nasal o pérdida del sentido del olfato o del gusto)?	◯ Sí<br>◯ No<br>◯ No sabe
Persona 8: ¿Cuándo le comenzaron los síntomas de COVID-19 a esta persona?	__________________________________
Persona 8: En vista de sus síntomas, ¿le preocupa a esta persona la posibilidad de tener COVID-19?	◯ Sí<br>◯ No<br>◯ No sabe
Persona 8: ¿Fue esta persona objeto de prejuicio o discriminación debido a sus síntomas?	◯ Sí<br>◯ No<br>◯ No sabe
Persona 8: ¿Qué hizo esta persona en vista de sus síntomas?	☐ Nada<br>☐ Tomó medicamentos sin receta (ibuprofeno, acetaminofén, etc.)<br>☐ Consultó por teléfono a un proveedor de asistencia médica<br>☐ Fue al consultorio de un proveedor de asistencia médica<br>☐ Fue a una clínica o una farmacia minorista<br>☐ Fue a un centro de urgencias (FASTMed, etc.)<br>☐ Fue a la sala de emergencias<br>☐ Fue ingresado al hospital<br>☐ Otra cosa<br>☐ No sabe<br>((Seleccione todo lo que corresponda.))
Persona 8: ¿Qué otra cosa hizo esta persona en vista de sus síntomas?	__________________________________
Persona 8: ¿Le dijo un proveedor de asistencia médica a esta persona que era posible que tuviera COVID-19?	◯ Sí<br>◯ No<br>◯ No sabe
Persona 8: Si a esta persona le hicieron una prueba de COVID-19 en vista de sus síntomas, ¿cuál fue el resultado?	◯ Pendiente<br>◯ Positivo<br>◯ Negativo<br>◯ No concluyente<br>◯ No se hizo la prueba<br>◯ No sabe
Persona 8: ¿Cuántos días estuvo hospitalizada esta persona?	__________________________________
Persona 8: ¿Se le hicieron a esta persona las siguientes intervenciones durante su hospitalización?	☐ Oxígeno adicional por la nariz<br>☐ Tratamiento en la Unidad de Cuidados Intensivos (Intensive Care Unit, ICU)<br>☐ Ventilación mecánica (intubación o tubo de respiración)<br>☐ No sabe
Persona 8: ¿Ha vuelto esta persona a su salud normal?	◯ Sí<br>◯ No<br>◯ No sabe

Persona 8: ¿Cuáles de las siguientes medidas tomó esta persona para proteger a sus amigos y familiares después de que comenzaron sus síntomas?

- ☐ Ponerse mascarilla con mayor frecuencia
- ☐ Lavarse las manos con agua y jabón con mayor frecuencia
- ☐ Limpiarse las manos con desinfectante con mayor frecuencia
- ☐ Aislarse en su casa con mayor frecuencia
- ☐ Quedarse en casa con mayor frecuencia
- ☐ Ponerse guantes desechables con mayor frecuencia
- ☐ No sabe

Por cada persona adicional en su hogar, proporcione la siguiente informacion.

Persona 9: ¿Cuál es su relación con esta persona?

- ◯ Pareja o cónyuge
- ◯ Hijo o hija
- ◯ Padre o madre
- ◯ Hermano o hermana
- ◯ Otro familiar
- ◯ Proveedor de cuidado infantil u otros cuidados en casa
- ◯ Otra

Persona 9: Especifique su relación con esta persona.

____

Persona 9: ¿Qué edad tiene esta persona?

____
((Especifique la edad en años))

Persona 9: ¿Cuál es el sexo de esta persona?

- ◯ Femenino
- ◯ Masculino

Persona 9: ¿Cuál es la raza de esta persona?

- ☐ Indio americano o nativo de Alaska
- ☐ Asiático
- ☐ Negro o afroestadounidense
- ☐ Nativo de Hawái o de las islas del Pacífico
- ☐ Blanco
- ☐ Otra
- ☐ No sabe

((Seleccione todo lo que corresponda.))

Persona 9: ¿Cuál es la identidad étnica de esta persona?

- ◯ Hispano o latino
- ◯ Ni hispano ni latino
- ◯ Otra
- ◯ No sabe

Persona 9: ¿Cuál es el nivel de educación de esta persona?

- ◯ No tiene educación formal
- ◯ Kínder a 8° grado
- ◯ Estudios de educación secundaria
- ◯ Equivalencia de educación secundaria (GED)
- ◯ Diploma de educación secundaria
- ◯ Estudios de educación superior
- ◯ Título universitario
- ◯ Estudios de posgrado o más
- ◯ No sabe

Persona 9: ¿Cuál de las siguientes es la mejor descripción de la situación laboral actual de esta persona?	◯ Empleo a tiempo completo<br>◯ Empleo a tiempo parcial<br>◯ En busca de empleo<br>◯ Jubilado<br>◯ Ama de casa<br>◯ Estudiante<br>◯ Permiso de maternidad o paternidad<br>◯ Permiso por enfermedad<br>◯ Desempleado por discapacidad<br>◯ Otra<br>◯ No sabe
Persona 9: ¿Se considera esta persona actualmente empleado por cuenta propia (contratista independiente, trabajador esporádico (gig), etc.)?	◯ Sí<br>◯ No<br>◯ No sabe
Persona 9: ¿Trabaja esta persona actualmente en alguno de los siguientes ambientes de alto riesgo de contagio de COVID-19?	☐ Ambiente de asistencia médica (hospital, clínica, centro de urgencias, etc.)<br>☐ Ambiente residencial denso (hogar de ancianos, otro centro de asistencia de larga duración)<br>☐ Prisión o cárcel<br>☐ Establecimiento de envasado de carne<br>☐ Establecimiento de envío o distribución<br>☐ Establecimiento minorista de alto volumen (tienda de provisiones, etc.)<br>☐ No sabe
Persona 9: ¿Le ofrece el empleador a esta persona alguno de los siguientes beneficios en su empleo principal actual?	☐ Permiso por enfermedad con goce de sueldo<br>☐ Vacaciones o permiso personal con goce de sueldo<br>☐ Seguro de salud<br>☐ Seguro de discapacidad<br>☐ Plan de jubilación<br>☐ Otro<br>☐ No sabe<br>((Seleccione todo lo que corresponda.))
Persona 9: En una escala de 0 (definitivamente no va a suceder) a 10 (definitivamente va a suceder), ¿qué tan probable es que esta persona pierda su empleo debido a la pandemia de COVID-19?	__________________________________
Persona 9: En una escala de 0 (definitivamente no va a suceder) a 10 (definitivamente va a suceder), ¿qué tan probable es que a esta persona se le asignen menos horas de trabajo debido a la pandemia de COVID-19?	__________________________________

	Todo el tiempo (100%)	La mayor parte del tiempo (75%)	La mitad del tiempo (50%)	Menos de la mitad del tiempo (25%)	nunca (0%)	Nunca
Persona 9: Actualmente ¿con qué frecuencia se le exige a esta persona que trabaje fuera de su residencia?	◯	◯	◯	◯	◯	◯

Persona 9: Actualmente, ¿con qué frecuencia se encuentra esta persona físicamente cerca de sus compañeros de trabajo mientras trabaja fuera de su residencia?	◯	◯	◯	◯	◯	◯
Persona 9: Actualmente, ¿con qué frecuencia se encuentra esta persona físicamente cerca de sus clientes mientras trabaja fuera de su residencia	◯	◯	◯	◯	◯	◯

Persona 9: ¿Piensa esta persona vacunarse contra la COVID-19 cuando se ofrezca una vacuna?	◯ Sí<br>◯ No<br>◯ No sabe
Persona 9: En las últimas dos semanas, ¿ha tenido esta persona algún síntoma de COVID-19 (tos, fiebre, dificultad para respirar, fatiga, dolores de cuerpo, diarrea, goteo nasal o pérdida del sentido del olfato o del gusto)?	◯ Sí<br>◯ No<br>◯ No sabe
Persona 9: ¿Cuándo le comenzaron los síntomas de COVID-19 a esta persona?	______________________________
Persona 9: En vista de sus síntomas, ¿le preocupa a esta persona la posibilidad de tener COVID-19?	◯ Sí<br>◯ No<br>◯ No sabe
Persona 9: ¿Fue esta persona objeto de prejuicio o discriminación debido a sus síntomas?	◯ Sí<br>◯ No<br>◯ No sabe
Persona 9: ¿Qué hizo esta persona en vista de sus síntomas?	☐ Nada<br>☐ Tomó medicamentos sin receta (ibuprofeno, acetaminofén, etc.)<br>☐ Consultó por teléfono a un proveedor de asistencia médica<br>☐ Fue al consultorio de un proveedor de asistencia médica<br>☐ Fue a una clínica o una farmacia minorista<br>☐ Fue a un centro de urgencias (FASTMed, etc.)<br>☐ Fue a la sala de emergencias<br>☐ Fue ingresado al hospital<br>☐ Otra cosa<br>☐ No sabe<br>((Seleccione todo lo que corresponda.))
Persona 9: ¿Qué otra cosa hizo esta persona en vista de sus síntomas?	______________________________
Persona 9: ¿Le dijo un proveedor de asistencia médica a esta persona que era posible que tuviera COVID-19?	◯ Sí<br>◯ No<br>◯ No sabe

Persona 9: Si a esta persona le hicieron una prueba de COVID-19 en vista de sus síntomas, ¿cuál fue el resultado?	◯ Pendiente<br>◯ Positivo<br>◯ Negativo<br>◯ No concluyente<br>◯ No se hizo la prueba<br>◯ No sabe
Persona 9: ¿Cuántos días estuvo hospitalizada esta persona?	__________________________
Persona 9: ¿Se le hicieron a esta persona las siguientes intervenciones durante su hospitalización?	☐ Oxígeno adicional por la nariz<br>☐ Tratamiento en la Unidad de Cuidados Intensivos (Intensive Care Unit, ICU)<br>☐ Ventilación mecánica (intubación o tubo de respiración)<br>☐ No sabe
Persona 9: ¿Ha vuelto esta persona a su salud normal?	◯ Sí<br>◯ No<br>◯ No sabe
Persona 9: ¿Cuáles de las siguientes medidas tomó esta persona para proteger a sus amigos y familiares después de que comenzaron sus síntomas?	☐ Ponerse mascarilla con mayor frecuencia<br>☐ Lavarse las manos con agua y jabón con mayor frecuencia<br>☐ Limpiarse las manos con desinfectante con mayor frecuencia<br>☐ Aislarse en su casa con mayor frecuencia<br>☐ Quedarse en casa con mayor frecuencia<br>☐ Ponerse guantes desechables con mayor frecuencia<br>☐ No sabe

Por cada persona adicional en su hogar, proporcione la siguiente informacion.

Persona 10: ¿Cuál es su relación con esta persona?	◯ Pareja o cónyuge<br>◯ Hijo o hija<br>◯ Padre o madre<br>◯ Hermano o hermana<br>◯ Otro familiar<br>◯ Proveedor de cuidado infantil u otros cuidados en casa<br>◯ Otra
Persona 10: Especifique su relación con esta persona.	__________________________
Persona 10: ¿Qué edad tiene esta persona?	__________________________<br>((Especifique la edad en años))
Persona 10: ¿Cuál es el sexo de esta persona?	◯ Femenino<br>◯ Masculino

Persona 10: ¿Cuál es la raza de esta persona?	☐ Indio americano o nativo de Alaska<br>☐ Asiático<br>☐ Negro o afroestadounidense<br>☐ Nativo de Hawái o de las islas del Pacífico<br>☐ Blanco<br>☐ Otra<br>☐ No sabe<br>((Seleccione todo lo que corresponda.))
Persona 10: ¿Cuál es la identidad étnica de esta persona?	◯ Hispano o latino<br>◯ Ni hispano ni latino<br>◯ Otra<br>◯ No sabe
Persona 10: ¿Cuál es el nivel de educación de esta persona?	◯ No tiene educación formal<br>◯ Kínder a 8° grado<br>◯ Estudios de educación secundaria<br>◯ Equivalencia de educación secundaria (GED)<br>◯ Diploma de educación secundaria<br>◯ Estudios de educación superior<br>◯ Título universitario<br>◯ Estudios de posgrado o más<br>◯ No sabe
Persona 10: ¿Cuál de las siguientes es la mejor descripción de la situación laboral actual de esta persona?	◯ Empleo a tiempo completo<br>◯ Empleo a tiempo parcial<br>◯ En busca de empleo<br>◯ Jubilado<br>◯ Ama de casa<br>◯ Estudiante<br>◯ Permiso de maternidad o paternidad<br>◯ Permiso por enfermedad<br>◯ Desempleado por discapacidad<br>◯ Otra<br>◯ No sabe
Persona 10: ¿Se considera esta persona actualmente empleado por cuenta propia (contratista independiente, trabajador esporádico (gig), etc.)?	◯ Sí<br>◯ No<br>◯ No sabe
Persona 10: ¿Trabaja esta persona actualmente en alguno de los siguientes ambientes de alto riesgo de contagio de COVID-19?	☐ Ambiente de asistencia médica (hospital, clínica, centro de urgencias, etc.)<br>☐ Ambiente residencial denso (hogar de ancianos, otro centro de asistencia de larga duración)<br>☐ Prisión o cárcel<br>☐ Establecimiento de envasado de carne<br>☐ Establecimiento de envío o distribución<br>☐ Establecimiento minorista de alto volumen (tienda de provisiones, etc.)<br>☐ No sabe
Persona 10: ¿Le ofrece el empleador a esta persona alguno de los siguientes beneficios en su empleo principal actual?	☐ Permiso por enfermedad con goce de sueldo<br>☐ Vacaciones o permiso personal con goce de sueldo<br>☐ Seguro de salud<br>☐ Seguro de discapacidad<br>☐ Plan de jubilación<br>☐ Otro<br>☐ No sabe<br>((Seleccione todo lo que corresponda.))

Persona 10: En una escala de 0 (definitivamente no va a suceder) a 10 (definitivamente va a suceder), ¿qué tan probable es que esta persona pierda su empleo debido a la pandemia de COVID-19?

____

Persona 10: En una escala de 0 (definitivamente no va a suceder) a 10 (definitivamente va a suceder), ¿qué tan probable es que a esta persona se le asignen menos horas de trabajo debido a la pandemia de COVID-19?

____

	Todo el tiempo (100%)	La mayor parte del tiempo (75%)	La mitad del tiempo (50%)	Menos de la mitad del tiempo (25%)	nunca (0%)	Nunca
Persona 10: Actualmente ¿con qué frecuencia se le exige a esta persona que trabaje fuera de su residencia?	◯	◯	◯	◯	◯	◯
Persona 10: Actualmente, ¿con qué frecuencia se encuentra esta persona físicamente cerca de sus compañeros de trabajo mientras trabaja fuera de su residencia?	◯	◯	◯	◯	◯	◯
Persona 10: Actualmente, ¿con qué frecuencia se encuentra esta persona físicamente cerca de sus clientes mientras trabaja fuera de su residencia	◯	◯	◯	◯	◯	◯

Persona 10: ¿Piensa esta persona vacunarse contra la COVID-19 cuando se ofrezca una vacuna?

◯ Sí
◯ No
◯ No sabe

Persona 10: En las últimas dos semanas, ¿ha tenido esta persona algún síntoma de COVID-19 (tos, fiebre, dificultad para respirar, fatiga, dolores de cuerpo, diarrea, goteo nasal o pérdida del sentido del olfato o del gusto)?

◯ Sí
◯ No
◯ No sabe

Persona 10: ¿Cuándo le comenzaron los síntomas de COVID-19 a esta persona?

____

Persona 10: En vista de sus síntomas, ¿le preocupa a esta persona la posibilidad de tener COVID-19?

◯ Sí
◯ No
◯ No sabe

Persona 10: ¿Fue esta persona objeto de prejuicio o discriminación debido a sus síntomas?

◯ Sí
◯ No
◯ No sabe

Persona 10: ¿Qué hizo esta persona en vista de sus síntomas?	☐ Nada<br>☐ Tomó medicamentos sin receta (ibuprofeno, acetaminofén, etc.)<br>☐ Consultó por teléfono a un proveedor de asistencia médica<br>☐ Fue al consultorio de un proveedor de asistencia médica<br>☐ Fue a una clínica o una farmacia minorista<br>☐ Fue a un centro de urgencias (FASTMed, etc.)<br>☐ Fue a la sala de emergencias<br>☐ Fue ingresado al hospital<br>☐ Otra cosa<br>☐ No sabe<br>((Seleccione todo lo que corresponda.))
Persona 10: ¿Qué otra cosa hizo esta persona en vista de sus síntomas?	__________________________________
Persona 10: ¿Le dijo un proveedor de asistencia médica a esta persona que era posible que tuviera COVID-19?	◯ Sí<br>◯ No<br>◯ No sabe
Persona 10: Si a esta persona le hicieron una prueba de COVID-19 en vista de sus síntomas, ¿cuál fue el resultado?	◯ Pendiente<br>◯ Positivo<br>◯ Negativo<br>◯ No concluyente<br>◯ No se hizo la prueba<br>◯ No sabe
Persona 10: ¿Cuántos días estuvo hospitalizada esta persona?	__________________________________
Persona 10: ¿Se le hicieron a esta persona las siguientes intervenciones durante su hospitalización?	☐ Oxígeno adicional por la nariz<br>☐ Tratamiento en la Unidad de Cuidados Intensivos (Intensive Care Unit, ICU)<br>☐ Ventilación mecánica (intubación o tubo de respiración)<br>☐ No sabe
Persona 10: ¿Ha vuelto esta persona a su salud normal?	◯ Sí<br>◯ No<br>◯ No sabe
Persona 10: ¿Cuáles de las siguientes medidas tomó esta persona para proteger a sus amigos y familiares después de que comenzaron sus síntomas?	☐ Ponerse mascarilla con mayor frecuencia<br>☐ Lavarse las manos con agua y jabón con mayor frecuencia<br>☐ Limpiarse las manos con desinfectante con mayor frecuencia<br>☐ Aislarse en su casa con mayor frecuencia<br>☐ Quedarse en casa con mayor frecuencia<br>☐ Ponerse guantes desechables con mayor frecuencia<br>☐ No sabe

Por cada persona adicional en su hogar, proporcione la siguiente informacion.

Persona 11: ¿Cuál es su relación con esta persona?	◯ Pareja o cónyuge<br>◯ Hijo o hija<br>◯ Padre o madre<br>◯ Hermano o hermana<br>◯ Otro familiar<br>◯ Proveedor de cuidado infantil u otros cuidados en casa<br>◯ Otra
Persona 11: Especifique su relación con esta persona.	__________________________________
Persona 11: ¿Qué edad tiene esta persona?	__________________________________<br>((Especifique la edad en años))
Persona 11: ¿Cuál es el sexo de esta persona?	◯ Femenino<br>◯ Masculino
Persona 11: ¿Cuál es la raza de esta persona?	☐ Indio americano o nativo de Alaska<br>☐ Asiático<br>☐ Negro o afroestadounidense<br>☐ Nativo de Hawái o de las islas del Pacífico<br>☐ Blanco<br>☐ Otra<br>☐ No sabe<br>((Seleccione todo lo que corresponda.))
Persona 11: ¿Cuál es la identidad étnica de esta persona?	◯ Hispano o latino<br>◯ Ni hispano ni latino<br>◯ Otra<br>◯ No sabe
Persona 11: ¿Cuál es el nivel de educación de esta persona?	◯ No tiene educación formal<br>◯ Kínder a 8° grado<br>◯ Estudios de educación secundaria<br>◯ Equivalencia de educación secundaria (GED)<br>◯ Diploma de educación secundaria<br>◯ Estudios de educación superior<br>◯ Título universitario<br>◯ Estudios de posgrado o más<br>◯ No sabe
Persona 11: ¿Cuál de las siguientes es la mejor descripción de la situación laboral actual de esta persona?	◯ Empleo a tiempo completo<br>◯ Empleo a tiempo parcial<br>◯ En busca de empleo<br>◯ Jubilado<br>◯ Ama de casa<br>◯ Estudiante<br>◯ Permiso de maternidad o paternidad<br>◯ Permiso por enfermedad<br>◯ Desempleado por discapacidad<br>◯ Otra<br>◯ No sabe
Persona 11: ¿Se considera esta persona actualmente empleado por cuenta propia (contratista independiente, trabajador esporádico (gig), etc.)?	◯ Sí<br>◯ No<br>◯ No sabe

Persona 11: ¿Trabaja esta persona actualmente en alguno de los siguientes ambientes de alto riesgo de contagio de COVID-19?

- ☐ Ambiente de asistencia médica (hospital, clínica, centro de urgencias, etc.)
- ☐ Ambiente residencial denso (hogar de ancianos, otro centro de asistencia de larga duración)
- ☐ Prisión o cárcel
- ☐ Establecimiento de envasado de carne
- ☐ Establecimiento de envío o distribución
- ☐ Establecimiento minorista de alto volumen (tienda de provisiones, etc.)
- ☐ No sabe

Persona 11: ¿Le ofrece el empleador a esta persona alguno de los siguientes beneficios en su empleo principal actual?

- ☐ Permiso por enfermedad con goce de sueldo
- ☐ Vacaciones o permiso personal con goce de sueldo
- ☐ Seguro de salud
- ☐ Seguro de discapacidad
- ☐ Plan de jubilación
- ☐ Otro
- ☐ No sabe

((Seleccione todo lo que corresponda.))

Persona 11: En una escala de 0 (definitivamente no va a suceder) a 10 (definitivamente va a suceder), ¿qué tan probable es que esta persona pierda su empleo debido a la pandemia de COVID-19?

____

Persona 11: En una escala de 0 (definitivamente no va a suceder) a 10 (definitivamente va a suceder), ¿qué tan probable es que a esta persona se le asignen menos horas de trabajo debido a la pandemia de COVID-19?

____

	Todo el tiempo (100%)	La mayor parte del tiempo (75%)	La mitad del tiempo (50%)	Menos de la mitad del tiempo (25%)	nunca (0%)	Nunca
Persona 11: Actualmente ¿con qué frecuencia se le exige a esta persona que trabaje fuera de su residencia?	○	○	○	○	○	○
Persona 11: Actualmente, ¿con qué frecuencia se encuentra esta persona físicamente cerca de sus compañeros de trabajo mientras trabaja fuera de su residencia?	○	○	○	○	○	○
Persona 11: Actualmente, ¿con qué frecuencia se encuentra esta persona físicamente cerca de sus clientes mientras trabaja fuera de su residencia	○	○	○	○	○	○

Persona 11: ¿Piensa esta persona vacunarse contra la COVID-19 cuando se ofrezca una vacuna?

- ○ Sí
- ○ No
- ○ No sabe

Persona 11: En las últimas dos semanas, ¿ha tenido esta persona algún síntoma de COVID-19 (tos, fiebre, dificultad para respirar, fatiga, dolores de cuerpo, diarrea, goteo nasal o pérdida del sentido del olfato o del gusto)?	◯ Sí<br>◯ No<br>◯ No sabe
Persona 11: ¿Cuándo le comenzaron los síntomas de COVID-19 a esta persona?	______________________________
Persona 11: En vista de sus síntomas, ¿le preocupa a esta persona la posibilidad de tener COVID-19?	◯ Sí<br>◯ No<br>◯ No sabe
Persona 11: ¿Fue esta persona objeto de prejuicio o discriminación debido a sus síntomas?	◯ Sí<br>◯ No<br>◯ No sabe
Persona 11: ¿Qué hizo esta persona en vista de sus síntomas?	☐ Nada<br>☐ Tomó medicamentos sin receta (ibuprofeno, acetaminofén, etc.)<br>☐ Consultó por teléfono a un proveedor de asistencia médica<br>☐ Fue al consultorio de un proveedor de asistencia médica<br>☐ Fue a una clínica o una farmacia minorista<br>☐ Fue a un centro de urgencias (FASTMed, etc.)<br>☐ Fue a la sala de emergencias<br>☐ Fue ingresado al hospital<br>☐ Otra cosa<br>☐ No sabe<br>((Seleccione todo lo que corresponda.))
Persona 11: ¿Qué otra cosa hizo esta persona en vista de sus síntomas?	______________________________
Persona 11: ¿Le dijo un proveedor de asistencia médica a esta persona que era posible que tuviera COVID-19?	◯ Sí<br>◯ No<br>◯ No sabe
Persona 11: Si a esta persona le hicieron una prueba de COVID-19 en vista de sus síntomas, ¿cuál fue el resultado?	◯ Pendiente<br>◯ Positivo<br>◯ Negativo<br>◯ No concluyente<br>◯ No se hizo la prueba<br>◯ No sabe
Persona 11: ¿Cuántos días estuvo hospitalizada esta persona?	______________________________
Persona 11: ¿Se le hicieron a esta persona las siguientes intervenciones durante su hospitalización?	☐ Oxígeno adicional por la nariz<br>☐ Tratamiento en la Unidad de Cuidados Intensivos (Intensive Care Unit, ICU)<br>☐ Ventilación mecánica (intubación o tubo de respiración)<br>☐ No sabe
Persona 11: ¿Ha vuelto esta persona a su salud normal?	◯ Sí<br>◯ No<br>◯ No sabe

Persona 11: ¿Cuáles de las siguientes medidas tomó esta persona para proteger a sus amigos y familiares después de que comenzaron sus síntomas?

- ☐ Ponerse mascarilla con mayor frecuencia
- ☐ Lavarse las manos con agua y jabón con mayor frecuencia
- ☐ Limpiarse las manos con desinfectante con mayor frecuencia
- ☐ Aislarse en su casa con mayor frecuencia
- ☐ Quedarse en casa con mayor frecuencia
- ☐ Ponerse guantes desechables con mayor frecuencia
- ☐ No sabe

Por cada persona adicional en su hogar, proporcione la siguiente informacion.

Persona 12: ¿Cuál es su relación con esta persona?

- ◯ Pareja o cónyuge
- ◯ Hijo o hija
- ◯ Padre o madre
- ◯ Hermano o hermana
- ◯ Otro familiar
- ◯ Proveedor de cuidado infantil u otros cuidados en casa
- ◯ Otra

Persona 12: Especifique su relación con esta persona.

____

Persona 12: ¿Qué edad tiene esta persona?

____
((Especifique la edad en años))

Persona 12: ¿Cuál es el sexo de esta persona?

- ◯ Femenino
- ◯ Masculino

Persona 12: ¿Cuál es la raza de esta persona?

- ☐ Indio americano o nativo de Alaska
- ☐ Asiático
- ☐ Negro o afroestadounidense
- ☐ Nativo de Hawái o de las islas del Pacífico
- ☐ Blanco
- ☐ Otra
- ☐ No sabe

((Seleccione todo lo que corresponda.))

Persona 12: ¿Cuál es la identidad étnica de esta persona?

- ◯ Hispano o latino
- ◯ Ni hispano ni latino
- ◯ Otra
- ◯ No sabe

Persona 12: ¿Cuál es el nivel de educación de esta persona?

- ◯ No tiene educación formal
- ◯ Kínder a 8° grado
- ◯ Estudios de educación secundaria
- ◯ Equivalencia de educación secundaria (GED)
- ◯ Diploma de educación secundaria
- ◯ Estudios de educación superior
- ◯ Título universitario
- ◯ Estudios de posgrado o más
- ◯ No sabe

Persona 12: ¿Cuál de las siguientes es la mejor descripción de la situación laboral actual de esta persona?	◯ Empleo a tiempo completo<br>◯ Empleo a tiempo parcial<br>◯ En busca de empleo<br>◯ Jubilado<br>◯ Ama de casa<br>◯ Estudiante<br>◯ Permiso de maternidad o paternidad<br>◯ Permiso por enfermedad<br>◯ Desempleado por discapacidad<br>◯ Otra<br>◯ No sabe
Persona 12: ¿Se considera esta persona actualmente empleado por cuenta propia (contratista independiente, trabajador esporádico (gig), etc.)?	◯ Sí<br>◯ No<br>◯ No sabe
Persona 12: ¿Trabaja esta persona actualmente en alguno de los siguientes ambientes de alto riesgo de contagio de COVID-19?	☐ Ambiente de asistencia médica (hospital, clínica, centro de urgencias, etc.)<br>☐ Ambiente residencial denso (hogar de ancianos, otro centro de asistencia de larga duración)<br>☐ Prisión o cárcel<br>☐ Establecimiento de envasado de carne<br>☐ Establecimiento de envío o distribución<br>☐ Establecimiento minorista de alto volumen (tienda de provisiones, etc.)<br>☐ No sabe
Persona 12: ¿Le ofrece el empleador a esta persona alguno de los siguientes beneficios en su empleo principal actual?	☐ Permiso por enfermedad con goce de sueldo<br>☐ Vacaciones o permiso personal con goce de sueldo<br>☐ Seguro de salud<br>☐ Seguro de discapacidad<br>☐ Plan de jubilación<br>☐ Otro<br>☐ No sabe<br>((Seleccione todo lo que corresponda.))
Persona 12: En una escala de 0 (definitivamente no va a suceder) a 10 (definitivamente va a suceder), ¿qué tan probable es que esta persona pierda su empleo debido a la pandemia de COVID-19?	______________________
Persona 12: En una escala de 0 (definitivamente no va a suceder) a 10 (definitivamente va a suceder), ¿qué tan probable es que a esta persona se le asignen menos horas de trabajo debido a la pandemia de COVID-19?	______________________

	Todo el tiempo (100%)	La mayor parte del tiempo (75%)	La mitad del tiempo (50%)	Menos de la mitad del tiempo (25%)	nunca (0%)	Nunca
Persona 12: Actualmente ¿con qué frecuencia se le exige a esta persona que trabaje fuera de su residencia?	◯	◯	◯	◯	◯	◯

Persona 12: Actualmente, ¿con qué frecuencia se encuentra esta persona físicamente cerca de sus compañeros de trabajo mientras trabaja fuera de su residencia? ◯ ◯ ◯ ◯ ◯ ◯

Persona 12: Actualmente, ¿con qué frecuencia se encuentra esta persona físicamente cerca de sus clientes mientras trabaja fuera de su residencia ◯ ◯ ◯ ◯ ◯ ◯

Persona 12: ¿Piensa esta persona vacunarse contra la COVID-19 cuando se ofrezca una vacuna?

◯ Sí
◯ No
◯ No sabe

Persona 12: En las últimas dos semanas, ¿ha tenido esta persona algún síntoma de COVID-19 (tos, fiebre, dificultad para respirar, fatiga, dolores de cuerpo, diarrea, goteo nasal o pérdida del sentido del olfato o del gusto)?

◯ Sí
◯ No
◯ No sabe

Persona 12: ¿Cuándo le comenzaron los síntomas de COVID-19 a esta persona?

____

Persona 12: En vista de sus síntomas, ¿le preocupa a esta persona la posibilidad de tener COVID-19?

◯ Sí
◯ No
◯ No sabe

Persona 12: ¿Fue esta persona objeto de prejuicio o discriminación debido a sus síntomas?

◯ Sí
◯ No
◯ No sabe

Persona 12: ¿Qué hizo esta persona en vista de sus síntomas?

☐ Nada
☐ Tomó medicamentos sin receta (ibuprofeno, acetaminofén, etc.)
☐ Consultó por teléfono a un proveedor de asistencia médica
☐ Fue al consultorio de un proveedor de asistencia médica
☐ Fue a una clínica o una farmacia minorista
☐ Fue a un centro de urgencias (FASTMed, etc.)
☐ Fue a la sala de emergencias
☐ Fue ingresado al hospital
☐ Otra cosa
☐ No sabe
((Seleccione todo lo que corresponda.))

Persona 12: ¿Qué otra cosa hizo esta persona en vista de sus síntomas?

____

Persona 12: ¿Le dijo un proveedor de asistencia médica a esta persona que era posible que tuviera COVID-19?

◯ Sí
◯ No
◯ No sabe

Persona 12: Si a esta persona le hicieron una prueba de COVID-19 en vista de sus síntomas, ¿cuál fue el resultado?	◯ Pendiente<br>◯ Positivo<br>◯ Negativo<br>◯ No concluyente<br>◯ No se hizo la prueba<br>◯ No sabe
Persona 12: ¿Cuántos días estuvo hospitalizada esta persona?	__________________________________
Persona 12: ¿Se le hicieron a esta persona las siguientes intervenciones durante su hospitalización?	☐ Oxígeno adicional por la nariz<br>☐ Tratamiento en la Unidad de Cuidados Intensivos (Intensive Care Unit, ICU)<br>☐ Ventilación mecánica (intubación o tubo de respiración)<br>☐ No sabe
Persona 12: ¿Ha vuelto esta persona a su salud normal?	◯ Sí<br>◯ No<br>◯ No sabe
Persona 12: ¿Cuáles de las siguientes medidas tomó esta persona para proteger a sus amigos y familiares después de que comenzaron sus síntomas?	☐ Ponerse mascarilla con mayor frecuencia<br>☐ Lavarse las manos con agua y jabón con mayor frecuencia<br>☐ Limpiarse las manos con desinfectante con mayor frecuencia<br>☐ Aislarse en su casa con mayor frecuencia<br>☐ Quedarse en casa con mayor frecuencia<br>☐ Ponerse guantes desechables con mayor frecuencia<br>☐ No sabe

Complete la siguiente informacion sobre su salud mental y bienestar

A su juicio, ¿qué tan grave es para usted personalmente la pandemia de COVID-19 en este momento?	◯ Muy grave<br>◯ Más o menos grave<br>◯ No demasiado grave<br>◯ Nada grave
A su juicio, ¿qué tan grave es actualmente la pandemia de COVID-19 para los integrantes de su comunidad?	◯ Muy grave<br>◯ Más o menos grave<br>◯ No demasiado grave<br>◯ Nada grave
A su juicio, ¿qué tan grave es actualmente la pandemia de COVID-19 para los habitantes de Estados Unidos?	◯ Muy grave<br>◯ Más o menos grave<br>◯ No demasiado grave<br>◯ Nada grave
A su juicio, ¿qué tan grave es actualmente la pandemia de COVID-19 para los habitantes de todo el mundo?	◯ Muy grave<br>◯ Más o menos grave<br>◯ No demasiado grave<br>◯ Nada grave
En las últimas dos semanas, ¿con qué frecuencia ha encontrado usted medios de comunicación, hechos o tendencias virales, conversaciones en línea, fotos o videos en línea en los cuales una persona como usted es amenazada o lastimada?	◯ Nunca<br>◯ Aproximadamente una vez al mes<br>◯ Aproximadamente una vez a la semana<br>◯ Aproximadamente una vez al día<br>◯ Varias veces al día

En las últimas dos semanas, ¿con qué frecuencia ha encontrado usted medios de comunicación, hechos o tendencias virales, conversaciones en línea, fotos o videos en línea en los cuales se presentan estereotipos negativos de personas como usted como si fueran verdaderos?

- ◯ Nunca
- ◯ Aproximadamente una vez al mes
- ◯ Aproximadamente una vez a la semana
- ◯ Aproximadamente una vez al día
- ◯ Varias veces al día

La pandemia de COVID-19 puede cuasar desafios por algunas personas sin importar a que esten infectados. Que tan preocupado/a esta usted sobre cada una de la siguiente?

	Nada	No mucho	Un poco	Mucho
Recibir la asistencia médica que necesita (incluyendo la asistencia de salud mental)	◯	◯	◯	◯
Tener un lugar para vivir	◯	◯	◯	◯
Poder interactuar con otras personas	◯	◯	◯	◯
Conseguir comida, agua y otros suministros domésticos	◯	◯	◯	◯
Conseguir medicamentos	◯	◯	◯	◯
Tener transporte para llegar adonde tiene que ir	◯	◯	◯	◯
Cuidar de su familia y a sus amigos	◯	◯	◯	◯

Durante las ultimas 2 semanas, que tan seguido ha tenido molestias debido a los siguientes problemas?

	Nunca	Varios días	Más de la mitad de los días	Casi todos los días
Nerviosismo o ansiedad	◯	◯	◯	◯
Incapacidad de dejar de preocuparse o controlar las preocupaciones	◯	◯	◯	◯
Exceso de preocupación por diferentes cosas	◯	◯	◯	◯
Dificultad para tranquilizarse	◯	◯	◯	◯
Tanta inquietud que le es difícil estarse quieto	◯	◯	◯	◯
Facilidad para molestarse o irritarse	◯	◯	◯	◯
Temor de que algo horrible podría pasar	◯	◯	◯	◯

¿Cuánta dificultad le han causado estos problemas para hacer su trabajo, ocuparse de las cosas de su casa o llevarse bien con los demás?

- ◯ Nada de dificultad
- ◯ Un poco de dificultad
- ◯ Bastante dificultad
- ◯ Mucha dificultad

Durante las ultimas 2 semanas, que tan seguido ha tenido los siguentes sentimientos y comportamientos?

	Rara vez o nunca (menos de 1 día)	Pocas veces (1-2 días)	De vez en cuando (3-4 días)	La mayor parte del tiempo o todo el tiempo (5-7 días)
Me ha molestado lo que normalmente no me molesta.	◯	◯	◯	◯
No he tenido ganas de comer; he tenido poco apetito.	◯	◯	◯	◯
He sentido que no podía librarme de la tristeza, ni siquiera con la ayuda de mis familiares o amigos.	◯	◯	◯	◯
He sentido que yo no soy tan bueno como otras personas.	◯	◯	◯	◯
Me ha costado concentrarme en lo que hacía.	◯	◯	◯	◯
Me he sentido deprimido.	◯	◯	◯	◯
He sentido que todo lo que hacía implicaba un gran esfuerzo.	◯	◯	◯	◯
He sentido desesperanza respecto al futuro.	◯	◯	◯	◯
He pensado que mi vida es un fracaso.	◯	◯	◯	◯
He tenido miedo.	◯	◯	◯	◯
No he dormido bien.	◯	◯	◯	◯
No he sido feliz.	◯	◯	◯	◯
He hablado menos de lo habitual.	◯	◯	◯	◯
Me he sentido solo.	◯	◯	◯	◯
Las personas han sido poco amistosas.	◯	◯	◯	◯
No he disfrutado de la vida.	◯	◯	◯	◯
He tenido episodios de llanto.	◯	◯	◯	◯
Me he sentido triste.	◯	◯	◯	◯
He sentido que no les agrado a los demás.	◯	◯	◯	◯
No he podido "ponerme en marcha".	◯	◯	◯	◯

¿Cómo llenó esta encuesta?

◯ En una computadora (portátil o de escritorio)
◯ En un dispositivo móvil (tableta o teléfono celular)
◯ Por teléfono con un entrevistador
◯ Otra manera
