# Supplementary material for: SARS-CoV-2 infection in central North Carolina: Protocol for a population-based longitudinal cohort study and preliminary participant results
Source: PLoS One. 2021 Oct 25;16(10):e0259070. doi: 10.1371/journal.pone.0259070 (PMC8544868; doi:10.1371/journal.pone.0259070)
Supplement: S3 Appendix — (DOCX) [file pone.0259070.s006.docx]

**C4 COVID Study Laboratory Protocol**

Table of Contents

[**Specimen Collection Details and Laboratory Preparation** 1](#_Toc67517392)

[**Solutions for Sample Processing** 2](#_Toc67517393)

[**Processing of Mid-Turbinate Nasal Swab (MTNS)** 3](#_Toc67517394)

[**Processing of Blood Tubes** 4](#_Toc67517395)

[**Processing of Tasso Serum Sample** 7](#_Toc67517396)

[**Enzyme-linked immunosorbent assay (ELISA) Protocol** 8](#_Toc67517397)

## **Specimen Collection Details and Laboratory Preparation**

Protocol based on collection of 2 Ficoll tubes and 1 MTNS. MTNS collected by participants at home between clinic visits will be brought to participants’ subsequent clinic visits; these MTNS specimens will be processed and tested for SARS-CoV-2 by the study team using RNA Extraction and PCR as outlined in this protocol. MTNS collected during clinic visits will be sent to the North Carolina State Laboratory for Public Health for SARS-CoV-2 PCR testing. A Tasso serum separator tube (SST) may be collected if participants have opted into at-home specimen collection. Study staff will leave Ficoll and Tasso tubes at room temperature in a BSL2 hood and MTNS samples in the receiving refrigerator at 4°C.

**Before Starting:**

- **Turn on water bath to 56**°**C.**
- **Prepare 10% bleach solution in waste bucket - place waste bucket inside hood.**
- **Print labels based on guideline below.**

Each label should include:

- Patient ID and Month Number
- Date of collection
- Sample type (S- serum, SW- swab (if applicable), PBMC- PBMC cells, SST- Tasso)

Ensure all steps are carried out in BSL2 hood unless indicated. All staff must wear double gloves, a lab coat, eye protection, and closed toe shoes.

All spills to be cleaned per laboratory protocol.

Any exposure to biologic material should be reported to Occupational Health and the PI.

## **Solutions for Sample Processing**

Use an autoclaved, sterile glass bottle for making the amount you will need for the week.

Use aseptic technique and sterile pipettes when making reagent.

Discard and remake the reagent after one week.

Store reagent at 4°C for the week.

**PBMC Freezing Medium 1 (prepare a master mix based on these per participant volumes)**

1ml FBS (Life technologies 10082147)

1.5ml RPMI 1640 (Invitrogen 11875119)

Store at 4°C for up to one week.

**PBMC Freezing Medium 2 (prepare a master mix based on these per participant volumes)**

0.5ml DMSO (Sigma D8418-500ml)

2ml RPMI 1640

Store at 4°C for up to one week.

## **Processing of Mid-Turbinate Nasal Swabs (MTNS)**

**Equipment needed**

- Sample in BSL2 hood
- 2 inch Storage Box
- 1ml pipette
- 2 X 2ml O-ring tubes
- 2 printed labels for each sample
- Pipettor
- Vortex
- Racks for tubes
- Qiagen Viral RNA Mini Kit

**Sample Processing**

1. Check patient identifiers
2. Label 2 X 2ml O-ring tubes with patient ID
3. Vortex the tube with swab for 10-15 seconds
4. Let tube stand for 5 minutes in a rack
5. Remove cap
6. Use sterile 1ml pipette to pipet 0.5ml of solution into each of 2 tubes.
7. Dispose of tube with swab and cap in biohazard waste.
8. Place the 2 X 2ml O-ring tubes in a storage box in -80°C freezer.

**Extraction & PCR Testing**

- RNA is extracted from stored swab samples according to the QIAamp® Viral RNA Mini Kit Handbook.^3^

**PCR Protocol**

- Extracted RNA is analyzed according to TaqPath™COVID‑19 Combo Kit protocol.^4^

## **Processing of Blood Tubes**

**Equipment needed**

- Sample
- 2 inch Storage Boxes
- Eppendorf 5702 and Sorvall ST16R centrifuges
- Up to 5 X 2ml O-ring sample storage tubes
- 2 X 10ml pipettes
- 2 X 5ml pipettes
- 1 X 15ml Falcon conical tube
- Serologic Pipette device
- Biohazard bag in BSL2 hood
- Sample labels
- Blood tube racks
- 200µl pipette tips
- 200µl pipettemen
- Phosphate Buffered Saline (PBS) (Sigma P5493-1L)
- Water bath at 56°C
- Up to 10 X cryovials
- 1 X 15ml conical tube
- 20µl pipettor tips
- 20µl pipettor
- 3 X 5ml pipettes
- 1 X 10ml pipette
- PBMC Freezing medium 1 (see “solutions for sample processing”)
- PBMC Freezing medium 2 (see “solutions for sample processing”)
- Disposable cell counter slide for Nexcelom Auto 2000 (SD100)
- Mr Freezy (Thermo Fisher 5100-0001)
- Isopropyl alcohol
- 0.4% trypan blue stain (Invitrogen T10282)
- 0.65ml centrifuge tubes
- 1ml pipette tips

**Plasma Processing and PBMC Collection**

1. Use the two Ficoll tubes for this sample processing. **(Processing should start within 2 hours of collection.)**
2. Record processing start time on the Sample Tracking spreadsheet.
3. Centrifuge the two Ficoll tubes at 1,600 X g for 30 minutes at 22°C using the Sorvall ST16R centrifuge **(during this time, can process the nasal swab as above)**.

**Plasma Collection from Ficoll Tubes**

1. Label up to 5 X 2ml O-ring tubes with patient sample label.
2. Label 1 X 15ml Falcon tube with patient sample label.
3. Remove plasma (top clear yellow layer) from each Ficoll tube into a 15ml Falcon tube using a 10ml pipettor. **Do not disturb the hazy layer below. You may leave some plasma.**
4. Confirm caps of 15ml Falcon tubes are on tight.
5. **Store tubes of plasma at 4°C and move to step 1 of PBMC Collection from Ficoll Tubes. (Return to completing plasma clean-up during the first PBS wash of PBMC Collection process.)**
6. Heat inactivate the plasma for 30 minutes at 56°C, mixing every 5 minutes.
7. Bring 15ml Falcon tube to Eppendorf 5702 centrifuge and spin 1,500Xg for 10 minutes to pellet red blood cells and protein aggregate.
8. Bring back to BSL2 hood.
9. Carefully transfer 1.5ml of the supernatant (plasma) using a 2ml pipette into each of two 2-ml sterile tubes with screw top lids with O-rings using a sterile pipette tip.
10. Cap these tubes.
11. Place the two plasma-containing vials into storage boxes in the -80°C freezer.
12. Enter location of the samples into the FreezerPro tracking system.

**PBMC collection from Ficoll Tubes^5^**

1. Label a 15ml Falcon tube with patient ID.
2. Label up to 10 cryovials with patient ID.
3. Transfer the remaining plasma and the whitish, mononuclear layer (everything above the gel layer) using a serological pipette, to a 15ml Falcon tube, pooling the mononuclear layer from each of the two Ficoll-containing vacutainers into one conical tube per participant.
4. Add **1x PBS** to bring the total volume in the conical tube to 15 ml.
5. Cap tube and invert 5 times.
6. **Centrifuge (with brake and acceleration OFF - Acc 9, Dec 1) for 20 minutes at 500 x g, 22°C.**
7. Return the conical tube to the hood and aspirate all but ~500 µl of the PBS without disturbing the pellet. (PBMC yield is greater if ~200 µl of PBS is left above the pellet at this stage).
8. Add fresh **1x PBS** to bring the volume to 10 ml. Resuspend the pellet gently. Cap the tube and invert 5 times. **Centrifuge (with brake and acceleration OFF - Acc 9, Dec 1) for 20 minutes at 500 x g, 22°C.**
9. Aspirate as much supernatant/PBS as possible without disturbing the pellet. Resuspend pellet by adding in 1.5ml PBMC Freezing Medium 1.
10. Add 1.5ml PBMC Freezing Medium 2 to the cell/medium solution. Vortex gently.
11. Aliquot 10 µl of the cell solution into a 0.65 ml microcentrifuge tube. Add 10 µl of 0.4% trypan blue stain into the 0.65 ml microcentrifuge tube and mix by pipetting several times.
12. Pipette 10 µl of the mixture into a cell counting chamber slide (SD100 Cellometer) and place slide into the cell counter within 3 minutes of mixing. Zoom in and focus the cells. Press the “Count Cells” to obtain PBMC count.
13. *If the viable PBMC number is between 3 and 6 million live cells per milliliter (mc/ml),* aliquot 1mL into each cryovial. Store PBMCs in up to 10 cryovials at a concentration of at least 3 mc/ml each.
14. *If the viable PBMC number is above 6 million cells per milliliter,* add equal parts of Freezing Medium 1 and Freezing Medium 2 to get concentration to be between 3 and 6 mc/ml. Aliquot 1mL into each cryovial.
15. *If the viable PBMC number is below 3 mc/ml*, calculate the total number of cells using an excel spreadsheet calculator *or* by multiplying the viable mc/ml by 3 ml (which is the total volume the cells are in at this time). Determine a final volume so that the concentration is at least 3mc/mL.
    1. Centrifuge the conical tube containing the cells/freezing medium solution for 5 minutes at 300 x g (brake and acceleration OFF). After centrifugation, aspirate the appropriate volume of freezing medium (supernatant) so the amount remaining is the final volume calculated above.
    2. Resuspend the pellet in the remaining supernatant and aliquot at least 3 mc/ml into the appropriate number of cryovials (1-4) at 1 ml/cryovial. Final freezing medium is 10% Dimethyl Sulfoxide (DMSO)/20% Fetal Bovine Serum (FBS)/70% Roswell Park Memorial Institute (RPMI) 1640.
    3. Document the cell count per cryovial - number the cryovials and record the cell concentration for each vial.
16. Transfer the cryovials to a controlled rate freezing container (Mr. Freezy, **must add isopropanol to Mr. Freezy before use**) and store at -80ºC for at least 24 hours after which time the cryovials may be transferred to a cryobox and put in a liquid nitrogen tank (vapor phase) for long-term storage.
17. Enter into FreezerPro tracking system and document processing end time on the Sample Tracking spreadsheet.

## **Processing of Participant-Collected Tasso Serum Samples**

**Equipment needed**

- Sample in biohazard bag
- 3 X patient sample label (2 for nasal swabs, 1 for serum)
  - Label should include: Study ID, date of processing, day number, and type of sample
- Microcentrifuge with swing bucket rotor
- Microcentrifuge tube (0.5mL capacity, freezer-stable, sterile)
- 2 X cryo tubes (freezer-stable, sterile cryovials with O-ring)

**Sample Processing**

1. Remove samples from the biohazard bag inside the BSL2 hood.
2. Label samples with patient labels (confirm that label is the same as on the sample bag).
3. **Blood samples:**
   1. Ensure that the swing bucket rotor is in the Eppendorf 5702 centrifuge. In the BSL2 hood, label a 0.5 mL microcentrifuge tube for each participant.
   2. Use EDTA tubes as adapters for spinning samples.
   3. Spin samples at 1500 xg for 10 minutes. If a balance is needed, use a microcentrifuge tube filled with water inside an EDTA tube adapter.
   4. In the hood, aliquot serum into a labeled 0.5 mL microcentrifuge tube.
   5. Store samples in the study freezer box in a -80°C freezer.

## **Enzyme-linked Immunosorbent Assay (ELISA) Testing**

The SARS-CoV-2 ELISA assay performed on phlebotomy blood samples and Tasso serum samples was developed and validated by the de Silva Laboratory at the University of North Carolina at Chapel Hill. It uses recombinant Spike antigen for neutralizing antibodies for assessing total Ig, IgM, and IgG. Information and assay procedures can be found at <https://immunology.sciencemag.org/content/5/48/eabc8413/>***.***

**References**

1. Birger R, Morita H, Comito D, Filip I, Galanti M, Lane B, et al. Asymptomatic shedding of respiratory virus among an ambulatory population across seasons. mSphere. 2018 Jul 11;3(4):e00249-18. doi: 10.1128/mSphere.00249-18.
2. Galanti M, Birger R, Ud-Dean M, Filip I, Morita H, Comito D, et al. Longitudinal active sampling for respiratory viral infections across age groups. Influenza Other Respir Viruses. 2019 May;13(3): 226-232. doi: 10.1111/irv.12629.

3. Qiagen. QIAamp® Viral RNA Mini Kit handbook [Internet]. 2020 [cited 2021 Apr 21]. Available from: <https://www.qiagen.com/us/resources/download.aspx?id=c80685c0-4103-49ea-aa72-8989420e3018&lang=en>.

4. Thermo Fisher Scientific. TaqPath™ COVID‑19 Combo Kit instructions for use [Internet]. 2021 [cited 2021 Apr 21]. Available from: <https://assets.thermofisher.com/TFS-Assets/LSG/manuals/MAN0019181_TaqPath_COVID-19_IFU_EUA.pdf>

5. Weckle A, Aiello AE, Uddin M, Galea S, Coulborn RM, Soliven R, et al. Rapid fractionation and isolation of whole blood components in samples obtained from a community-based setting. J Vis Exp. 2015 Nov 30;(105):e52227. doi: 10.3791/52227
