# Supplementary material for: SARS-CoV-2 infection in central North Carolina: Protocol for a population-based longitudinal cohort study and preliminary participant results
Source: PLoS One. 2021 Oct 25;16(10):e0259070. doi: 10.1371/journal.pone.0259070 (PMC8544868; doi:10.1371/journal.pone.0259070)
Supplement: Attachment — Submitted filename: R2R1.docx [file pone.0259070.s008.docx]

September 17, 2021

Emily Chenette

Editor-in-Chief

*PLOS ONE*

Dear Dr. Chenette:

Thank you for your review of our manuscript entitled, “**SARS-CoV-2 infection in central North Carolina: Protocol for a population-based longitudinal cohort study and preliminary participant results.”** We appreciate the constructive comments of the reviewers, which we believe have significantly improved the manuscript. Please find our responses (in blue) to comments below:

Reviewer #1

**Major Comments**

- None

**Minor Comments**

- Sentences from 109-111 and 116-118 are redundant. Remove one of them.
  - We have removed one of the redundant sentences: *“Study protocols were reviewed by the University of North Carolina at Chapel Hill Institutional Review Board. Initial approval (Study #20-1632) was granted under expedited review under 45 CFE 46.110 on June 10, 2020. Written informed consent was obtained from all participants.”* (Lines 116-118)
- S1 figure has no label.
  - The figure title “*Map of North Carolina counties highlighting Chatham County”*(Line 536) explains what the map is showing; we request this line to be printed with the final published figure.
- Figure 2 resolution is not good.
  - We have re-attached a high-quality version of S2 Figure. Informational postcard describing study.
- Page 251 and 259 µl not ul. Change it throughout the manuscript if there are more.
  - Changed.
- It is not clear why there are multiple rows in Table 1.
  - In order to make the categorization more explicit, we have re-formatted **Table 1** *“Distinction between natural immunity from SARS-CoV-2 infection and acquired immunity from vaccination, based on vaccination status, ELISA RBD antibody assay, and Abbott Architect SARS-CoV-2 IgG assay targeting the nucleocapsid protein.”*
  - We have added an additional column title “possible combination” and added shading to the rows. **Please see tables below demonstrating the change.**

Submitted layout of Table 1:

Revised layout of Table 1:

Reviewer #2

**Major Comments**

- None

**Minor Comments**

- Please see comments to improve and emphasize this is a preliminary report in the title and in the text early on that the study is ongoing so the reader is crystal clear on that.
  - We added clarifying language to indicate that the study is ongoing in the introduction and abstract:
    - The title (Line 4) now reads: *“SARS-CoV-2 infection in central North Carolina: Protocol for a population-based longitudinal cohort study* ***and preliminary participant results****”*
    - The abstract (Line 48) now reads: “This **ongoing** prospective, observational study aimed to generate accurate estimates”
- Will [results] be eventually or real-time compared with full, accrual, reported population incidence/prevalence by state health authorities?
  - We added a description of our data sharing practices with the state health authorities into the abstract (Line 54): ***“Study data are being shared on a monthly basis with North Carolina state health authorities and future analyses aim to compare study data to state-wide metrics over time.”***
- Add plan for dissemination of findings
  - We have added details on dissemination to the state health authorities as part of the line edited in the previous comment (Line 54).
- Is there a table where [the demographic similarity of Chatham County and North Carolina] is compared?
  - This has been included in the original submission in the supporting information section (table S1).
- Add sampled [#] in [area] population
  - We have included more details about the population where our cohort came from and the sentence (line 53) now reads: *“As of June 23, 2021, we have enrolled a total of 153 participants* ***from a county with an estimated 76,285 total residents.”***
- Add “or natural immunity” (i.e. from exposure) (line 68)
  - We have revised the sentence to read: *“Until vaccination rates* ***or “natural immunity” from exposure*** *reach critical thresholds, guidance on the scope and duration of restrictions will continue to require epidemiological measurements of community infections.”*
- Add “or, in lay terms…,” change the word “assay” to “test,” (line 72) science changes very rapidly.
  - We have revised the relevant sentence, which now reads: *“Current estimates of SARS-CoV-2 incidence, prevalence across geographic regions, and mortality rates are largely drawn from seroprevalence studies [7],* ***which measure antibodies against the virus found in blood samples****. These studies vary by design, serological* ***test*** *employed****…”***
- Add citation (line 79)
  - We have inserted a new reference [9] and renumbered the remaining citations:
    - “The seroprevalence of SARS-CoV-2 is rapidly changing in North Carolina **[9]**…”
    - **9. Lopez CA, Cunningham CH, Pugh S, Brandt K, Vanna UP, Delacruz MJ, et al. Disparities in SARS-CoV-2 seroprevalence among individuals presenting for care in central North Carolina over a six-month period. medRxiv [Preprint]. 2021 Mar 30. doi: 10.1101/2021.03.25.21254320**
- Add “and have instead relied on____” (line 78)
  - We have added clarifying language to Line 82 to explain how past studies have often sampled populations: “However, no studies to date have used representative population estimation methods **and often rely on convenience sampling, which is difficult to extrapolate to underlying populations** [7].”
- Replace “examine” with “estimate and examine a truer population-based…” (line 93)
  - We have revised the sentence to read: *“Here, we describe the protocol of an ongoing study that was designed* ***to estimate and examine a truer population-based*** *incidence and prevalence of SARS-CoV-2 infection in a representative sample of adults residing in one county in central North Carolina.”*
- Add in spoilage rate for samples
  - We have added in data on rates that nasal swabs were not able to be analyzed by the State Lab of Public Health and rates that Tasso-based blood samples were not usable by our lab team (lines 404-408): ***“The majority of samples collected have successfully been analyzed; 0.8% (9 out of 1069) of nasal swabs collected for PCR testing by the North Carolina State Lab of Public Health were unusable due to shipping delays, damage to the materials, or not enough sample collected. 16.5% (20 out of 121) of samples from Tasso devices were not usable due to participant error or too small of volume of blood collected, or other causes.”***
- Cite news article on impact of essential workers (line 433)
  - We have added the following a reference to the following text (Line 433): *“In addition, rural communities host major agricultural production facilities with many workers deemed “essential”* ***[36].”***
    - **36. Sánchez-Guerra, A., 2020. ‘We’re not disposable’: Agricultural workers demand COVID-19 protections from state. News & Observer, [online] Available at: <https://www.newsobserver.com/news/coronavirus/article246984187.html> [cited 2021 September 10].**
- Other grammatical edits:
  - Add “one county” (line 86)
  - Add “more population-based” (line 88)
  - Insert “see” before referring to figure (various lines)
  - Write out phase number (e.g. “1” should be “One”) (various lines)
  - Move line 110 to line 126
  - Add county health department name (line 122)
  - Move line 193 up in paragraph
  - Break up Phase One and Phase Two explanations (line 143)
  - Clarify wording around exclusion of some high-risk individuals (line 360)

*We have incorporated all grammatical edits listed above.*

Thank you for your continued consideration of our manuscript.

Kind regards,

Ross M. Boyce MD, MSc

Assistant Professor

Division of Infectious Diseases
